# Sputum colour charts to guide antibiotic self-treatment of acute exacerbation of chronic obstructive pulmonary disease: the Colour-COPD RCT

**DOI:** 10.1136/bmjresp-2025-003615

**Published:** 2025-10-10

**Authors:** Eleni Gkini, Joshua De Soyza, Daniella A Spittle, Paul Robert Ellis, Sarah Tearne, Peymane Adab, Rachel Jordan, Nawar Diar Bakerly, Alice Margaret Turner

**Affiliations:** 1 Birmingham Clinical Trials Unit, College of Medicine and Health, University of Birmingham, Birmingham, UK; 2 University Hospitals Birmingham NHS Foundation Trust, Birmingham, UK; 3 School of Medical Sciences, College of Medicine and Health, University of Birmingham, Birmingham, UK; 4 School of Health Sciences, College of Medicine and Health, University of Birmingham, Birmingham, UK; 5 Northern Care Alliance NHS Foundation Trust, Salford, UK; 6 Manchester Metropolitan University, Manchester, UK

**Keywords:** COPD Exacerbations, Pulmonary Disease, Chronic Obstructive

## Abstract

**Background:**

Chronic obstructive pulmonary disease (COPD) patients are encouraged to manage exacerbations (acute exacerbation of COPD (AECOPD)) through self-management (SM) plans. Since only around half of AECOPD are bacterial, and sputum colour correlates with bacterial load, it may help guide antibiotic use. This pragmatic randomised controlled trial (RCT) assessed the safety and effectiveness of using a sputum colour chart in UK primary care.

**Methods:**

The multicentre RCT, Colour COPD randomised COPD adults who had ≥2 AECOPD or ≥1 AECOPD hospital admission in the preceding year. The primary objective was to assess the non-inferiority of the Bronkotest sputum colour chart compared with usual care, with hospital admission for AECOPD at 12 months as the primary outcome. Secondary outcomes included second courses of treatment requirement and quality of life (CAT score). Nested substudies examined daily symptoms via e-diaries and sputum culture.

**Results:**

115 severe COPD patients (global obstructive lung disease(GOLD) D, 54% Medical Research Council (MRC) 4 or 5, CAT score 24) were randomised. A trend towards more hospital admissions (32% vs 16%, relative risk (RR) 1.95 (0.92–4.18)) and increased antibiotic use within 14 days (34% vs 18%, adjusted relative risk (aRR) 1.80 (0.85–3.79)) was seen in the colour chart group. From 38 sputum substudy patients, 57 samples were received (42 stable, 15 during AECOPD), with 30% containing potentially pathogenic bacterium (PPB). Purulent sputum was more frequent in bronchiectasis, independent of disease state (stable vs exacerbation) or PPB presence, suggesting sputum colour alone does not reliably guide antibiotic use.

**Conclusion:**

Under-recruitment precluded definitive conclusions. However, sputum colour is unlikely to be a useful addition to COPD SM in primary care.

**Trial registration number:**

The UK’s Clinical Study Registry: ISRCTN14955629 (https://doi.org/10.1186/ISRCTN14955629; registration date: 11 Number 2020).

**WHAT IS ALREADY KNOWN ON THIS TOPIC**

- Sputum colour relates closely to bacterial load, hence may be a useful tool to aid patients in choosing which part of a rescue pack of antibiotics and steroids to use when self-managing a chronic obstructive pulmonary disease (COPD) exacerbation. However, this has not been tested in a robust clinical trial.

**WHAT THIS STUDY ADDS**

- This randomised controlled trial of self-management with a colour chart and self-management without a colour chart did not show any significant differences in hospitalisations between arms, but there was a trend towards this in the colour chart group, and sputum colour did not relate well to bacterial load in an unselected population. Comorbidity like bronchiectasis may have been a factor.

**HOW THIS STUDY MIGHT AFFECT RESEARCH, PRACTICE OR POLICY**

- Routine use of sputum colour to guide self-management cannot be recommended on the basis of our results, and caution should be exercised if doing so, especially in patients who have comorbid disease.

## Introduction

 Chronic obstructive pulmonary disease (COPD) is a chronic condition affecting 2 million people in the UK, causing over 140 000 hospital admissions and 1.7% of UK hospital bed days per year.^1^ Each year around half of all patients with COPD have frequent exacerbations (acute exacerbation of COPD (AECOPD; ≥2 per year^2^), and in the UK 44%–85% of patients with this AECOPD rate were hospitalised again within 12 months.^3^ Exacerbations are defined by ‘worsening of respiratory symptoms beyond normal day-to-day variations and leading to a change in medication’.^4^ Cardinal symptoms include altered sputum volume and/or colour and worsening dyspnoea.^5^ A systematic review in 2012 found bacteria in just 46% of exacerbations,^6^ suggesting antibiotics will effectively manage only half of AECOPD episodes—nevertheless they are used in the majority of events. Inappropriate antibiotic use or overuse increases the long-term risk of antibiotic resistance^7^ and reducing antibiotic resistance through appropriate stewardship is a recognised priority. In hospitalised AECOPD patients’ resistance occurs in up to 66% of cases and relates to past antibiotic use,^8^ suggesting those with prior hospitalisation or frequent antibiotic courses are a key group to target for interventions aimed at reducing resistance.

Sputum colour is a marker of neutrophilic inflammation and bacterial infection,^9^ suggesting it could be used to guide antibiotic treatment and reduce inappropriate use. In studies of approximately 100 patients over a year,^9^ there was 94% probability that infectious exacerbations of COPD had green sputum (sensitivity of green sputum=94%). Specificity of green sputum for bacterial infection was 77%. This suggested that sputum colour was a tool with potential to reduce inappropriate antibiotic use, and a definitive study to test this was required.

Intuitively, early recognition and treatment of AECOPD would reduce exacerbation severity and duration and improve prognosis; evidence for this is limited but supportive.^10^ This is usually achieved by use of self-management (SM) plans, alongside a pack of antibiotics and steroids (rescue pack (RP)).^11^ Evidence for SM as a means of reducing admission in COPD is inconsistent^12^ and heterogeneous.^13^ Multidisciplinary SM support programmes including action plans for AECOPD management are effective in reducing admissions when they include iterative feedback to patients.^14^ However, a systematic review conducted aimed at delineating the effect of each component of SM found that simple action plans for AECOPD management had no effect on hospital admissions and AECOPD rates.^13^ In the UK, many patients are given an action plan alongside a pack of steroids and antibiotics, which they are advised to use for AECOPD, but often with little education on when and how to use these. Furthermore, UK health professionals have identified numerous training needs to deliver iterative SM.^15^ Given the evidence, current SM plans, in usual care in the UK, are unlikely to reduce hospitalisations. We, therefore, conducted a non-inferiority trial to assess whether a sputum colour chart within a SM plan was safe, reasoning that if it safely reduced antibiotic usage without an increase in treatment failure, including hospital admissions, then this would be the preferred strategy epidemiologically, to reduce long-term antibiotic resistance risks.

## Methods

### Study design and oversight

The Colour-COPD trial was a multicentre randomised controlled trial in primary and secondary care in the UK. Trial steering committee and data monitoring committee oversaw it. The study gained all approvals on 4 November 2020 and the first site opened on 11 November 2021. This reflects the delays in adapting the protocol to operate within early COVID-19 restrictions, as well as the multiple delays in site opening due to diversion of staff into other duties. We had tried to reduce burden on staff from study conduct by way of design, aligning to usual care, and allowing some data collection phone calls to be made by the trials unit instead of general practitioner (GP) practices. We then made further adaptations to improve deliverability during the pandemic—we enabled remote consultation for SM plan education and planned to collect data on how the consultation was conducted—face to face, over video or over the telephone—to assess whether the mode of delivery impacted on efficacy or fidelity. None of the adaptations made a significant impact on recruitment rate. Further details may be found in our report to funder.^16^

### Participants

Participants were adults who had clinically diagnosed COPD, confirmed by a medical record of postbronchodilator spirometry denoting obstruction, and ≥2 AECOPD in the 12 months prior to screening according to the patient or ≥1 hospital admission for AECOPD. Patients were identified in primary care, secondary care outpatient and hospital inpatient settings. They were able to safely use an SM plan and colour chart in the view of their usual care practitioner. To participate in the E-diary substudy, they required access to a smartphone/tablet and an email address. To participate in the sputum substudy, they had chronic bronchitis, defined by self-reported sputum production for at least 3 months in each of 2 consecutive years or more.

### Randomisation

**Figure 1:**
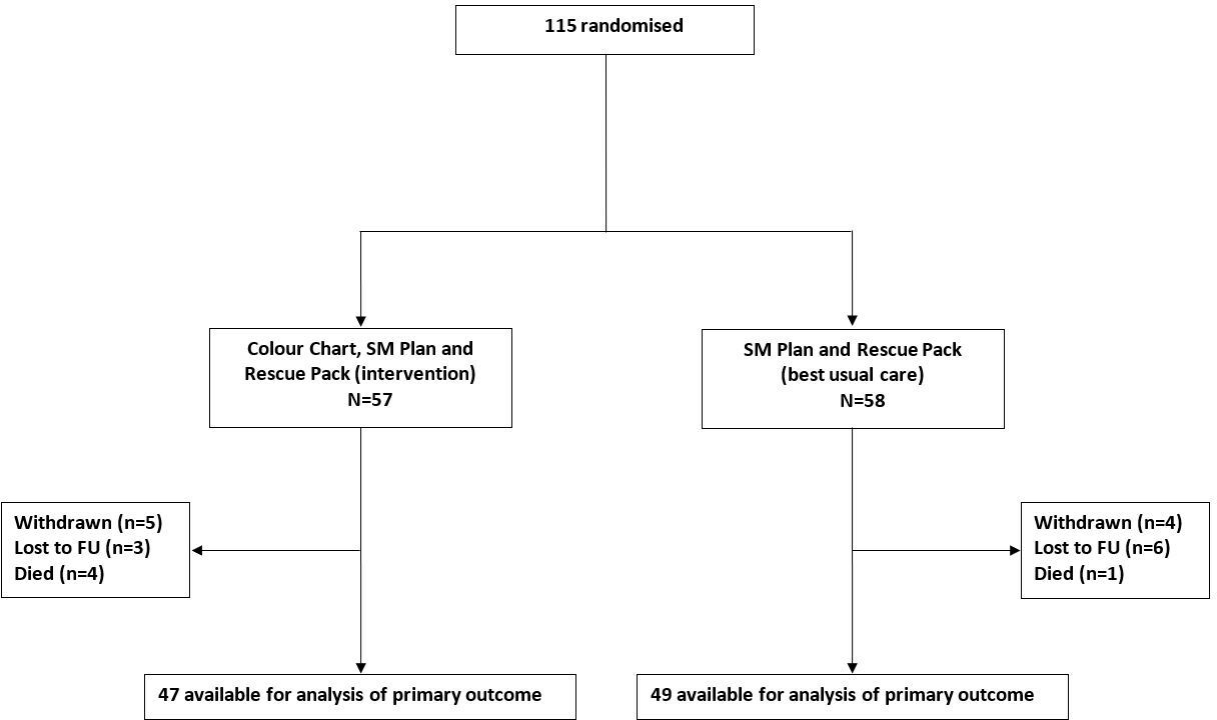
CONSORT diagram. SM, self-management.

Participants were individually randomised by central computer (or telephone if practices had poor online access) in a 1:1 ratio to intervention or control by the Birmingham Clinical Trials Unit to ensure concealment of the next treatment allocation. A minimisation algorithm ensured balance in the treatment for severity of COPD^4^ (global obstructive lung disease (GOLD) C or D), chronic bronchitis (yes or no), COPD hospitalisation in the last year (yes or no) and age (<65 years or 65–80 years or >80 years). In addition, GP practice was included to balance for effect of this. A ‘random element’ was included in the minimisation algorithm, so that each participant had a probability of being randomised to the opposite treatment than they would have otherwise received. Neither the personnel who enrolled participants nor those who assigned them to the interventions had access to the random allocation sequence. The study flowchart is shown in figure 1.

### Intervention and study procedures

The intervention was a 5-point sputum colour chart, adapted from Bronkotest (online supplemental figure S5), used alongside a standardised SM plan and RP containing 5 days of antibiotic and steroid treatment, with patients being told to use their antibiotic component only if their sputum colour changed from baseline or was persistently purulent in the context of other AECOPD symptoms. The control arm used the SM plan and RP alone. Patients were seen at baseline and at 12 months, completing CAT score at both visits, and reporting AECOPD events, treatment and hospitalisations; provision was made for telephone or video appointments to ensure deliverability during the pandemic. A phone call at 2 weeks checked fidelity of delivery of the SM intervention. Patients in the sputum substudy sent sputum at baseline, 12 months and during AECOPD to the central laboratory. Patients in the e-diary substudy completed an e-diary of the EXAcerbations of Chronic pulmonary disease Tool (EXACT) score daily. Further substudy methods are shown in the online supplemental material.

### Outcomes

The primary objective was to assess whether use of the intervention was safe, as defined by non-inferiority compared with use of the plan and pack alone (best usual care) for patient hospitalisation admission for AECOPD at 12 months postrandomisation.

Our secondary objectives included assessing the intervention’s impact on 30-day and 90-day AECOPD readmissions, treatment failure (ongoing symptoms or treatment within 14 days) and time to next AECOPD. We also assessed its effect on reducing self-reported antibiotic use and antibiotics-related adverse events (eg, oral thrush). All exacerbations reported by the patient were captured, whether managed remotely (eg, via telephone consultation) or by community teams; however, detailed healthcare utilisation data collection ceased when the funder mandated early cessation of the trial. The e-diary substudy assessed unreported AECOPD rates. The sputum substudy allowed us to assess the appropriateness of antibiotic use by objectively confirming sputum colour at AECOPD and confirming the presence of potentially pathogenic bacteria (PPB).

### Sample size

Hospitalisation rates from the Clinical Practice Research Datalink (CPRD) informed our sample size calculation.^3^ Assuming a one-sided significance level of 2.5% and a rate of admission in each group of 65% of that in CPRD, with a non-inferiority margin of 6%, we needed to enrol 1329 patients in each of the intervention and control groups to have 90% power. Assuming dropout/lost to follow-up/non-adherence rate of 10%, we therefore needed to recruit 2954 patients. Recruitment challenges led to early study termination.

### Statistical analysis

All primary analyses (primary and secondary outcomes including safety outcomes) were by intention-to-treat. For the primary outcome, frequencies and percentages of participants with at least one AECOPD-related hospital admission were summarised by group and a log-binomial model estimated the risk ratio, risk difference and 95% CIs. Adjusted comparisons using the minimisation variables besides GP practice had convergence issues and were not used. Analyses for readmissions and treatment failure were conducted similarly, but due to lack of events, the number of participants who reported at least one readmission to hospital for AECOPD within 30/90 days was summarised using frequencies and percentages. Total antibiotic and steroid courses were analysed using a negative binomial model, due to evidence of overdispersion identified by the likelihood ratio test, adjusting for minimisation variables as before, apart from GP practice. The natural logarithm of time in years from the date of randomisation to the date of trial last appointment was added as an offset variable to incorporating exposure time. The total CAT and EQ-5D-5L scores at 12 months post-randomisation were analysed individually using a linear regression model adjusting for baseline total scores and minimisation variables, apart from GP practice, as fixed effects. Age at randomisation, number of hospitalisations for COPD in previous year and the corresponding baseline score were included as continuous variables. Distributional assumptions were assessed visually using the studentised regression residuals (online supplemental figures S2 and S3). All other outcomes are reported using descriptive statistics; for details, see the Methods section in online supplemental material. Due to the very small sample size, no sensitivity analyses were performed to assess the potential impact of missing data on the results, nor were any subgroup analyses conducted. Following changes to data collection resulting from the early termination of the trial, it was not possible to verify adherence to the sputum colour chart. The full statistical analysis plan is in online supplemental files 1; 2. The full trial protocol is shown in supplemental file 4

### Patient and public involvement

Patient and public involvement (PPI) was integral to the project from commencement. A PPI group (n=8) was formed in Salford to advise on patient-facing study materials. Additionally, a patient served on our trial steering committee (TSC), and a patient coapplicant was invited to the trial management group (TMG).

## Results

Despite opening more sites than originally planned and shifting to include secondary care, from initially primary care only, recruitment did not improve and closure was mandated by the funder. The last patient enrolled in March 2023 and was followed up until March 2024. figure 1 shows the study flowchart.

Baseline characteristics are shown in table 1. Comorbidity was common with hypertension being present in almost half of participants, approximately 30% of patients having asthma, 25% bronchiectasis and 25% vascular disease (either coronary or cerebral). The majority of patients was not highly educated, and smoke exposure was heavy, though the majority had quit prior to enrolment. At follow-up, number of current smokers was lower in the colour chart group (3 vs 12 in control). Eosinophil counts exhibited a wide range, but the mean was within normal parameters. Symptom and quality of life scores indicated poorly controlled, symptomatic disease, driven by breathlessness. Most patients were taking regular triple therapy (long acting muscarinic antagonist (LAMA)/long acting beta agonist (LABA)/inhaled corticosteroids (ICS)), half were also on mucolytics (online supplemental table S7).

**Table 1:** Baseline characteristics by group and overall

		Allocated treatment		Total
		Colour chart, SM plan and rescue pack (intervention)	SM plan and rescue pack (best usual care)	
		N=57	N=58	N=115
Minimisation variables				
Severity of COPD, n (%)	Category C*	4 (7%)	3 (5%)	7 (6%)
	Category D†	53 (93%)	55 (95%)	108 (94%)
Presence of chronic bronchitis, n (%)		32 (56%)	33 (57%)	65 (57%)
Prior COPD hospitalisations, n (%)		20 (35%)	20 (34%)	40 (35%)
Age groups (years), n (%)	Age<65	17 (30%)	17 (29%)	34 (30%)
	65<=age<=80	33 (58%)	35 (60%)	68 (59%)
	Age>80	7 (12%)	6 (10%)	13 (11%)
Demographic and other baseline variables				
Age at randomisation (years)	Mean (SD)	68.9 (9.2)	68.2 (9.1)	68.5 (9.1)
Gender, n (%)	Male	29 (51%)	34 (59%)	63 (55%)
	Female	28 (49%)	24 (41%)	52 (45%)
Ethnicity, n (%)	White- British/English/Northern Irish/Scottish/Welsh	57 (100%)	56 (97%)	113 (98%)
	Asian and Asian British- Indian	0 (0%)	1 (2%)	1 (1%)
	Black and Black British- African Caribbean	0 (0%)	1 (2%)	1 (1%)
BMI (kg/m²)	Mean (SD)	28.6 (7.8)	26.9 (5.9)	27.7 (6.9)
Education level, n (%)	No formal education	19 (33%)	17 (29%)	36 (31%)
	GCSE, CSE, O level or equivalent	24 (42%)	24 (41%)	48 (42%)
	A-level/AS level or equivalent	6 (11%)	5 (9%)	11 (10%)
	Degree level or higher	7 (12%)	6 (10%)	13 (11%)
	Other please specify	1 (2%)	6 (10%)	7 (6%)
Medical history				
Number of hospitalisations for COPD in previous year	None	37 (65%)	39 (67%)	76 (66%)
	One	8 (14%)	14 (24%)	22 (19%)
	Two	5 (9%)	1 (2%)	6 (5%)
	Three	3 (5%)	0 (0%)	3 (3%)
	More than three	4 (7%)	4 (7%)	8 (7%)
Stable full blood count in the past 12 months		40 (71%)	44 (76%)	84 (74%)
	Missing	1	0	1
Most recent eosinophil level	Mean (SD)	0.18 (0.17)	0.20 (0.15)	0.19 (0.16)
Chronic asthma, n (%)		17 (30%)	20 (34%)	37 (32%)
Bronchiectasis, n (%)		10 (18%)	15 (26%)	25 (22%)
Medical history (ICD-10)				
Diabetes, n (%)		13 (23%)	11 (19%)	24 (21%)
CVA/stroke/TIA, n (%)		2 (4%)	4 (7%)	6 (5%)
Osteoporosis, n (%)		11 (19%)	8 (14%)	19 (17%)
Hypertension, n (%)		23 (40%)	30 (52%)	53 (46%)
Arthritis, n (%)		20 (35%)	19 (33%)	39 (34%)
Coronary heart disease, n (%)		9 (16%)	12 (21%)	21 (18%)
Depression/anxiety, n (%)		21 (37%)	19 (33%)	40 (35%)
GORD, n (%)		14 (25%)	17 (29%)	31 (27%)
Smoking status at baseline				
Current smoking status, n (%)	Current smoker	10 (18%)	15 (26%)	25 (22%)
	Ex-smoker	38 (67%)	40 (69%)	78 (68%)
	Never smoked	9 (16%)	3 (5%)	12 (10%)
Duration of smoking (years)	Mean (SD)	39.3 (13.3, 47)	38.5 (15.8)	38.9 (14.6)
	Missing	1	0	1
Number of cigarettes/day	Mean (SD)	18.2 (9.9)	17.4 (12.5)	17.8 (11.4)
Smoking status at 12 months post randomisation				
Current smoking status, n (%)	Current smoker	3 (7%)	12 (26%)	15 (16%)
	Ex-smoker	34 (76%)	32 (68%)	66 (72%)
	Never smoked	8 (18%)	3 (6%)	11 (12%)
	Missing	12	11	23
Duration of smoking (years)	Mean (SD)	38.8 (13.7)	36.0 (16.7)	37.3 (15.4)
	Missing	1	0	1
Number of cigarettes/day	Mean (SD)	15.0 (9.1)	18.3 (13.0)	16.7 (11.3)
Medical measurement				
FEV_1_: pre-bronchodilator (litres)	Mean (SD)	1.4 (0.7)	1.5 (0.5)	1.4 (0.6)
FEV_1_: post-bronchodilator (litres)	Mean (SD)	1.6 (0.6)	1.5 (0.6)	1.5 (0.6)
MRC Breathlessness Scale, n (%)	Grade 1	1 (2%)	2 (3%)	3 (3%)
	Grade 2	9 (16%)	7 (12%)	16 (14%)
	Grade 3	18 (32%)	16 (28%)	34 (30%)
	Grade 4	16 (28%)	23 (40%)	39 (34%)
	Grade 5	13 (23%)	10 (17%)	23 (20%)
Quality of Life scores				
Baseline CAT score‡	Mean (SD)	23.25 (8.65)	24.48 (7.75)	23.87 (8.19)
Baseline EQ-5D-5L score§	Mean (SD)	0.55 (0.32)	0.56 (0.28)	0.55 (0.30)
Baseline Vas EQ-5D-5L score¶	Mean (SD)	55.4 (22.4)	58.4 (23.1)	56.9 (22.7)

* CAT<10, 2 or more exacerbations in the last 12 months OR 1 hospital admission for an exacerbation.

† CAT≥10, 2 or more exacerbations in the last 12 months OR 1 hospital admission for an exacerbation.

‡ The CAT score can range from 0 to 40. Higher scores indicate that participants’ COPD has a greater impact on their overall health and well-being.

§ The total score EQ-5D-5L was calculated using the mapping function developed by Van Hout *et al*. (2012)^25^ and the Crosswalk value sets for the UK; and it ranges from −0.594 to 1 with −0594 indicates unable to/extreme problems on all of the five dimensions and 1 indicates no problems on any of the five dimensions.

¶ Vas EQ-5D-5L health state scores range from 0 to 100, where higher scores reflect better health.

AS, Advanced Subsidiary; BMI, body mass index; CAT, COPD assessment test; COPD, chronic obstructive pulmonary disease; CSE, Certificate of Secondary Education; CVA, cerebrovascular accident; EQ-5D-5L, EuroQol validity in assessing quality of life; FEV, forced expiratory volume; GCSE, General Certificate of Secondary Education; GORD, gastro-oesophageal reflux disease; ICD, international classification of disease; MRC, Medical Research Council; SM, self-management; TIA, transient ischaemic attack.

Proportions of patients on other relevant treatments were mainly similar between arms except for oxygen and home ventilation, which were more common in the colour chart arm.

**Figure 2:**
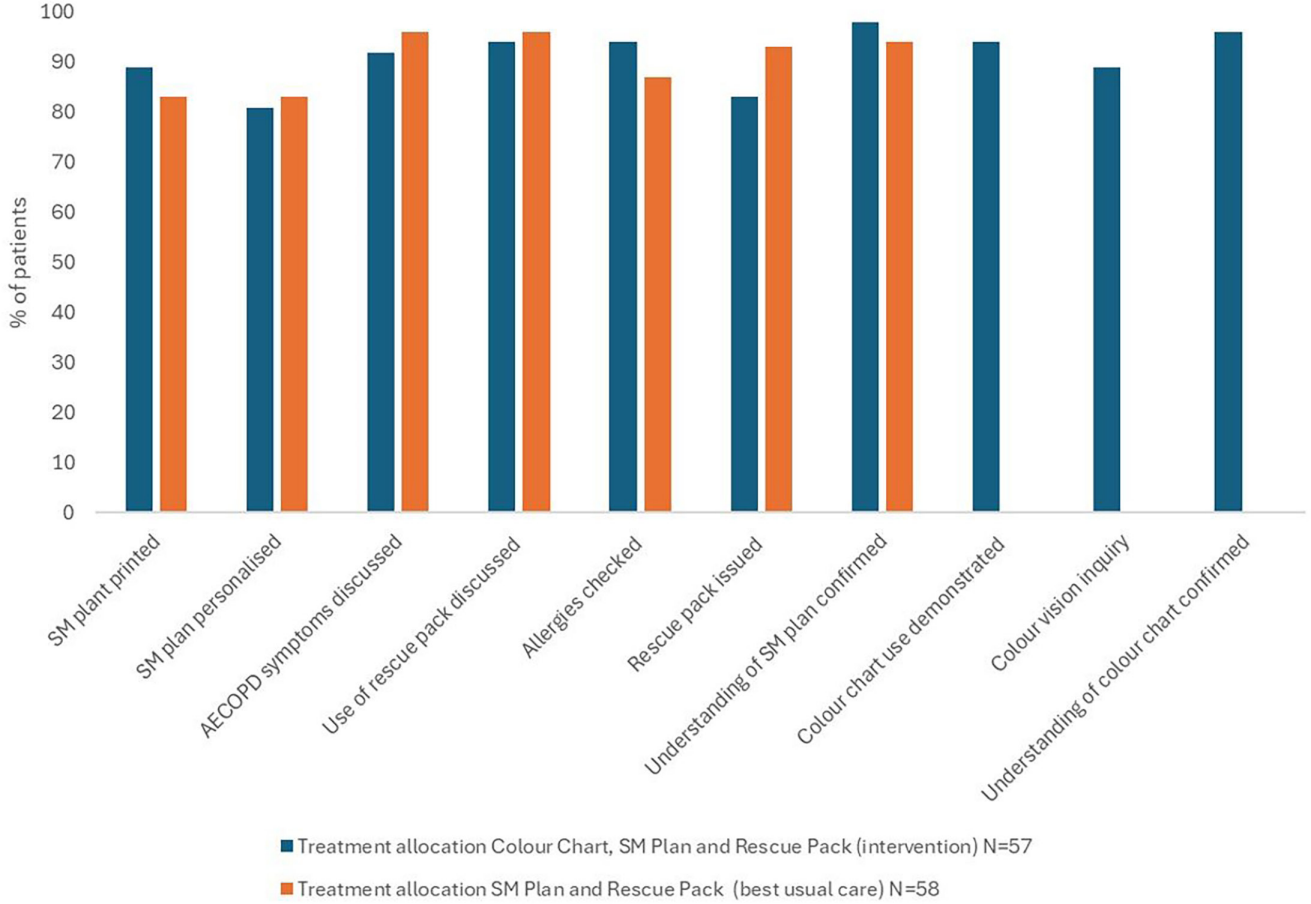
Delivery of key aspects of self-management (SM). AECOPD, acute exacerbation of chronic obstructive pulmonary disease.

SM was delivered consistently and reliably and was largely similar between arms, with the possible exception that RPs were more commonly in the control arm (93% vs 83%), as shown in figure 2.

### Primary outcome

Hospital admissions for AECOPD showed a potential trend towards being higher in the colour chart group compared with usual care (32 vs 16%, relative risk 1.95 (0.92–4.18)), as shown in table 2. Most patients did not experience an admission, and there were three recurrently admitted patients in the colour chart arm (online supplemental figure S1).

**Table 2:** Primary outcome summary statistics

Primary outcome		Allocated treatment			
		Colour chart, SM plan and rescue pack (intervention) N=57	SM plan and rescue pack (best usual care) N=58	Unadjusted relative risk*†‡ (95% CI)	Unadjusted risk difference†‡§ (95%CI)
Number of participants with at least one hospital admission due to AECOPD over 12 months post randomisation	Yes	15 (32%)	8 (16%)	1.95 (0.92 to 4.18)	0.16 (-0.01 to 0.32)
	No	32 (68%)	41 (84%)		
	Missing	10	9		

* RR<1 favours the colour chart, SM plan and rescue pack (intervention).

† Log-binomial regression model.

‡ Adjusted comparisons taking into account all minimisation variables apart from GP practice were performed but resulted in convergence issues and thus were not used.

§ RD<0 favours the colour chart, SM plan and rescue pack (intervention).

AECOPD, acute exacerbation of chronic obstructive pulmonary disease; GP, general practitioner; RD, risk difference; RR, relative risk; SM, self-management.

### Secondary outcomes

Secondary outcome results are summarised in online supplemental tables S1–S3. All results should be interpreted cautiously given the under recruitment. Consistent with the primary outcome, readmissions for AECOPD were higher in the colour chart group, and there was suggestive data for initial treatment failure in this group as well, with more second courses of antibiotics being used. Overall, however, antibiotic courses were no different between the two groups (total course IRR 1.03 (0.73–1.43), at least one course IRR 0.98 (0.82–1.16)). GP visits were rare for COPD in both arms. Counterintuitively, quality of life (CAT score) was better at follow-up in the colour chart group, despite the hints towards worse outcomes from the healthcare utilisation data (19.9 vs 24.5, adjusted mean difference −2.95 (−5.93 to −0.04)).

### Safety

Thirty-four of 51 serious adverse events (SAEs) were expected AECOPD hospitalisations. Non-AECOPD SAEs were largely unexpected and are shown in online supplemental table S4. The reasons were consistent with the age, smoking profile and known literature regarding incidence of other diseases in COPD.

### Substudies

Fifty-seven sputum samples were received from 37 patients: 42 stable, 15 during exacerbation and 17 (30%) contained a PPB. Sputum substudy participants’ characteristics are in online supplemental table S5. Patient-reported sputum colour was available for 54 samples and was similar to the colour reported by lab personnel (p=0.8). In the intervention group, patient-reported colour was similar to the associated number reported from the colour chart (p=0.7). Sputum colour was more likely to be purulent (3–5 on colour chart) in subjects with bronchiectasis, independent of disease state (stable vs exacerbation) or whether the sample was positive for a PPB (table 3). There was no difference in antibiotic resistance rates between arms.

**Table 3:** Odds of purulent sputum sample

Variable	OR*	95% CI
Exacerbation	0.98	0.28 to 1.58
Positive sample for PPB	0.78	0.19 to 3.05
Bronchiectasis	4.41	1.25 to 18.08

* Multiple logistic regression model displaying odds of purulent samples during exacerbation, when positive for a potentially pathogenic bacteria and in those with co-existing bronchiectasis. Intercept (95% CI) = 0.68 (0.28 to 1.58).

PPB, pathogenic bacterium.

Eleven patients enrolled to the e-diary substudy, and 42 symptom-defined AECOPD were detected, many of which were untreated. The median number of exacerbations was 4 (IQR 4.75) with two patients having 10. The annualised exacerbation rate was 9.1 (IQR 2.80) for the control group and 5.8 (IQR 0.86) for the intervention group. Treated exacerbations had a lower baseline symptom score, and a higher rise in symptoms from baseline compared with untreated exacerbations, implying that it was the perceived change that directed therapy, as opposed to the overall severity of symptoms. E-diary participants’ characteristics are shown in online supplemental table S6, and scores for symptom-defined events, stratified by treatment status in online supplemental figure S4.

## Discussion

Overall, the results do not support routine use of colour charts as an adjunct to SM, though caution is needed due to severe under-recruitment. While quality of life was better in colour chart users at 12 months, this could be a spurious result, and is not offset by the potential adverse impact on hospitalisations and treatment failure after initial AECOPD management. The adverse effects may have been a chance finding or resulted from imbalances in the rate of non-invasive ventilation (NIV) and oxygen use, or in the rate of bronchiectasis between arms. Furthermore, the collection of exacerbation data at 12 months based on participants’ self-reported accounts may introduce recall bias.

Despite under-recruitment, trends towards more hospitalisations and second treatment courses in the intervention arm suggest that using a colour chart to decide on antibiotic use in RPs may be inappropriate for this population. The increased hospitalisations, mainly for AECOPD but possibly for other reasons (as shown in SAEs), could have been influenced by small baseline differences in the groups not captured by GOLD COPD severity grading. Colour chart users had higher prevalence of long-term oxygen therapy (LTOT) and home NIV, both indicators of severe disease linked to higher admission rates. Cor pulmonale, also an SAE, was consistent with very severe disease in this group. Unmeasured severity factors such as undiagnosed bronchiectasis, may have been present. Mortality and morbidity among hospital inpatients are known to be high; 1-year mortality is 29%,^17^ and hospitalisation for AECOPD predicts future exacerbations. In a relatively small sample, this factor may have contributed to the adverse effects and high hospitalisation rates seen.

Health-related quality of life (HRQoL) results were counterintuitive; patients felt better despite more hospital admissions and additional treatments. Baseline HRQoL scores were well matched, and our analysis accounted for baseline score and other potential influences on QOL such as disease severity. Given the higher prevalence of very severe disease in the colour chart group, we expected worse HRQoL, but this was not observed. COVID-19 pandemic might have led to greater use of community respiratory services or additional support for patients that was not captured by our medication histories and simple healthcare utilisation measures, and which aided HRQoL. The abandoned economic analysis, due to early study cessation, might have captured this. Alternatively, the colour chart may have boosted patient confidence in symptom recognition, improving HRQoL. The e-diary could not assess whether the chart improved SM due to low enrolment.

Several sputum charts are available. We chose the 5-point sputum colour chart, adapted from Bronkotest from among these because it was the only one that has been validated for use in COPD. It had been validated against sputum bacterial load—84% of purulent samples (darker green colour) contained bacteria compared with 38% of mucoid (lighter colour) samples.^9^ It was commercially available and inexpensive (<£2 per patient), practical to use in UK primary care by patients to guide therapy^9^ and patients using the chart to guide antibiotic use rarely experienced treatment failure.^17^ Other colour charts were either less validated or less practical to use. Consistent with our data, a systematic review has shown only moderate specificity and poor sensitivity of colour for bacterial presence.^18^ Multiple studies, though not all before our trial, have shown this. A Dutch study reported a weak association between bacterial load and sputum colour, with no difference in bacterial load between patients with purulent sputum or not. Also, there was no consistent relationship between change in sputum colour and change in bacterial load during admission.^19^ In another study, the mucus score, not necessarily purulence, was the earliest determinant of exacerbation.^20^ Furthermore, eosinophilic sputum in asthma patients may also be purulent.^21^ This body of evidence brings into question whether routine adoption of colour alone, as opposed to a broader picture of symptoms and other features, personalised to the patient, is appropriate within either another trial or routine care.

The sputum substudy showed that PPB presence was not independently associated with purulent sputum, and that coexistence of bronchiectasis was associated with a fourfold increase in risk of purulent sputum, independent of disease state or PPB. Bronchiectasis, a common comorbidity in COPD present in 22% of our participants, is associated with a significant neutrophilic burden,^22^ and our findings may suggest that the Bronkotest is less specific in this cohort of patients. We kept inclusion criteria broad because comorbidity (both respiratory and otherwise) is common in COPD, and we wanted to test if the intervention was useful in all COPD patients, as opposed to specific subgroups. We did not directly confirm bronchiectasis via CT scans, and undiagnosed cases may have been included. Perhaps bronchiectasis may be a better minimisation variable than chronic bronchitis in any future similar work.

Most limitations stem from early trial cessation, leading to less economic data, no economic analysis, limited trial fidelity analyses, no self-efficacy data and severe under-recruitment. CPRD data showed that GOLD C and D patients comprise nearly 46% of registered COPD patients in primary care, indicating that this should not have limited recruitment. We consulted the national COPD audit, which included data from 183 hospitals and 13 414 patients in England and Wales,^23^ and the rates indicated a similar risk to the CPRD data such that we were confident in the veracity of the data with respect to the whole UK. This indicated at trial design stage that we should be able to complete recruitment in 2 years. However, the pandemic impacted both AECOPD rates, and hence eligibility, as well as recruitment preventing the study from reaching its target. Structural barriers to study completion were a major factor; ‘usual care’ was not happening, for example, it took a long time for annual COPD reviews to recommence, which was where our study was designed to sit. Sites also fed back that lack of access to spirometry following the pandemic prevented them from enrolling (when historic tests were not available), that there was a lack of space to see patients (especially during periods of social distancing) and that measures to boost recruitment (text messages, phone calls) were ineffective. Our parallel qualitative study^24^ also concluded that the intervention was unlikely to have significant impact on well-established clinical practices for infection control and patient habits of SM because of issues such as the tension between stewardship of antimicrobials and need to reduce risk of serious illness. Further details on recruitment issues may be found in our report to the funder.^16^ In addition, the study would never have provided guidance to patients unable to expectorate sputum; such patients were advised in our SM plan to take their antibiotic component if there were other features of infection (such as fever) in order to be safe, even though this could also occur with viruses.

## Conclusion

The sputum substudy data and limited effectiveness analyses, while not conclusive, may deprioritise further studies of colour charts, at least in an unselected COPD population.

## Supplementary material

10.1136/bmjresp-2025-003615 online supplemental file 1

10.1136/bmjresp-2025-003615 online supplemental file 2

10.1136/bmjresp-2025-003615 online supplemental file 3

10.1136/bmjresp-2025-003615 online supplemental file 4

## Data Availability

Data are available upon request.
