# Supplementary material for: Sputum colour charts to guide antibiotic self-treatment of acute exacerbation of chronic obstructive pulmonary disease: the Colour-COPD RCT
Source: BMJ Open Respir Res. 2025 Oct 10;12(1):e003615. doi: 10.1136/bmjresp-2025-003615 (PMC12517013; doi:10.1136/bmjresp-2025-003615)
Supplement: online supplemental file 1 [file bmjresp-12-1-s001.pdf]

Multi-centre randomised trial to determine if the use of sputum colour chart is non-inferior to usual care with respect to hospital admissions: The Colour-COPD Trial

Trial Registration: ISRCTN 14955629

Statistical Analysis Plan

SAP Version Number	Protocol Version Number
1.0	5.0

Name of Author:	Eleni Gkini	Role:	Trial Statistician	Affiliation:	BCTU University of Birmingham
Signature of Author:		Date:	06/12/2021		
Name of Chief Investigator:	Dr Alice M Turner	Role:	Chief Investigator	Affiliation:	University of Birmingham
Signature of Chief Investigator:	Alice M Turner	Date:	6th December 2021		
**This Statistical Analysis Plan has been approved by:**					
Name of Approver:	Rajnikant L Mehta	Role:	Senior Statistician	Affiliation:	BCTU University of Birmingham
Signature of Approver:	Rajnikant L Mehta	Date:	7th December 2021		

Statistical Analysis Plan (SAP) Amendments

SAP version number	SAP section number	Description of and reason for change	Timing of change with respect to interim analysis/ final analysis/ database lock	Blind Reviewer	
				Name:	
				Signature:	
				Date:	
				Name:	
				Signature:	
				Date:	
				Name:	
				Signature:	
				Date:	

Abbreviations & Definitions	
**Abbreviation / Acronym**	**Meaning**
AECOPD	Acute exacerbation of COPD
BCTU	Birmingham Clinical Trials Unit
BMI	Body Mass Index
CAT	COPD Assessment Test
CI	Chief Investigator
CONSORT	Consolidated Standards of Reporting Trials
COPD	Chronic Obstructive Pulmonary Disease
CPRD	Clinical Practice Research Database
CRF	Case Report Form
DMC	Data Monitoring Committee
DOB	Date of Birth
EQ-5D-5L	Euroqol 5-Dimension 5-Level
FEV1	Forced Expiratory Volume in 1 second
FVC	Forced Vital Capacity
GEE	Generalised Estimating Equations
GLI	Global Lung Function Index
GP	General Practitioner
HES	Hospital Episode Statistics
HRA	Health Research Authority
HRU	Health Resource Usage
ICF	Informed Consent Form
ICH	International Conference on Harmonisation
ITT	Intention to Treat
ISRCTN	International Standard Randomised Controlled Trial Number
NHS	National Health Service
NICE	National Institute for Health and Care Excellence
PI	Principal Investigator
PIS	Participant Information Sheet
PP	Per-Protocol
QA	Quality Assurance
RCT	Randomised Controlled Trial
REC	Research Ethics Committee
RP	Rescue Pack
RGT	Research Governance Team
SAE	Serious Adverse Event
SAP	Statistical Analysis Plan
SAR	Serious Adverse Reaction

SGRQ	St George's Respiratory Questionnaire
SM	Self-Management
SUSAR	Suspected Unexpected Serious Adverse Reaction
TC	Telephone Call
TSC	Trial Steering Committee
UoB	University of Birmingham
UK	United Kingdom
**Term**	**Definition**
International Standard Randomised Controlled Trial Number	A clinical trial registry
Protocol	Document that details the rationale, objectives, design, methodology and statistical considerations of the study
Randomisation	The process of assigning trial participants to intervention or control groups using an element of chance to determine the assignments in order to reduce bias.
Statistical Analysis Plan	Pre-specified statistical methodology documented for the trial, either in the protocol or in a separate document.

TABLE OF CONTENTS

1. Introduction ... 7
2. Background and rationale ... 7
3. Trial objectives ... 8
4. Trial methods ... 9
4.1. Trial design ... 9
4.2. Trial interventions ... 9
4.3. Primary outcome measure ... 9
4.4. Secondary outcome measures ... 9
4.5. Timing of outcome assessments ... 10
4.6. Randomisation ... 11
4.7. Sample size ... 11
4.8. Framework ... 11
4.9. Interim analyses and stopping guidance ... 12
4.10. Internal Pilot Progression Rules ... 12
4.11. Timing of final analysis ... 13
4.12. Timing of other analyses ... 13
4.13. Trial comparisons ... 13
5. Statistical Principles ... 13
5.1. Confidence intervals and p-values ... 13
5.2. Adjustments for multiplicity ... 14
5.3. Analysis populations ... 14
5.4. Definition of adherence ... 14
5.5. Handing protocol deviations ... 14
5.6. Unblinding ... 15
6. Trial population ... 15
6.1. Recruitment ... 15
6.2. Baseline characteristics ... 15
7. Intervention(s) ... 15
7.1. Description of the intervention(s) ... 15
7.2. Adherence to allocated intervention ... 16
8. Protocol deviations ... 16
9. Analysis methods ... 16
9.1. Covariate adjustment ... 16
9.2. Distributional assumptions and outlying responses ... 16
9.3. Handling missing data ... 17
9.4. Data manipulations ... 17
9.5. Analysis methods – primary outcome(s) ... 19
9.6. Analysis methods – secondary outcomes ... 19
9.7. Analysis methods – exploratory outcomes and analyses ... 21
9.8. Safety data ... 21
9.9. Planned subgroup analyses ... 21
9.10. Sensitivity analyses ... 22
10. Analysis of sub-randomisations ... 23
11. Health economic analysis ... 23
12. Statistical software ... 23
13. References ... 23
Appendix A: Deviations from SAP ... 25
Appendix B: Trial schema ... 26
Appendix C: Schedule of assessments ... 27
Appendix D1: CONSORT flow diagram ... 28

Appendix D2: Baseline characteristics 29
Appendix D3: Description of the intervention(s) 31
Appendix D4: Adherence to allocated intervention 32
Appendix D5: Protocol deviations 32
Appendix D6: Primary outcome results: AECOPD 32
Appendix D7: Secondary outcomes results 33
Appendix D8: Safety 34
Appendix D9: Subgroup and exploratory analysis for primary outcome 35
Appendix D10: Analysis of sub-randomisations – EXACT items 36

1. Introduction

This document is the Statistical Analysis Plan (SAP) for the Colour COPD trial and should be read in conjunction with the current trial protocol. This SAP details the proposed analyses and presentation of the data for the main paper(s) reporting the results for the Colour COPD trial.

The results reported in these papers will follow the strategy set out here. Subsequent analyses of a more exploratory nature will not be bound by this strategy, though they are expected to follow the broad principles laid down here. The principles are not intended to curtail exploratory analysis (e.g. to decide cut-points for categorisation of continuous variables), nor to prohibit accepted practices (e.g. transformation of data prior to analysis), but they are intended to establish rules that will be followed, as closely as possible, when analysing and reporting data.

Any deviations from this SAP will be described and justified in the final report or publication of the trial (using a table as shown in Appendix A: Deviations from SAP). The analysis will be carried out by an appropriately qualified statistician, who should ensure integrity of the data during their data cleaning processes.

2. Background and rationale

The background and rationale for the trial are outlined in detail in the protocol. In brief,

Chronic Obstructive Pulmonary Disease (COPD) is a chronic condition affecting 2 million people in the UK, causing over 140,000 hospital admissions and 1.7% of UK hospital bed days per year (1). Common day to day symptoms include breathlessness, which is typically worse on exertion, and cough productive of sputum. COPD is defined by airflow obstruction on spirometry, this being a ratio less than 0.7 and lower than the lower limit of normal for age in the forced expiratory volume in 1 second (FEV1) and forced vital capacity (FVC) after administration of a bronchodilator.

This study addresses the problem of personalising and thus optimising effectiveness of Acute exacerbation of COPD (AECOPD) management. It is now widely accepted that each year around half of all patients with COPD have frequent AECOPD (≥2 per year (2)), and this is clinically relevant as these patients have poorer prognosis.

Evaluation of approaches to personalising treatment for COPD is a current research priority, as disease heterogeneity is increasingly recognized. AECOPD are an important part of the personalization agenda, given their frequency in the COPD population and the burden that accrues from them to our health economy. In addition they are a particularly important group for study since hospitalization rates are rising (3).

3. Trial objectives

The primary objective is to assess whether use of the 5 point sputum colour chart, alongside a Self-Management (SM) plan and rescue pack (RP) containing 5 days of antibiotic and steroid treatment (the intervention) is safe, as defined by being not substantially worse compared to use of the plan and pack alone (best usual care) for patient hospitalisation admission for AECOPD at 12 months post enrolment (defined by randomisation time point).

Secondary objectives are as follows:

1. Assess whether the intervention is safe, as defined by the rate of 30 and 90 day AECOPD readmissions, rate of treatment failure (defined by ongoing symptoms and/or requirement for treatment in the 14 days after a self-managed event), and time to next AECOPD after a self-managed event

2. Determine whether use of the intervention is effective at 12 months after enrolment in terms of reducing self-reported antibiotic use when compared to best usual care, including rescue pack, as well as reducing adverse events related to antibiotics (e.g. oral thrush).

3. Describe the effect of the intervention on number of unscheduled General Practitioner (GP) attendances, for AECOPD, in the 12 months post enrolment.

4. Describe the effect of the intervention on unreported AECOPD rate through a sub-study using daily symptom diaries. Unreported AECOPD are defined by daily symptom change in the absence of input from a healthcare professional, or reporting symptom change to a healthcare professional. This sub-study will allow us to determine if unreported AECOPD are impacted upon by the intervention, and their rate in UK primary care; it also allows us to assess adherence to the intervention's advice accurately.

5. Describe the effect of the intervention on antibiotic resistance patterns in sputum of people with COPD. This sub-study also allows us to assess the appropriateness of antibiotic use by objectively confirming sputum colour at AECOPD, and confirming presence of bacteria.

6. To assess fidelity of delivery of the intervention by use of a checklist inquiring on critical features of education around colour chart and self-management plan use.

7. To assess adherence to SM plan advice by comparing use of AECOPD treatment to daily symptoms (e-diary subgroup only).

8. To explore social acceptability and practical responses to the intervention by interviewing both staff delivering the intervention and participants receiving the intervention.

Economic objectives are as follows:

1. Determine the cost-effectiveness and cost-utility of using a colour chart as part of a SM plan.

4. Trial methods

4.1. Trial design

Colour COPD is a prospective 2 arm, multi-centre, open label, and parallel-group non-inferiority randomised controlled trial investigating the use of Sputum Colour Charts to guide antibiotic self-treatment of acute exacerbation of COPD in patients with COPD. There is an integral pilot, sub-studies of acceptability of the intervention, antibiotic resistance patterns in sputum, daily symptom control and an economic analysis. The study is set in primary care, recruiting from approximately 80 GP practices predominantly within Birmingham and Greater Manchester areas. See Appendix B: Trial schema for trial schema.

Due to the nature of this intervention it is not possible to blind at the patient or investigator level. The research team member making the phone calls to the participants will be blinded to the participant's allocation.

There is a 9 month internal pilot among 50% of practices and 15% of participants (n=444). For further details please refer to section 4.10 and section 2.1 within the protocol.

4.2. Trial interventions

Experimental Arm: use of the 5 point sputum colour chart, adapted from Bronkotest® a SM plan and RP containing 5 days of antibiotic and steroid treatment.

Control Arm: use of the plan and pack alone (best usual care).

4.3. Primary outcome measure

A binary outcome assessing incidence of at least one AECOPD over 12 months post randomisation where patients needed hospitalisation (defined by hospital discharge letter/coding). Data will be obtained from the medical record National Health Service (NHS) digital (hospital episode statistics; HES) which ensures any admissions not reported to the GP are picked up.

4.4. Secondary outcome measures

The secondary outcomes are as follows :

- Self-reported AECOPD (including those for which admission is required) obtained by telephone calls to patients every 3 months (at 3, 6, 9 and 12 months post randomisation respectively);
- Self-reported antibiotic and steroid prescriptions for AECOPD at 12 months post randomisation;
- All cause hospital admission, from HES and/or participant self-report at 12 months post randomisation;
- Readmissions to hospital for AECOPD at 30 and 90 days, from HES and participant self-report at 12 months post randomisation;
- Bed days due to AECOPD at 12 months post randomisation;
- Mortality , as determined by the medical record at 12 months post randomisation;
- Self-reported unscheduled GP visits, for AECOPD at 12 months post randomisation;
- Self-reported prescriptions for 2nd courses of antibiotics within 14 days of self-reported event (defined as treatment failure) at 12 months post randomisation;
- Self-reported prescriptions for anti-fungals (e.g. for oral thrush) at 12 months post randomisation;
- Quality of life (COPD assessment test [CAT], Euroqol 5-Dimension 5-Level [EQ-5D-5L]) at 3 monthly intervals (at 3, 6, 9 and 12 months post randomisation respectively):
    - The CAT score can range from 0 to 40 and the total score will be used to compare between groups. There are no subscales within it
    - The EQ-5D-5L generates a 5-digit score from 11111, which indicates no problems on any of the five dimensions to 55555, which indicates extreme problems on all of the five dimensions, the total score will be used for the economic evaluation only and;
- Antibiotic resistance (determined by sputum culture at baseline, all AECOPD and 12 months);
- Health Resource Usage (HRU); self-reported by participant every 3 months, and submitted using a specific HRU Case Report Form (CRF) (at 3, 6, 9 and 12 months post randomisation respectively).

All secondary outcomes measured at 12 months after enrolment unless otherwise stated. Comparisons will occur for the 12 month time point only, unless otherwise stated, and where data is collected more frequently the summed data at 12 months will be used for between arm comparisons.

The EQ-5D-5L total score and the Health Resource Usage (HRU) outcomes will be assessed in the economic evaluation analysis and a separate document will detail the analysis plan for the health economic outcomes.

4.5. Timing of outcome assessments

The schedule of trial procedures and outcome assessments are given in Appendix C: **Schedule of assessments**.

4.6. Randomisation

Participants will be randomised by computer (or telephone if practices have poor online access) at the level of the individual in a 1:1 ratio to either 5 point sputum colour chart, adapted from Bronkotest® colour chart or usual care as described previously, and this will be conducted by the Birmingham Clinical Trials Unit (BCTU) team.

A minimisation algorithm will be used within the online randomisation system to ensure balance in the treatment allocation over the following variables, which centre on factors influencing AECOPD and admission:

- Severity of COPD (see Figure 1 in protocol section 1.1)
  - C: CAT<10, 2 or more exacerbations in the last 12 months OR ≥1 hospital admission for an exacerbation
  - D: CAT≥10, 2 or more exacerbations in the last 12 months OR ≥1 hospital admission for an exacerbation
- Presence or absence of chronic bronchitis
- Prior COPD hospitalization (yes or no within the 12 months prior to enrolment)
- Age, as defined by <65 years, 65-80 years (inclusive), >80 years

In addition, GP practice will be included to adjust for any stratification effects.

A 'random element' will be included in the minimisation algorithm, so that each participant has a probability (unspecified here), of being randomised to the opposite treatment that they would have otherwise received. Full details of the randomisation specification will be stored in a confidential document at BCTU.

4.7. Sample size

We have used hospitalisation rates from the clinical practice research database (CPRD) to determine event rate for our primary outcome (4). Assuming a 1-sided significance level of 2.5% and a rate of admission in each group of 65% of that in the referenced data, with a non-inferiority margin of 6 percentage points, we would need to enrol 1329 patients in each of the intervention and control groups (2658 in total) to have 90% power for determining whether the results in the usual care group were non-inferior to those in the intervention group. Assuming dropout/lost to follow-up/non-adherence rate of 10% we therefore need to recruit 2954 patients.

4.8. Framework

The objective of the trial is to test the non-inferiority of one intervention to another.

The primary outcome is rate of hospital admission for AECOPD. Assuming a 1-sided significance level of 2.5% and a true success rate in each group of 65%, with a non-inferiority

margin of 6 percentage points, the study has 90% power to detect non-inferiority of the intervention.

4.9. Interim analyses and stopping guidance

A separate Data Monitoring Committee (DMC) reporting template will be drafted and agreed by the DMC including an agreement on which outcomes will be reported at interim analyses. The statistical methods stated in this SAP will be followed for the outcomes included in the DMC report, where possible.

Interim analyses of safety and efficacy for presentation to the independent DMC will occur as per standard trials unit processes. Criteria for stopping or modifying the study based on this information will be ratified by the DMC.

4.10. Internal Pilot Progression Rules

There is a 9 month internal pilot among 50% of practices and 15% of participants (n=443). Data from these elements form our stop/go criteria.

Accrual of 222 participants per group (Total = 444) in 9 months
At least 40 sites open to accrual at 9 months
At least 50% of sites open to accrual successfully recruiting a participant at 9 months

The internal pilot has three purposes:

(i) To assess recruitment rate and exclusions. We aim for 50% of sites enrolling at 9 months from study approval by the relevant authorities, and target an overall recruitment rate of 6 patients per site per month. This target is an average across all sites, not per site and informs progression.

(ii) To assess spirometry in the GP record, specifically the rate of recording of raw values (which are required alongside age and sex to calculate predicted values), its ease of remote electronic extraction and any inconsistency between coded diagnosis of COPD and spirometry values indicative of airflow obstruction.

(iii) To refine sample size calculation, and determine practicality of HES data for this. We will do this by reviewing incidence of hospitalisations for AECOPD and AECOPD rate (including unreported events in the e-diary study) to determine if sample size remains accurate.

Progression criteria based on recruitment and data received

Progression criteria: We have taken into account guidance from a Medical Research Council Hubs for Trials Methodology Research workshop when determining stop/go criteria, and will report our pilot results according to their suggestions (5). Areas considered by the workshop as suitable progression criteria included recruitment rate, protocol adherence and outcome rate.

A traffic light system of green (go), amber (amend) and red (stop) is being used and summarised in the table below. If a red or amber criterion is hit the trial steering committee will be consulted. The expectation is that sites would be assisted to address amber criteria and red might trigger the trial to stop, depending on steering committee views.

Criteria	Green	Amber	Red
**Recruitment rate (average per site)**	≥5 per month	3-4 per month	<3 per month
**% excluded due to perceived unsuitability for self-management**	<30%	30-59%	≥60%
**Completeness of critical data**	>90%	80-90%	<80%

4.11. Timing of final analysis

The final analysis for the trial will occur after all participants have completed the 1 year assessment and the corresponding outcome data has been entered onto the trial database and validated as being ready for analysis. This is provided that the trial has not been stopped early for any reason (e.g. DMC advice or funding body request). E-diary and sputum sub-studies are also analysed at completion of the study. This analysis will include data items up to and including the 1 year assessment and no further.

4.12. Timing of other analyses

Not applicable.

4.13. Trial comparisons

All references in this document to 'group' refer to: 5 point sputum colour chart, adapted from Bronkotest®, alongside a standardised SM plan and rescue pack of 5 days antibiotic and steroid treatment or SM plan and rescue pack alone (best usual care).

5. Statistical Principles

5.1. Confidence intervals and p-values

All estimates of differences between groups will be presented with one-sided 95% confidence intervals, unless otherwise stated. Statistical significance (p-values<0.05) will confirm non-inferiority.

5.2. Adjustments for multiplicity

No correction for multiple testing will be made.

5.3. Analysis populations

All primary analyses (primary and secondary outcomes including safety outcomes) will be by intention-to-treat (ITT). Participants will be analysed in the intervention group to which they were randomised, and all participants shall be included whether or not they received the allocated intervention. This is to avoid any potential bias in the analysis.

For this non-inferiority trial, 'Per-Protocol' (PP) analysis will also be conducted for the primary outcome excluding patients with major violations. Refer to section 9.10 for further details on any sensitivity analyses.

5.4. Definition of adherence

We define adherence as how well patients adhere to advice in their SM plan in the e-diary sub study where daily symptoms are collected. Specifically we will examine the frequency of unreported AECOPD, i.e. events where symptoms occur but patients do not report an AECOPD and do not take treatment for it. We will also use the self-reported AECOPD data compared to the medically confirmed AECOPD data in the whole trial population to infer this; if self-reported events are greater than confirmed events this implies events for which treatment is not taken, and thus potentially poor adherence to the SM plan.

5.5. Handing protocol deviations

A protocol deviation is defined as a failure to adhere to the protocol such as errors in applying the inclusion/exclusion criteria, the incorrect intervention being given, incorrect data being collected or measured, follow-up visits outside the visit window or missed follow-up visits. We will apply a strict definition of the ITT principle and will include all participants as per the ITT population described in section 5.3 in the analysis, in some form, regardless of deviation from the protocol (6). This does not include those participants who have specifically withdrawn consent for the use of their data in the first instance; however, these outcomes will be explored as per other missing responses.

Telephone calls (TC) 1, 2, 3, 4 all have schedule windows of ± four days and four weeks respectively. Two week follow-up questionnaires will be considered valid provided they have been completed ± four days from Telephone call 1 (TC1). Three month follow-up questionnaires will be considered valid provided they have been completed ± four weeks from Telephone call 2 (TC2). Six month follow-up questionnaires will be considered valid provided they have been completed ± four weeks from Telephone 3 (TC3). Nine month follow-up

questionnaires will be considered valid provided they have been completed ± four weeks from Telephone 4 (TC4). Twelve month follow-up questionnaires will be considered valid provided they have been completed ± two months.

5.6. Unblinding

Not applicable, Colour-COPD is an open-label study.

6. Trial population

6.1. Recruitment

A flow diagram (as recommended by CONSORT (7)) will be produced to describe the participant flow through each stage of the trial. This will include information on the number (with reasons) of losses to follow-up (drop-outs and withdrawals) over the course of the trial. A template for reporting this is given in Appendix D1**: CONSORT flow diagram**.

6.2. Baseline characteristics

The trial population will be tabulated as per Appendix D2**: Baseline characteristics**. Categorical data will be summarised by number of participants, counts and percentages. Continuous data will be summarised by the number of participants, mean and standard deviation if deemed to be normally distributed or number of participants, median and interquartile range if data are skewed, and ranges if appropriate. Tests of statistical significance will not be undertaken, nor confidence intervals presented (8).

7. Intervention(s)

7.1. Description of the intervention(s)

The intervention is the 5 point sputum colour chart, adapted from Bronkotest® 5 colour sputum chart, issued alongside a standardised SM plan and rescue pack at the time of randomisation only. Instructions on how to use the intervention are available from the manufacturer and are shown briefly in the patient self-management plan, which is standardised. A template for reporting information on the intervention(s) is given in Appendix D3**: Description of the intervention(s)**.

7.2. Adherence to allocated intervention

A cross-tabulation of allocated intervention by the adherence categories stated in section 5.4 will be produced (proportions and percentages). A template for reporting adherence is given in Appendix D4: **Adherence to allocated intervention**.

8. Protocol deviations

Frequencies and percentages by group will be tabulated for the protocol deviations as per Appendix D5: **Protocol deviations**.

9. Analysis methods

Intervention groups will be compared using mixed effects log binomial regression model adjusting for all covariates as specified in section 9.1, where possible.

9.1. Covariate adjustment

In the first instance, intervention effects between groups for all outcomes will be adjusted for the minimisation parameters listed in section 4.6. Categorised continuous variables (e.g. age) will be treated as continuous variables in this adjustment. All minimisation variables will be treated as fixed effects, apart from GP practice which will be included as a random effect.

Other covariate adjustment will be baseline values for parameters where available (e.g. an analysis of questionnaire scores at 12 months will also include the baseline score as a covariate in the model). Furthermore, methods of delivery used to collect the research data which is a categorical variable (Face to face, Video consultation, Telephone with Video and Telephone only) will be factored into the analysis.

If the log-binomial model fails to converge, we first of all will omit the GP practice random effect and re-analyse the data. If this model fails to converge a Poisson regression model with robust standard errors will be used to estimate the same parameters (9). If this also fails to converge, unadjusted estimates will be produced from the log-binomial model. It will be made clear in the final report why this occurred (e.g. not possible due to low event rate/lack of model convergence).

9.2. Distributional assumptions and outlying responses

Distributional assumptions (e.g. normality of data and/or regression residuals for continuous outcomes) will be assessed visually prior to reporting the results of the analysis. Although in the first instance the proposed primary method of estimation in this analysis plan will be

followed, if distributional assumptions are considered to be violated, the impact of this will be examined through sensitivity analysis; this will consist of transformation of responses prior to analysis (e.g. log transformation) in the first instance. If extreme values are apparent and considered to be affecting the integrity of the analysis, a sensitivity analysis consisting of removing the outlying response(s) and repeating the analysis will be performed. Output from these analyses, if performed, will be described and presented alongside the original analysis (or included, e.g. in appendices) with the excluded values clearly labelled. See section 9.10 for further details regarding sensitivity analyses.

9.3. Handling missing data

In the first instance, analysis will be completed on received data only with every effort made to follow-up participants to minimise any potential for bias. To examine the possible impact of missing data on the results, and to make sure we are complying with the intention-to-treat principle, sensitivity analysis will be performed on the primary outcome measure (10). See section 9.10 for further details regarding sensitivity analyses.

9.4. Data manipulations

The Trial Statistician will derive all responses from the raw data recorded in the database as follows:

1. COPD Assessment Test (CAT)

The CAT response scale are coded as follows:

- Question 1: 0-I never cough=0, 1=1, 2=2, 3=3, 4=4, 5=I cough all the time
- Question 2: 0-I have no phlegm on my chest at all=0, 1=1, 2=2, 3=3, 4=4, 5=My chest is full of phlegm
- Question 3: 0-My chest does not feel right at all=0, 1=1, 2=2, 3=3, 4=4, 5=My chest feels very tight
- Question 4: 0-When I walk up a hill or a flight of stairs I am not out of breath=0, 1=1, 2=2, 3=3, 4=4, 5=When I walk up a hill or a flight of stairs I am completely out of breath
- Question 5: 0-I am not limited to doing any activity at home =0, 1=1, 2=2, 3=3, 4=4, 5=I am completely limited to doing all activities at home

- Question 6: 0-I am confident leaving my home despite my lung condition=0, 1=1, 2=2, 3=3, 4=4, 5=I am not confident leaving my home at all because of my lung condition

- Question 7: 0-I sleep soundly=0, 1=1, 2=2, 3=3, 4=4, 5=I do not sleep soundly because of my lung condition

- Question 8: 0-I have lots of energy=0, 1=1, 2=2, 3=3, 4=4, 5=I have no energy at all

The CAT total score will be derived by taking the sum of these items.

Furthermore we will also consider categorising the CAT total score as follows:

CAT score	Impact level	Broad clinical picture of the impact of COPD by CAT score	Possible management considerations
>30<br><br><br><br><br><br>>20	Very high<br><br><br><br><br><br>High	Their condition stops them doing everything they want to do and they never have any good days. If they can manage to take a bath or shower, it takes them a long time. They cannot go out of the house for shopping or recreation, or do their housework. Often, they cannot go far from their bed or chair. They feel as if they have become an invalid.<br><br>COPD stops them doing most things that they want to do. They are breathless walking around the home and when getting washed or dressed. They may be breathless when they talk. Their cough makes them tired and their chest symptoms disturb their sleep on most nights. They feel that exercise is not safe for them and everything they do seems too much effort. They are afraid and panic and do not feel in control of their chest problem.	Patient has significant room for improvement<br>In addition to the guidance for patients with low and medium impact CAT scores consider:<br>• Referral to specialist care (if you are a primary care physician)<br><br>Also consider:<br>• Additional pharmacological treatments<br>• Referral for pulmonary rehabilitation<br>• Ensuring best approaches to minimising and managing exacerbations
10-20	Medium	COPD is one of the most important problems that they have. They have a few good days a week, but cough up sputum on most days and have one or two exacerbations a year. They are breathless on most days and usually wake up with chest tightness or wheeze. They get breathless on bending over and can only walk up a flight of stairs slowly. They either do their housework slowly or have to stop for rests.	Patient has room for improvement – optimise management<br>In addition to the guidance provided for patients with low impact CAT scores consider:<br>• Reviewing maintenance therapy – is it optimal?<br>• Referral for pulmonary rehabilitation<br>• Ensuring best approaches to minimising and managing exacerbations<br>• Reviewing aggravating factors – is the patient still smoking?
<10	Low	Most days are good, but COPD causes a few problems and stops people doing one or two things that they would like to do. They usually cough several days a week and get breathless when playing sports and games and when carrying heavy loads. They have to slow down or stop when walking up hills or if they hurry when walking on level ground. They get exhausted easily.	• Smoking cessation<br>• Annual influenza vaccination<br>• Reduce exposure to exacerbation risk factors<br>• Therapy as warranted by further clinical assessment.
5		Upper limit of normal in healthy non-smokers	

Primary outcome: Patient hospitalised due to AECOPD over 12 months follow-up period after enrolment.

Other outcomes:

- Age at randomisation = (Randomisation date - date of birth (DOB)) / 365.25
- Body Mass Index (BMI) = Weight (kg) / [Height (m)]^2

9.5. Analysis methods – primary outcome(s)

A template for reporting the primary outcome is given in Appendix D6**: Primary outcome results:** .

For the analysis of the primary outcome measure, a mixed-effects log-binomial model will be used to generate relative risks along with 95% confidence intervals, adjusting for the minimisation parameters (see section 9.1). The Risk Difference will be presented using the identity link. Statistical significance of the treatment group parameter will be determined (p-value generated) through examination of the associated chi-squared statistic. If covariate adjustment fails, we first of all will omit GP practice from our model and re-analyse the data. Estimates will be produced with a clear explanation in the output why this occurred, e.g. due to a low event rate i.e. sparse or lack of model convergence. If the revised log-binomial model fails to converge a Poisson regression model (9) with robust standard errors will be used to estimate the same parameters. If this also fails to converge, unadjusted estimates will be produced from the log-binomial model. It will be made clear in the final report why this occurred.

See section 9.1 for covariate adjustment and model convergence.

9.6. Analysis methods – secondary outcomes

A template for reporting the secondary outcomes is given in Appendix D7**: Secondary outcomes results**.

- *Self-reported AECOPD*

This information will be collected from the participant via telephone call made by the research team at 3, 6, 9 and 12 months respectively. The evaluation self-reported AECOPD will be analysed using repeated measures mixed effects techniques for binary outcome adjusting for the intervention group, baseline recordings (if available), minimisation variables and associated relative risks will be calculated and associated 95% confidence intervals.

- *Self-reported antibiotic and steroid prescriptions for AECOPD*

The assessment of antibiotic and steroid prescription for AECOPD will be summarised using descriptive statistics by intervention group. Furthermore, this recorded information will be analysed using a mixed effects log binomial regression model with the presentation of calculate relative risk and associated 95% confidence intervals. The Risk Difference will be presented using the identity link. Also adjusted analysis will be conducted accounting for baseline recordings (if available) and minimisation variables.

- *All cause hospital admission/ Readmission to hospital for AECOPD (30 days, 90 days)/Self-reported unscheduled GP visits for AECOPD*

The hospital admissions frequency count will be summarised by treatment group. Poisson regression techniques will be used to calculate relative risks. Furthermore adjusted analysis will be conducted accounting for baseline recordings (if available) and minimisation variables. Likewise, the same methods will also be applied for Unscheduled GP visits. Also, statistical techniques for 'Mortality' (below) will also be used for All cause hospital admissions.

- *Bed days due to AECOPD*

Total number of bed days due to AECOPD will be summarised using basic descriptive statistics (Mean(SD) or medians(IQR) if data is skewed). We will also use linear regression models to estimate differences between the two treatment groups adjusting for baseline recordings (if available) and minimisation variables.

- *Mortality, as determined by the medical record*

The mortality within each treatment group will be analysed using survival analysis techniques: Kaplan Meier curves with log rank test. Furthermore, Cox regression methods will be applied to investigate treatment group differences adjusting for baseline recordings (if available) and minimisation variables. The time to mortality will be measured at 12 months post randomisation, in which the date of censoring will be the date of either withdrawal-and patient wishes to withdraw completely i.e. no further data will be collected, loss-to-follow-up, or the exact occurs 12 months from the date of randomisation, whichever occurs first. The difference between this date of censoring and the date of randomisation will be time to censoring. If a participant has been withdrawn, or lost-to-follow-up prior to 12 months from the date of randomisation, they will be classed as a censored observation.

- *Self-reported prescriptions for 2nd courses of antibiotics within 14 days of self-reported event (defined as treatment failure)*

The evaluation of second course of antibiotics within 14 days will be analysed in a similar manner to Self-reported antibiotic and steroid prescriptions for AECOPD.

- *Self-reported prescriptions for anti-fungals (e.g. for oral thrush)*

Prescription for an anti-fungal will be summarised for each intervention group using descriptive statistics and will be analysed in a similar manner to Self-reported antibiotic and steroid prescriptions for AECOPD.

- *QoL (COPD assessment test(CAT)) at 3 monthly intervals (at 3, 6, 9, and 12 months post randomisation respectively)*

Recorded patient questionnaires (CAT) via telephone calls will be converted into scores and treatment groups analysed using mixed effects repeated measures (multi-level) model adjusting for baseline recordings (if available) and minimisation variables.

- *Antibiotic resistance (from sputum culture at Baseline, First AECOPD and 12 months)*

Data from this variable will be analysed in a similar manner to Self-reported AECOPD.

- *Health Resource Usage (HRU); self-reported by participant every 3 months, and submitted using a specific HRU CRF*

Flow-volume data recorded for calculated: FEV_1, FVC, FEV_1/FVC ratio; the Global Lung Function Index 2012 (GLI-2012) equations freely *available* software will be used to analyse this data. Analysis will be performed using GAMLSS package in R.

9.7. Analysis methods – exploratory outcomes and analyses

Any data that does not form a pre-specified outcome will be presented using simple summary statistics by intervention group (i.e. numbers and percentages for binary data and means (or medians) and standard deviations (or inter-quartile ranges) for continuous normal (or non-normal) data.

Education level (No formal education / GCSE, CSE, O level or equivalent / A-level/AS level or equivalent / Degree level or higher / Other) will be taken into consideration and to assess interaction with the intervention.

9.8. Safety data

The number and percentage of participants experiencing any adverse events, serious adverse events (SAEs) and suspected unexpected serious adverse reactions (SUSARs) will be presented by intervention group. Statistical significance will be determined by chi-squared test. The total number of SAEs in each group will also be given along with a descriptive table of the events. A template for reporting this safety data is given in Appendix D8: **Safety**.

9.9. Planned subgroup analyses

Interpretation of subgroup analysis will be treated with caution (output will be treated as exploratory rather than definitive (11, 1212)). Analysis will be limited to the primary outcome which is the hospital admission rate at 12 months (where the primary reason for admission is AECOPD only), and the following subgroups:

- Severity of COPD (C: CAT<10, 2 or more exacerbations in the last 12 months OR ≥1 hospital admission for an exacerbation / D: CAT≥10, 2 or more exacerbations in the last 12 months OR ≥1 hospital admission for an exacerbation)

- Presence of chronic bronchitis (Presence / Absence)
- Prior COPD hospitalization (Yes / No within the 12 months prior to enrolment)
- Age, as defined by (<65 / 65-80 (inclusive) / >80)
- Patients with co-morbid bronchiectasis vs those without
- Patients with asthma vs those without
- Patients with chronic bronchitis vs those without
- Patients hospitalized in the year prior to inclusion vs those who were not hospitalized
- GOLD groups C-D
- Describe our AECOPD results with respect to blood eosinophils and classes of COPD treatment used during the study (specifically LAMA/LABA/ICS/roflumilast/carbocisteine). In the rare scenario that two or more COPD treatments have been administered a suitable combination of these treatments will be presented in order for these groups to be independent.
- Delivery methods of appointment 1 (screening) (Face to face / Video consultation / Telephone with video links / Telephone only).
- Delivery methods of appointment 2 (12 months follow up) (Face to face / Video consultation / Telephone with video links / Telephone only).

The effects of these subgroups will be examined by adding the subgroup by treatment group interaction parameters to the log-binomial model. Statistical significance (p-values) of these interaction parameters will be determined by Wald tests. Differences between the treatment groups within subgroups will only be examined if the interaction parameter is shown to statistically important (p-value <0.05). A template for reporting the subgroup analyses for the primary outcome is given in Appendix D9: **Subgroup and exploratory analysis for primary outcome**.

9.10. Sensitivity analyses

Sensitivity analyses will be limited to the primary outcome and will consist of:

- Per-protocol analysis (population described in sections 5.3 and 5.4);
- We would also conduct a sensitivity analysis to assess:
    - The generalisability of the results by comparing baseline characteristics of those who provide primary outcome data to those who do not provide primary outcome data.
    - The effect of missing responses. This sensitivity analysis will consist of simulating the missing responses using a multiple imputation approach (9). A Markov chain Monte Carlo method (MCMC) that assumes an arbitrary missing data pattern and a multivariate normal distribution will be used. Variables including treatment group and the minimisation variables listed in section 7 (except centre) will be included in the model and used to generate 20 simulated data-sets. The analysis will then be performed (as described below) on each set with the results combined using Rubin’s rules to obtain a single set of results (treatment effect estimate and confidence interval).

10. Analysis of sub-randomisations

E-diary users (n=300 (10% of the total population)) enrolled consecutively from the start.

Analysis of e-diary AECOPD using the EXACT score will be conducted by a clinical lecturer. 'E-diary analysis will focus on diary defined AECOPD, both reported and unreported, and will use similar principles to those described for self-reported AECOPD in the secondary outcomes. Diary defined AECOPD will be split into reported and unreported according to methods described in prior trials (13).

11. Health economic analysis

As indicated in the protocol there will also be an economic analysis. The details of this analysis are documented separately.

12. Statistical software

Statistical analysis will be undertaken in the following statistical software packages: SAS (version 9.4) or STATA (version 12 or higher).

13. References

1. The battle for breath - the impact of lung disease in the UK. London: British Lung Foundation, 2016.

2. Hurst JR, Vestbo J, Anzueto A, Locantore N, Mullerova H, Tal-Singer R, et al. Susceptibility to exacerbation in chronic obstructive pulmonary disease. N Engl J Med. 2010;363(12):1128-38.

3. Stone RA, Holzhauer BJ, Lowe D, Searle L, Skipper E, Welham S, et al. National COPD audit programme. COPD: Who cares matters. London, UK: Royal College of Physicians, 2015 Contract No.: eISBN 978-1-86016-559-7.

4. Merinopoulou E, Raluy-Callado M, Ramagopalan S, MacLachlan S, Khalid JM. COPD exacerbations by disease severity in England. Int J Chron Obstruct Pulmon Dis. 2016;11:697-709.

5. Avery KN, et al. Informing efficient randomised controlled trials: exploration of challenges in developing progression criteria for internal pilot studies. BMJ open. 2017;7:e013537.

6. Gupta SK. Intention-to-treat concept: A review. Perspect Clin Res. 2011;2(3):109-112.

7. Piaggio G, Elbourne DR, Pocock SJ, Evens SJW, Altman DG. Reporting of Noninferiority and Equivalence Randomized Trials. JAMA 2012;V(308), No.24.

8. Altman DG, Dore CJ. Randomisation and baseline comparisons in clinical trials. Lancet 1990; 335:149-53.

9. Zou G. A modified Poisson regression approach to prospective studies with binary data. Am J Epidemiol. 2004;159(7):702-6.

10. White IR, Horton NJ, Carpenter J, Pocock SJ. Strategy for intention to treat analysis in randomised trials with missing outcome data. BMJ. 2011;342:d40.

11. Guideline on the investigation of subgroups in confirmatory clinical trials: EMA January 2019.

12. Wand R, Lagakos SW, Ware JH, Hunter DJ, Drazen JM. Reporting of subgroups analyses in clinical trials. NEJM. 2007;357:2189-94.

13. Paul W. Jones, Rosa Lamarca, Ferran Chuecos, Dave Singh, Alvar Agustí, Eric D. Bateman, Gonzalo de Miquel, Cynthia Caracta, Esther Garcia Gil. European Respiratory Journal 2014 44: 1156-1165.

Appendix A: Deviations from SAP

This report below follows the statistical analysis plan dated *<insert effective date of latest SAP>* apart from following:

Section of report not following SAP	Reason
<insert section >	<insert, e.g. exploratory analyses request by TMG>

Appendix B: Trial schema

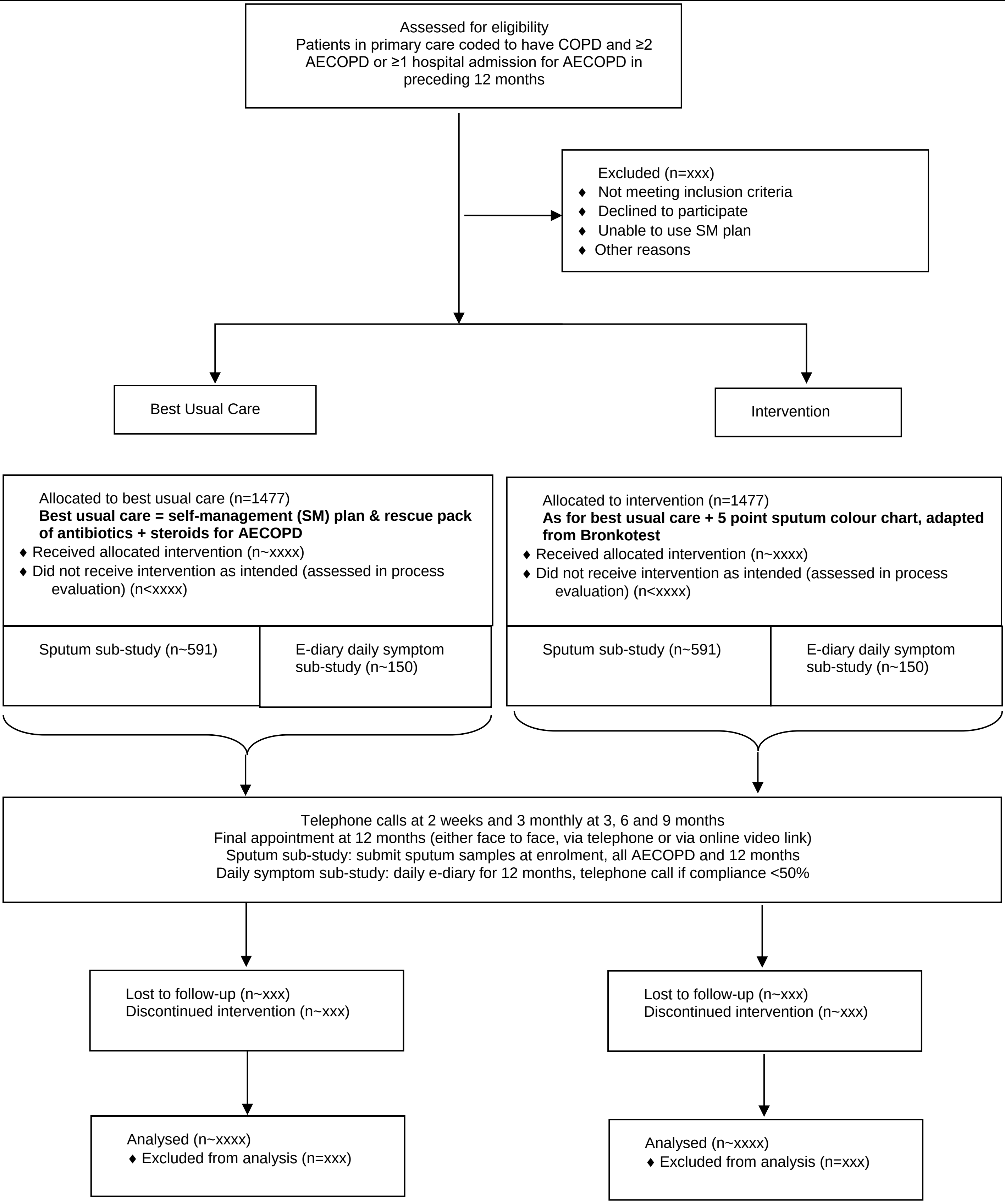

Appendix C: Schedule of assessments

Appointment	Pre-screening	Screening Appointment 1	Telephone Call TC1 *(E-diary sub-study and fidelity checks)*	Telephone Call TC2	Telephone Call TC3	Telephone Call TC4	End of Study Appointment 2
**Weeks**		**Day 0**	**2 weeks**	**3 months**	**6 months**	**9 months**	**12 months**
**Time window for appointment**			**+/- 7 days**	**+/- 4 weeks**	**+/- 4 weeks**	**+/- 4 weeks**	**+/- 2 month**
Review of Inclusion & Exclusion criteria	X	X					
Informed consent		X					
Randomisation		X					
Intervention		X					
Demographics		X					
Medical history		X					
Smoking status		X					X
Concomitant medication		X					X
Educational level		X					
Chronic bronchitis		X					
FEV1 & FVC		X					X
MRC score		X					X
Trial fidelity			X				
Adverse events				X	X	X	X
CAT score		X		X	X	X	X
EQ-5D-5L		X		X	X	X	X
AECOPD rate		X		X	X	X	X
Hospitalisation review		X					X

Appendix D1: CONSORT flow diagram

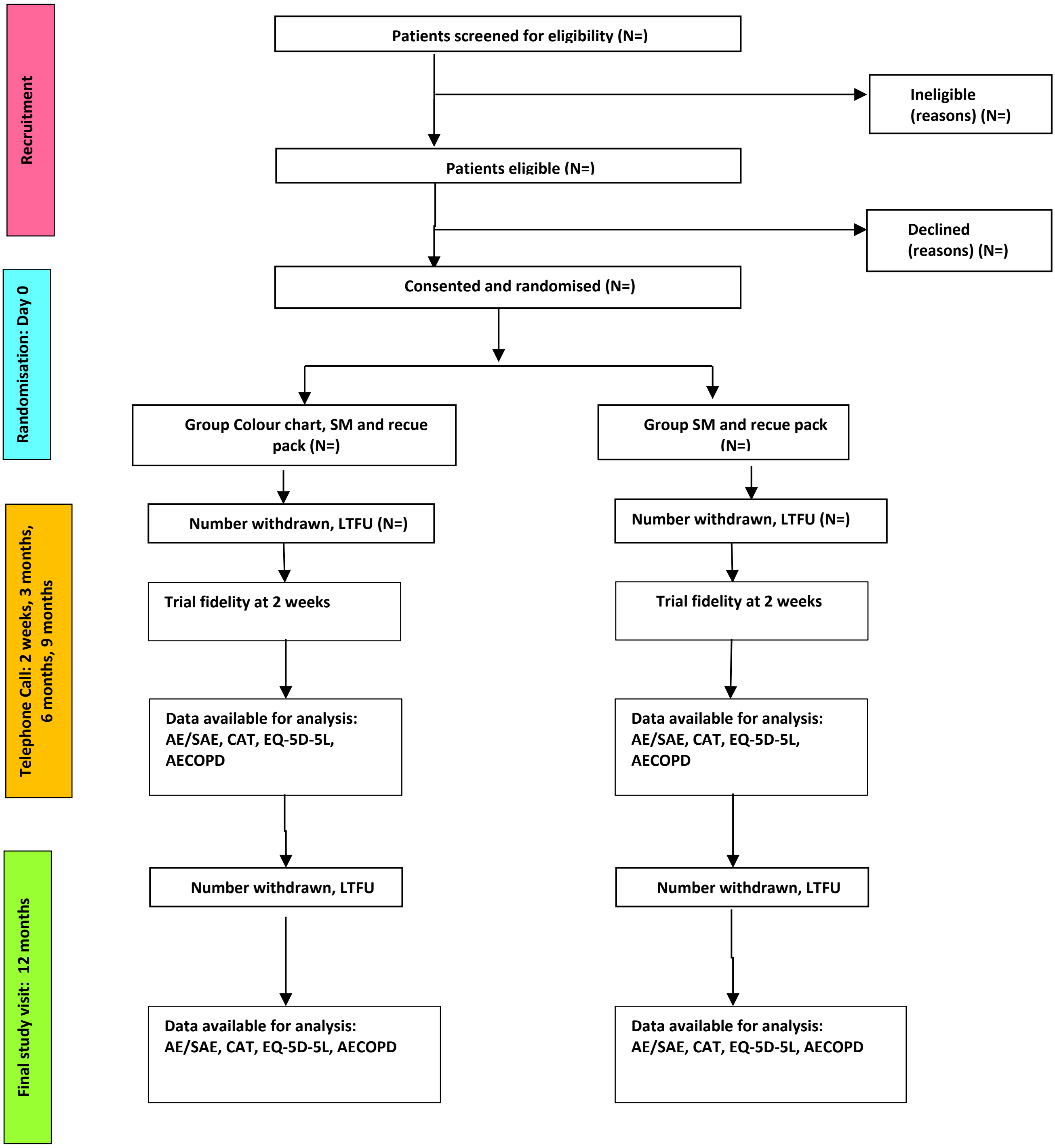

Key:
SM: Self Management
LTFU: Lost to Follow-Up
CAT: COPD assessment test; AE/SAE: Adverse event/Serious adverse event
AECOPD: Acute exacerbation of COPD; EQ-5D-5L

Appendix D2: Baseline characteristics

		Colour chart, SM and recue pack (N=xxx)	SM and recue pack (N=xxx)	Overall (N=xxx)
**Minimisation variables**				
Severity of COPD	Category 1	n (%)	n (%)	n (%)
	Category 2	n (%)	n (%)	n (%)
	…	…	…	…
Presence of chronic bronchitis	Category 1	n (%)	n (%)	n (%)
	Category 2	n (%)	n (%)	n (%)
	…	…	…	…
Prior COPD hospitalization	Category 1	…	…	…
	Category 2	…	…	…
	…			
Age	Category 1	…	…	…
	Category 2	…	…	…
	Category 3	…	…	…
**Demographic and other baseline variables (Baseline and 12 month follow up)**				
Age, years	Mean (SD)	…	…	…
	Missing	…	…	…
Weight, kg	Mean (SD)			
	Missing			
Height, cm	Mean (SD)			
	Missing			
BMI	Mean (SD)			
	Missing			
**Method of Delivery appointment 1 (screening)**				
	Face to face	n (%)	n (%)	n (%)
	Video consultation	n (%)	n (%)	n (%)
	Telephone with video	n (%)	n (%)	n (%)
	Telephone only	n (%)	n (%)	n (%)
**Method of Delivery appointment 2 (12 months follow up)**				
	Face to face	n (%)	n (%)	n (%)
	Video consultation	n (%)	n (%)	n (%)
	Telephone with video	n (%)	n (%)	n (%)
	Telephone only	n (%)	n (%)	n (%)
**Medical History (Baseline and 12 month follow up)**	….	…	…	…
Chronic bronchitis	Yes	…	…	…
Number of COPD exacerbations	Median(IQR)	…	…	…
Number of hospitalisation for COPD	Median(IQR)	…	…	…
Medical History (ICD-10)	List	…	…	…
**Smoking status (Baseline and 12 month follow up)**				
Current smoking status	Current	n (%)	n (%)	n (%)
	Ex-smoker	…	…	…
	Never smoked	…	…	…
Duration of smoking, years	Mean (SD)			
	Missing			
Do you smoke cigarettes	Yes	n (%)	n (%)	n (%)

Number of cigarettes / day	Mean (SD)			
Do you smoke tobacco	Yes	n (%)	n (%)	n (%)
Quantity of tobacco / week	Mean (SD)			
Do you smoke cigars	Yes	n (%)	n (%)	n (%)
Number of cigars / week	Mean (SD)			
Education level	None	n (%)	n (%)	n (%)
	GCSE	n (%)	n (%)	n (%)
	A/AS level	n (%)	n (%)	n (%)
	Degree or higher	n (%)	n (%)	n (%)
	Other	n (%)	n (%)	n (%)
**Medical measurement (Baseline and 12 month follow up)**				
FEV1 :Pre-bronchodilator	Mean (SD)			
FEV1 :Post-bronchodilator	Mean (SD)			
FVC :Pre-bronchodilator	Mean (SD)			
FVC :Post-bronchodilator	Mean (SD)			

		**Colour chart, SM and recue pack (N=)**	**SM and recue pack (N=)**	**Overall (N=xxx)**
**Healthcare Contact (3 month, 6 month, 12 month)**				
Symptoms of COPD	Yes	n (%)	n (%)	n (%)
NHS Service	GP or another	n (%)	n (%)	n (%)
	Practice nurse	n (%)	n (%)	n (%)
	Physiotherapist			
	…			
	…			
	Other	…	…	…
**A&E visits**				
Symptoms of COPD	Yes	n (%)	n (%)	n (%)
Number of visits	Median (IQR)			
Type of admission	Elective	n (%)	n (%)	n (%)
	Emergency	n (%)	n (%)	n (%)
Number of days spent in hospital	Median (IQR)			
Location of Patient during Admission(Mean (SD))	General ward			
	Acute level 1			
	HDU level 2			
	ITU level 3			
Investigation/procedure (Median (IQR))	X-ray			
	CT scan			
	Oxygen therapy			
	Nebuliser			
	Non-invasive ventilation			
	Other			

Appendix D3: Description of the intervention(s)

	Allocated intervention	
	Colour chart, SM and recue pack (N=)	SM and recue pack (N=)
…		

Appendix D4: Adherence to allocated intervention

		Allocated intervention	
Time-point	Received intervention	Colour chart, SM and recue pack (N=)	SM and recue pack (N=)
xxx	Colour chart, SM and rescue pack		
	SM and recue pack		
	None		
	Missing		

For the e-diary sub study population only	Allocated intervention	
	Colour chart, SM and recue pack (N=)	SM and recue pack (N=)
Self-reported AECOPD equal to medical confirmed AECOPD		
Self-reported AECOPD greater than medical confirmed AECOPD		
Self-reported AECOPD less than medical confirmed AECOPD		

Appendix D5: Protocol deviations

	Allocated intervention	
Protocol deviation	Colour chart, SM and recue pack (N=)	SM and recue pack (N=)
…		
…		
…		

Appendix D6: Primary outcome results:

		Allocated Treatment			
		Colour chart, SM and recue pack (N=)	SM and recue pack (N=)	Relative Risk[1](95% CI, p-value	Adjusted RR[1,2] (95% CI , p-value)
	Yes				

**Primary outcome results: Incidence of at least one AECOPD over 12 months post randomisation where patients needed hospitalisation**	No					
	Missing					

[1] Relative Risk<1 favours the Colour chart, SM and rescue pack
[2]
[2] Adjusted comparisons taking into account all minimisation variables.

Appendix D7: Secondary outcomes results

*** Self-assessment of AECOPD, GP confirmed antibiotic and steroid prescription for AECOPD, all cause hospital admission, Mortality, Unscheduled GP visits, Prescription for oral anti-fungal, Antibiotic resistance**

	**Allocated Treatment**			
	**Colour chart, SM and recue pack (N=)**	**SM and recue pack (N=)**	**Relative Risk[1,2](95% CI, p-value**	**Adjusted RR[1,2] (95% CI , p-value)**
**Secondary Outcomes * (every 3 months)**				
Month 3				
Month 6				
Month 9				
Month 12				

[1] Relative Risk<1 favours the Colour chart, SM and rescue pack
[2] Adjusted comparisons taking into account all minimisation variables.

**** Readmission to hospital for AECOPD at 30 and 90 days, Bed days due to AECOPD, Prescription for 2nd course of antibiotics within 14 days of self-reported event**

	**Allocated Treatment**			
	**Colour chart, SM and recue pack (N=)**	**SM and recue pack (N=)**	**Comparison (Mean difference[1] or RR[2]), (95% CI) , p-value**	**Adjusted Comparison[3] (Estimated difference[1] or RR[2]), (95% CI) , p-value**
**Secondary Outcomes ** (every 3 months)**				
Month 3				
Month 6				
Month 9				
Month 12				

[1] For continuous Scores mean difference <0 indicate less issues with Colour chart, SM and rescue pack
[2] Relative Risk<1 favours the Colour chart, SM and rescue pack
[3] Adjusted comparisons taking into account all minimisation variables. Estimated difference <0 indicate less AECOPD hospital admission with Colour chart, SM and rescue pack. For binary outcome, RR<1 favours the Colour chart, SM and rescue pack.

*****COPD Assessment Test (CAT): Cough, Phlegm, Chest, Breath, Limited, Confident, Sleep and Energy**

	**Allocated Treatment**			
	**Colour chart, SM and recue pack (N=)**	**SM and recue pack (N=)**	**Interaction p-value**	**Treatment Effect[1,2] (95% CI)**
**CAT (Mean (SD,n))**				
Screening				
Month 3 (TC2)				

Month 6 (TC3)				
Month 9 (TC4)				
Month 12				

[1] For continuous Scores mean difference <0 indicate less issues with Colour chart, SM and rescue pack

[2] Adjusted comparisons taking into account all minimisation variables including Baseline score. Estimated difference <0 indicate less issues with Colour chart, SM and rescue pack.

Appendix D8: Safety

	**Colour chart, SM and recue pack (N=)**	**SM and recue pack (N=)**	**p-value**
Adverse event 1	n (%)	n (%)	
Adverse event 2	n (%)	n (%)	
…			

	**Colour chart, SM and recue pack (N=)**	**SM and recue pack (N=)**	**p-value**
Total number of SAEs	n	n	
Total number of participants experiencing an SAE	n (%)	n (%)	
Total number of SUSARs	n	n	
Total number of participants experiencing an SUSAR	n (%)	n (%)	

**Summary of SAE**	**Reason for Reporting**	**Causality**	**Action taken**
**Colour chart, SM and recue pack (N=)**			
1 <insert description of SAE>			
2			
3			
4			
**SM and recue pack (N=)**			
1			
2			
3			
4			

Appendix D9: Subgroup and exploratory analysis for primary outcome

Subgroup description	Colour chart, SM and recue pack (N(%))	SM and recue pack (N(%))	Adjusted Comparison[1] (Estimated difference or RR), (95% CI) , p-value	p-value for interaction
**Severity of COPD**				
**Presence of chronic bronchitis**				
**Prior COPD hospitalization…**				
**Age**				
**Co-morbid bronchiectasis**				
**Asthma**				
**Chronic bronchitis**				
**Hospitalisation**				
**GOLD**				
**AECOPD results with respect to blood eosinophils and class of COPD treatment used**				
**Delivery Methods of appointment 1 (screening)**				
**Delivery Methods of appointment 2 (12 months follow up)**				
**EXPLORATORY**				
Education level				

[1] Adjusted comparisons taking into account all minimisation variables. Estimated difference <0 indicate less AECOPD hospital admission with Colour chart, SM and rescue pack. For binary outcome, RR<1 favours the Colour chart, SM and rescue pack.

Appendix D10: Analysis of sub-randomisations – EXACT items

Subgroup description	Colour chart, SM and recue pack (N: Mean(SD))	SM and recue pack (N: Mean(SD))	Adjusted Comparison[1] (Estimated difference), (95% CI) , p-value	
**Total Score**				
**Breathlessness Score**				
**Cough and Sputum Score**				
**Chest Symptoms Score**				
**1. Did your chest feel congested today?**				
Not at all	n(%)	n(%)		
Slightly	n(%)	n(%)		
Moderately	n(%)	n(%)		
Severely	n(%)	n(%)		
Extremely	n(%)	n(%)		
**2. How often did you cough today?**				
Not at all	n(%)	n(%)		
Slightly	n(%)	n(%)		
Moderately	n(%)	n(%)		
Severely	n(%)	n(%)		
Extremely	n(%)	n(%)		
…				
…				
**14. How scared or worried were you about your lung problems today?**				
Not at all	n(%)	n(%)		
Slightly	n(%)	n(%)		
Moderately	n(%)	n(%)		
Severely	n(%)	n(%)		
Extremely	n(%)	n(%)		

[1] Adjusted comparisons taking into account all minimisation variables. Estimated difference <0 indicate less severity with Colour cha rt, SM and rescue pack.
