# Supplementary material for: Sputum colour charts to guide antibiotic self-treatment of acute exacerbation of chronic obstructive pulmonary disease: the Colour-COPD RCT
Source: BMJ Open Respir Res. 2025 Oct 10;12(1):e003615. doi: 10.1136/bmjresp-2025-003615 (PMC12517013; doi:10.1136/bmjresp-2025-003615)
Supplement: online supplemental file 2 [file bmjresp-12-1-s002.pdf]

UNIVERSITY OF BIRMINGHAM | BCTU Birmingham Clinical Trials Unit

Multi-centre randomised trial to determine if the use of sputum colour chart is non-inferior to usual care with respect to hospital admissions: The Colour-COPD Trial

COLOUR COPD

Trial Registration: ISRCTN 14955629

Statistical Analysis Plan

SAP Version Number	Protocol Version Number
2.0	7.0

Name of Author:	Eleni Gkini	Role:	Trial Statistician	Affiliation:	BCTU University of Birmingham
Signature of Author:	E.Gkini	Date:	16 APR 2024		
Name of Chief Investigator:	Dr Alice M Turner	Role:	Chief Investigator	Affiliation:	University of Birmingham
Signature of Chief Investigator:	Alice M Turner	Date:	16/04/2024		
**This Statistical Analysis Plan has been approved by:**					
Name of Approver:	Rajnikant L Mehta	Role:	Senior Statistician	Affiliation:	BCTU University of Birmingham
Signature of Approver:	R Mehta	Date:	17-APR-2024		

SAP version number	SAP section number	Description of and reason for change	Timing of change with respect to interim analysis/ final analysis/ database lock	Blind Reviewer	
2.0	Whole document	Colour-COPD was closed to recruitment by the funder on 31st March 2023 due to recruitment barriers after randomising 115 participants from a required sample size of 2954. Given this can no longer be considered a definitive trial, this has had an impact on the extent of the proposed analyses. The changes described below reflect a pared down approach to the analysis, e.g. less adjustments due reduced numbers of events; sensitivity, apart from those assessing the impact of distributional violations for continuous outcomes and subgroup analyses will no longer be performed.<br><br>A decision was made to stop collecting the Healthcare Economics form at 3, 6 and 9 months and collect only the Healthcare Economics 12 months and hence Quality of life outcomes and self-reported AECOPD outcome will be assessed only at 12 months post-randomisation.<br><br>Moreover, HES data will not be obtained to assess the primary outcome or other secondary outcomes because at the TSC meeting held on 28th February 2022, it was decided that HES data should not be used as a major outcome measure.<br><br>The health economics analysis will no longer be performed.	Prior to database hard lock for the final analysis	Name:	Lee Middleton
				Signature:	Lee Middleton
				Date:	16/4/24
	4.3	Amendment to clarify that the primary outcome will be obtained only from patient reports and recorded on the CRFs.		Name:	Lee Middleton
				Signature:	Lee Middleton

				Date:	16/4/24
	4.4	Amendment to clarify that:<br>• the secondary outcomes (all cause hospital admission, readmission to hospital for AECOPD at 30 and 90 days,) will be obtained only from patient reports and recorded on the CRFs,<br>• the steroid prescriptions outcome will be derived from all cause prescriptions and<br>• the Quality of life outcomes will be assessed only at 12 months post randomisation because after the trial was stopped the healthcare form reporting these outcomes stopped being collected at 3, 6 and 9 months.<br><br>Removal of the secondary outcome: self-reported AECOPD measured repeatedly because after the trial was stopped the healthcare form reporting this outcome stopped being collected at 3, 6 and 9 months.<br><br>Addition of a clarification that the economic evaluation analysis will not be conducted.		Name:<br>Signature:<br>Date:	Lee Middleton<br>Lee Middleton<br>16/4/24
	4.8	Correction to provide a clearer framework.		Name:<br>Signature:<br>Date:	Lee Middleton<br>Lee Middleton<br>16/4/24
	4.9	Amendment of the paragraph to use past tense.		Name:<br>Signature:<br>Date:	Lee Middleton<br>Lee Middleton<br>16/4/24
	4.10	Amendment of the paragraph to use past tense and to remove unnecessary information.		Name:<br>Signature:<br>Date:	Lee Middleton<br>Lee Middleton<br>16/4/24
	4.11	Amendment of the paragraph to include details after the early cessation of the trial.		Name:<br>Signature:	Lee Middleton<br>Lee Middleton

				 Date: 16/4/24
	5.1	Amendment of the paragraph to remove p-values.		Name: Lee Middleton<br>Signature: Lee Middleton<br>Date: 16/4/24
	5.3	Removal of the per protocol analysis population.		Name: Lee Middleton<br>Signature: Lee Middleton<br>Date: 16/4/24
	5.4	Addition of the definition of adherence for the whole trial population.		Name: Lee Middleton<br>Signature: Lee Middleton<br>Date: 16/4/24
	5.5	Amendment to consider all questionnaires valid regardless of the timing of completion		Name: Lee Middleton<br>Signature: Lee Middleton<br>Date: 16/4/24
	9.1	Amendment to remove GP practice from any adjustment due to reduced degrees of freedom.		Name: Lee Middleton<br>Signature: Lee Middleton<br>Date: 16/4/24
	9.2	Addition of bootstrapping methods in case of skewed continuous outcomes.		Name: Lee Middleton<br>Signature: Lee Middleton<br>Date: 16/4/24
	9.3	Removal of the sensitivity analysis to assess the impact of the missing data.		Name: Lee Middleton<br>Signature: Lee Middleton<br>Date: 16/4/24
	9.4	Amendment to move this section in the Appendix E.		Name: Lee Middleton<br>Signature: Lee Middleton

				 Date:	 16/4/24
	9.5	Amendment of the paragraph to remove the mixed effects (GP practice as a random effect) from the analysis models.		Name:<br>Signature:<br>Date:	Lee Middleton<br>Lee Middleton<br>16/4/24
	9.6	Amendment of the analysis of the secondary outcomes.		Name:<br>Signature:<br>Date:	Lee Middleton<br>Lee Middleton<br>16/4/24
	9.7	Amendment of the paragraph to remove exploratory sub-group analysis (educational level) and to add the e-diary exploratory analysis.		Name:<br>Signature:<br>Date:	Lee Middleton<br>Lee Middleton<br>16/4/24
	9.8	Removal of the chi-squared test because safety will be presented only descriptively.		Name:<br>Signature:<br>Date:	Lee Middleton<br>Lee Middleton<br>16/4/24
	9.9	Amendment to remove all the sub-group analyses.		Name:<br>Signature:<br>Date:	Lee Middleton<br>Lee Middleton<br>16/4/24
	9.10	Amendment of the paragraph to include sensitivity analyses for skewed continuous outcomes, for outliers and to remove the per-protocol sensitivity analysis.		Name:<br>Signature:<br>Date:	Lee Middleton<br>Lee Middleton<br>16/4/24
	11	Amendment of the paragraph to remove the health economic analysis after the early cessation of the trial.		Name:<br>Signature:<br>Date:	Lee Middleton<br>Lee Middleton<br>16/4/24
	13	Amendment to include extra references.		Name:	Lee Middleton

				Signature:	Lee Middleton
				Date:	16/4/24
	Appendix E	Amendment of the manipulations section to clarify all outcomes.		Name:	Lee Middleton
				Signature:	Lee Middleton
				Date:	16/4/24

Statistical Analysis Plan (SAP) Amendments

Abbreviations & Definitions	
**Abbreviation / Acronym**	**Meaning**
AECOPD	Acute exacerbation of COPD
aIRR	Adjusted Incidence Rate Ratio
aRD	Adjusted Risk Difference
aRR	Adjusted Risk Ratio
BCTU	Birmingham Clinical Trials Unit
BMI	Body Mass Index
CAT	COPD Assessment Test
CI	Chief Investigator
CONSORT	Consolidated Standards of Reporting Trials
COPD	Chronic Obstructive Pulmonary Disease
CPRD	Clinical Practice Research Database
CRF	Case Report Form
DMC	Data Monitoring Committee
DOB	Date of Birth
EQ-5D-5L	Euroqol 5-Dimension 5-Level
FEV1	Forced Expiratory Volume in 1 second
FVC	Forced Vital Capacity
GEE	Generalised Estimating Equations
GLI	Global Lung Function Index
GP	General Practitioner
HES	Hospital Episode Statistics
HRA	Health Research Authority
HRU	Health Resource Usage
ICF	Informed Consent Form
ICH	International Conference on Harmonisation
ITT	Intention to Treat
ISRCTN	International Standard Randomised Controlled Trial Number
NHS	National Health Service
NICE	National Institute for Health and Care Excellence
PI	Principal Investigator
PIS	Participant Information Sheet
PP	Per-Protocol
QA	Quality Assurance
RCT	Randomised Controlled Trial
REC	Research Ethics Committee
RP	Rescue Pack
RGT	Research Governance Team
SAE	Serious Adverse Event
SAP	Statistical Analysis Plan

SAR	Serious Adverse Reaction
SGRQ	St George's Respiratory Questionnaire
SM	Self-Management
SUSAR	Suspected Unexpected Serious Adverse Reaction
TC	Telephone Call
TSC	Trial Steering Committee
UoB	University of Birmingham
UK	United Kingdom

**Term**	**Definition**
International Standard Randomised Controlled Trial Number	A clinical trial registry
Protocol	Document that details the rationale, objectives, design, methodology and statistical considerations of the study
Randomisation	The process of assigning trial participants to intervention or control groups using an element of chance to determine the assignments in order to reduce bias.
Statistical Analysis Plan	Pre-specified statistical methodology documented for the trial, either in the protocol or in a separate document.

TABLE OF CONTENTS

1. Introduction 11
2. Background and rationale 11
3. Trial objectives 11
4. Trial methods 12
4.1. Trial design 12
4.2. Trial interventions 13
4.3. Primary outcome measure 13
4.4. Secondary outcome measures 13
4.5. Timing of outcome assessments 14
4.6. Randomisation 14
4.7. Sample size 14
4.8. Framework 15
4.9. Interim analyses and stopping guidance 15
4.10. Internal Pilot Progression Rules 15
4.11. Timing of final analysis 15
4.12. Timing of other analyses 16
4.13. Trial comparisons 16
5. Statistical Principles 16
5.1. Confidence intervals and p-values 16
5.2. Adjustments for multiplicity 16
5.3. Analysis populations 16
5.4. Definition of adherence 16
5.5. Handing protocol deviations 17
5.6. Unblinding 17
6. Trial population 17
6.1. Recruitment 17
6.2. Baseline characteristics 17
7. Intervention(s) 18
7.1. Description of the intervention(s) 18
7.2. Adherence to allocated intervention 18
8. Protocol deviations 18
9. Analysis methods 18
9.1. Covariate adjustment 18
9.2. Distributional assumptions and outlying responses 19
9.3. Handling missing data 19
9.4. Data manipulations 19
9.5. Analysis methods – primary outcome 19
9.6. Analysis methods – secondary outcomes 19
9.7. Analysis methods – exploratory outcomes and analyses 21
9.8. Safety data 22
9.9. Planned subgroup analyses 22
9.10. Sensitivity analyses 22
10. Analysis of sub-randomisations 22
11. Health economic analysis 22
12. Statistical software 22
13. References 22
Appendix A: Deviations from SAP 23
Appendix B: Trial schema 24
Appendix C: Schedule of assessments 25
Appendix D1: CONSORT flow diagram 26

Appendix D2: Baseline characteristics 27
Appendix D3: Description of the intervention(s) 30
Appendix D4: Adherence to allocated intervention 30
Appendix D5: Protocol deviations 30
Appendix D6: Primary outcome results: 31
Appendix D7: Secondary outcomes results 31
Appendix D8: Safety 32
Appendix D9: Subgroup and exploratory analysis for primary outcome 34
Appendix D10: Analysis of sub-randomisations – EXACT items 35
Appendix E: Data manipulations 36

1. Introduction

This document is the Statistical Analysis Plan (SAP) for the Colour COPD trial and should be read in conjunction with the current trial protocol. This SAP details the proposed analyses and presentation of the data for the main paper(s) reporting the results for the Colour COPD trial.

The results reported in these papers will follow the strategy set out here. Subsequent analyses of a more exploratory nature will not be bound by this strategy, though they are expected to follow the broad principles laid down here. The principles are not intended to curtail exploratory analysis (e.g. to decide cut-points for categorisation of continuous variables), nor to prohibit accepted practices (e.g. transformation of data prior to analysis), but they are intended to establish rules that will be followed, as closely as possible, when analysing and reporting data.

Any deviations from this SAP will be described and justified in the final report or publication of the trial (using a table as shown in Appendix A: Deviations from SAP). The analysis will be carried out by an appropriately qualified statistician, who should ensure integrity of the data during their data cleaning processes.

2. Background and rationale

The background and rationale for the trial are outlined in detail in the protocol. In brief,

Chronic Obstructive Pulmonary Disease (COPD) is a chronic condition affecting 2 million people in the UK, causing over 140,000 hospital admissions and 1.7% of UK hospital bed days per year (1). Common day to day symptoms include breathlessness, which is typically worse on exertion, and cough productive of sputum. COPD is defined by airflow obstruction on spirometry, this being a ratio less than 0.7 and lower than the lower limit of normal for age in the forced expiratory volume in 1 second (FEV1) and forced vital capacity (FVC) after administration of a bronchodilator.

This study addresses the problem of personalising and thus optimising effectiveness of Acute exacerbation of COPD (AECOPD) management. It is now widely accepted that each year around half of all patients with COPD have frequent AECOPD (≥2 per year (2)), and this is clinically relevant as these patients have poorer prognosis.

Evaluation of approaches to personalising treatment for COPD is a current research priority, as disease heterogeneity is increasingly recognized. AECOPD are an important part of the personalization agenda, given their frequency in the COPD population and the burden that accrues from them to our health economy. In addition they are a particularly important group for study since hospitalization rates are rising (3).

3. Trial objectives

The primary objective is to assess whether use of the 5 point sputum colour chart, alongside a Self-Management (SM) plan and rescue pack (RP) containing 5 days of antibiotic and steroid treatment (the intervention) is safe, as defined by being not substantially worse compared to use of the plan and pack alone (best usual care) for patient hospitalisation admission for AECOPD at 12 months post enrolment (defined by randomisation time point).

Secondary objectives are as follows:

1. Assess whether the intervention is safe, as defined by the rate of 30 and 90 day AECOPD readmissions, rate of treatment failure (defined by ongoing symptoms and/or requirement for treatment in the 14 days after a self-managed event), and time to next AECOPD after a self-managed event

2. Determine whether use of the intervention is effective at 12 months after enrolment in terms of reducing self-reported antibiotic use when compared to best usual care, including rescue pack, as well as reducing adverse events related to antibiotics (e.g. oral thrush).

3. Describe the effect of the intervention on number of unscheduled General Practitioner (GP) attendances, for AECOPD, in the 12 months post enrolment.

4. Describe the effect of the intervention on unreported AECOPD rate through a sub-study using daily symptom diaries. Unreported AECOPD are defined by daily symptom change in the absence of input from a healthcare professional, or reporting symptom change to a healthcare professional. This sub-study will allow us to determine if unreported AECOPD are impacted upon by the intervention, and their rate in UK primary care; it also allows us to assess adherence to the intervention's advice accurately.

5. Describe the effect of the intervention on antibiotic resistance patterns in sputum of people with COPD. This sub-study also allows us to assess the appropriateness of antibiotic use by objectively confirming sputum colour at AECOPD and confirming presence of bacteria.

6. To assess fidelity of delivery of the intervention by use of a checklist inquiring on critical features of education around colour chart and self-management plan use.

7. To assess adherence to SM plan advice by comparing use of AECOPD treatment to daily symptoms (e-diary subgroup only).

8. To explore social acceptability and practical responses to the intervention by interviewing both staff delivering the intervention and participants receiving the intervention.

Economic objectives are as follows:

1. Determine the cost-effectiveness and cost-utility of using a colour chart as part of a SM plan.

4. Trial methods

4.1. Trial design

Colour COPD is a prospective 2 arm, multi-centre, open label, and parallel-group non-inferiority randomised controlled trial investigating the use of Sputum Colour Charts to guide antibiotic self-treatment of acute exacerbation of COPD in patients with COPD. There is an integral pilot, sub-studies of acceptability of the intervention, antibiotic resistance patterns in sputum, daily symptom control and an economic analysis. The study is set in primary care, recruiting from approximately 80 GP practices predominantly within Birmingham and Greater Manchester areas. See Appendix B: Trial schema for trial schema.

Due to the nature of this intervention it is not possible to blind at the patient or investigator level. The research team member making the phone calls to the participants will be blinded to the participant's allocation.

There is a 9 month internal pilot among 50% of practices and 15% of participants (n=444). For further details please refer to section 4.10 and section 2.1 within the protocol.

4.2. Trial interventions

Experimental Arm: use of the 5 point sputum colour chart, adapted from Bronkotest® a SM plan and RP containing 5 days of antibiotic and steroid treatment.

Control Arm: use of the plan and pack alone (best usual care).

4.3. Primary outcome measure

A binary outcome assessing incidence of at least one AECOPD over 12 months post randomisation where patients needed hospitalisation (defined by hospital discharge letter/coding). Incidence of a AECOPD will be obtained from patient reports and recorded on the CRFS.

4.4. Secondary outcome measures

The secondary outcomes are as follows:

- Self-reported antibiotic for AECOPD and all cause steroid prescriptions at 12 months post randomisation
- All cause hospital admission, participant self-report at 12 months post randomisation
- Readmissions to hospital for AECOPD at 30 and 90 days, participant self-report at 12 months post randomisation
- Bed days due to AECOPD at 12 months post randomisation
- Mortality, as determined by the medical record at 12 months post randomisation
- Self-reported GP visits, for AECOPD at 12 months post randomisation
- Self-reported prescriptions for 2nd courses of antibiotics within 14 days of self-reported event (defined as treatment failure) at 12 months post randomisation
- Self-reported prescriptions for anti-fungals (e.g. for oral thrush) at 12 months post randomisation
- Quality of life (COPD assessment test [CAT], Euroqol 5-Dimension 5-Level [EQ-5D-5L]) at 12 months post randomisation:
    - The CAT score can range from 0 to 40 and the total score will be used to compare between groups. There are no subscales within it (4)
    - The total score EQ-5D-5L will be calculated using the mapping function developed by Van Hout et al. (2012) (5) and the Crosswalk value sets for the UK; and it ranges from -0.594 to 1 with -0594 indicates unable to / extreme problems on all of the five dimensions and 1 indicates no problems on any of the five dimensions.
- Antibiotic resistance (determined by sputum culture at baseline, all AECOPD and 12 months)

- Health Resource Usage (HRU); self-reported by participant every 3 months, and submitted using a specific HRU Case Report Form (CRF) (at 3, 6, 9 and 12 months post randomisation respectively).

The EQ-5D-5L total score and the Health Resource Usage (HRU) outcomes were meant to be assessed in the economic evaluation analysis, however, after the trial cessation by NIHR this analysis will not be conducted. Additionally, the self-reported AECOPD assessed repeatedly outcome will not be analysed because the outcome was stopped being selected repeatedly after the trial cessation.

4.5. Timing of outcome assessments

The schedule of trial procedures and outcome assessments are given in Appendix C: Schedule of assessments.

4.6. Randomisation

Participants will be randomised by computer (or telephone if practices have poor online access) at the level of the individual in a 1:1 ratio to either 5 point sputum colour chart, adapted from Bronkotest® colour chart or usual care as described previously, and this will be conducted by the Birmingham Clinical Trials Unit (BCTU) team.

A minimisation algorithm will be used within the online randomisation system to ensure balance in the treatment allocation over the following variables, which centre on factors influencing AECOPD and admission:

- Severity of COPD (see Figure 1 in protocol section 1.1)
    - C: CAT<10, 2 or more exacerbations in the last 12 months OR ≥1 hospital admission for an exacerbation
    - D: CAT≥10, 2 or more exacerbations in the last 12 months OR ≥1 hospital admission for an exacerbation
- Presence or absence of chronic bronchitis
- Prior COPD hospitalization (yes or no within the 12 months prior to enrolment)
- Age, as defined by <65 years, 65-80 years (inclusive), >80 years

In addition, GP practice will be included to adjust for any stratification effects.

A 'random element' will be included in the minimisation algorithm, so that each participant has a probability (unspecified here), of being randomised to the opposite treatment that they would have otherwise received. Full details of the randomisation specification will be stored in a confidential document at BCTU.

4.7. Sample size

We have used hospitalisation rates from the clinical practice research database (CPRD) to determine event rate for our primary outcome.(6) Assuming a 1-sided significance level of 2.5% and a rate of admission in each group of 65% of that in the referenced data, with a non-inferiority margin of 6 percentage points, we would need to enrol 1329 patients in each of the intervention and control groups (2658 in total) to have 90% power for determining whether the results in the usual care group were non-inferior to those in the intervention group. Assuming dropout/lost to follow-up/non-adherence rate of 10% we therefore need to recruit 2954 patients.

4.8. Framework

The objective of the trial is to test the non-inferiority of one intervention to another.

The intervention group will be considered as non-inferior to the usual care group if the upper 95% confidence limit for the absolute risk difference (treated vs usual care) was < 6%.

4.9. Interim analyses and stopping guidance

A separate Data Monitoring Committee (DMC) reporting template was drafted and agreed by the DMC including an agreement on which outcomes would be reported at interim analyses. The statistical methodology stated in this SAP was followed for the outcomes included in the DMC report, where possible.

4.10. Internal Pilot Progression Rules

The trial was designed to have a 9 month internal pilot among 50% of practices and 15% of participants (n=443). Data from these elements form our stop/go criteria.

Accrual of 222 participants per group (Total = 444) in 9 months
At least 40 sites open to accrual at 9 months
At least 50% of sites open to accrual successfully recruiting a participant at 9 months

The internal pilot had three purposes:

(i) To assess recruitment rate and exclusions. We were aiming for 50% of sites enrolling at 9 months from study approval by the relevant authorities and targeted an overall recruitment rate of 6 patients per site per month. This target was an average across all sites, not per site and informs progression.

(ii) To assess spirometry in the GP record, specifically the rate of recording of raw values (which are required alongside age and sex to calculate predicted values), its ease of remote electronic extraction and any inconsistency between coded diagnosis of COPD and spirometry values indicative of airflow obstruction.

(iii) To refine sample size calculation and determine practicality of HES data for this. We were planning to do this by reviewing incidence of hospitalisations for AECOPD and AECOPD rate (including unreported events in the e-diary study) to determine if sample size remains accurate.

See the protocol for or progression criteria based on recruitment and data received.

However, recruitment was very slow and led to early termination of the trial. From June 2021 when trial opened to recruitment until March 2022 only 3 participants had been randomised in Colour-COPD.

4.11. Timing of final analysis

The final analysis for the trial will occur after all randomised participants until March 2023, when the trial stopped, have completed the 1 year assessment and the corresponding outcome data has been entered onto the trial database and validated as being ready for analysis. E-diary and sputum sub-studies are also analysed at

completion of the study. This analysis will include data items up to and including the 1 year assessment and no further.

4.12. Timing of other analyses

Not applicable.

4.13. Trial comparisons

All references in this document to 'group' refer to: 5 point sputum colour chart, adapted from Bronkotest®, alongside a standardised SM plan and rescue pack of 5 days antibiotic and steroid treatment or SM plan and rescue pack alone (best usual care).

5. Statistical Principles

5.1. Confidence intervals and p-values

Estimates for the primary and secondary outcomes will be presented with two-sided 95% confidence intervals only. Safety data will be summarised by number and percentages of participants experienced an event.

5.2. Adjustments for multiplicity

No correction for multiple testing will be made.

5.3. Analysis populations

All primary analyses (primary and secondary outcomes including safety outcomes) will be by intention-to-treat (ITT). Participants will be analysed in the intervention group to which they were randomised, and all participants shall be included whether or not they received the allocated intervention. This is to avoid any potential bias in the analysis.

Further supportive/sensitivity analyses, such as per-protocol analysis or subgroup analysis, will not be presented as the trial was stopped early for recruitment and data are insufficient to perform analyses.

5.4. Definition of adherence

After changes to data collection due to early stopping of the trial, adherence with the protocol for the whole trial population was impossible to be verified by the site research team in terms of whether the participant received no treatment, their randomised allocation or the non-allocated treatment.

We will explore adherence as how well participants adhere to advice in their SM plan in the e-diary sub study where daily symptoms are collected. Specifically, we will examine the frequency of unreported AECOPD, i.e. events where symptoms occur but patients do not report an AECOPD and do not take treatment for it. We will

also use the self-reported AECOPD data compared to the medically confirmed AECOPD data in the whole trial population to infer this; if self-reported events are greater than confirmed events this implies events for which treatment is not taken, and thus potentially poor adherence to the SM plan.

5.5. Handing protocol deviations

A protocol deviation is defined as a failure to adhere to the protocol such as errors in applying the inclusion/exclusion criteria, the incorrect intervention being given, incorrect data being collected or measured, follow-up visits outside the visit window or missed follow-up visits. We will apply a strict definition of the ITT principle and will include all participants as per the ITT population described in section 5.3 in the analysis, in some form, regardless of deviation from the protocol.(7) This does not include those participants who have specifically withdrawn consent for the use of their data in the first instance; however, these outcomes will be explored as per other missing responses.

All questionnaires will be considered valid regardless of the timing of completion.

5.6. Unblinding

Not applicable, Colour-COPD is an open-label study.

6. Trial population

6.1. Recruitment

A flow diagram [as recommended by CONSORT (8)] will be produced to describe the participant flow through each stage of the trial. This will include information on the number (with reasons) of losses to follow-up (drop-outs and withdrawals) over the course of the trial. A template for reporting this is given in Appendix D1: CONSORT flow diagram.

6.2. Baseline characteristics

The trial population will be tabulated as per Appendix D2: Baseline characteristics. Categorical data will be summarised by number of participants, counts and percentages. Continuous data will be summarised by the number of participants, mean and standard deviation if deemed to be normally distributed or number of participants, median and interquartile range if data are skewed. Tests of statistical significance will not be undertaken, nor confidence intervals presented.(9)

7. Intervention(s)

7.1. Description of the intervention(s)

The intervention is the 5 point sputum colour chart, adapted from Bronkotest® 5 colour sputum chart, issued alongside a standardised SM plan and rescue pack at the time of randomisation only. Instructions on how to use the intervention are available from the manufacturer and are shown briefly in the patient self-management plan, which is standardised. A template for reporting information on the intervention(s) is given in Appendix D3: Description of the intervention(s).

7.2. Adherence to allocated intervention

A cross-tabulation of allocated intervention by the adherence categories stated in section 5.4 will be produced (proportions and percentages). A template for reporting adherence is given in Appendix D4: Adherence to allocated intervention.

8. Protocol deviations

Frequencies and percentages by group will be tabulated for the protocol deviations as per Appendix D5: Protocol deviations.

9. Analysis methods

Intervention groups will be compared using log binomial regression model adjusting for all covariates as specified in section 9.1, where possible.

9.1. Covariate adjustment

In the first instance, intervention effects between groups for all outcomes will be adjusted for the minimisation parameters listed in section 4.6 apart from GP practice, which will be excluded due to insufficient number of events. Categorised continuous variables (e.g. age) will be treated as continuous variables in this adjustment. All covariates will be treated as fixed effects.

Other covariate adjustment will be baseline values for parameters where available (e.g. an analysis of questionnaire scores at 12 months will also include the baseline score as a covariate in the model).

If the log-binomial model fails to converge a Poisson regression model with robust standard errors will be used to estimate the same parameters.(10) In this scenario, the risk difference will be estimated from a logistic regression model, using the standardisation approach. If this also fails to converge, unadjusted estimates will be produced from the log-binomial model. It will be made clear in the final report why this occurred (e.g. not possible due to low event rate/lack of model convergence).

9.2. Distributional assumptions and outlying responses

Distributional assumptions (e.g. normality of data and/or regression residuals for continuous outcomes) will be assessed visually prior to reporting the results of the analysis. Although in the first instance the proposed primary method of estimation in this analysis plan will be followed, if distributional assumptions are considered to be violated, the impact of this will be examined through sensitivity analysis; For continuous outcomes, if responses are considered to be particularly skewed and/or distributional assumptions violated, sensitivity analyses will be performed (see Section 9.10) where medians and interquartile ranges will be reported alongside unadjusted differences in medians (and corresponding 95% confidence intervals) using bootstrapping methods (repetitions=1000, seed=050224). If extreme values are apparent and considered to be affecting the integrity of the analysis, a sensitivity analysis consisting of removing the outlying response(s) and repeating the analysis will be performed. Output from these analyses, if performed, will be described and presented alongside the original analysis (or included, e.g. in appendices) with the excluded values clearly labelled. See section 9.10 for further details regarding sensitivity analyses.

9.3. Handling missing data

Due to the very small sample size, no sensitivity analysis will be performed to examine the potential impact of missing data on the results.

9.4. Data manipulations

See Appendix E: Data manipulations

9.5. Analysis methods – primary outcome

A template for reporting the primary outcome is given in Appendix D6: Primary outcome results:

For the analysis of the primary outcome measure frequencies and percentages by group will summarise the number of participants who had at least one hospital admission due to an AECOPD, and a log-binomial model will be used to estimate an adjusted Risk Ratio (aRR) along with 95% confidence intervals, adjusting for the minimisation variables listed in section 4.6 apart from GP practise (see section 9.1) and the number of hospitalisations for COPD in previous year recorded at baseline as a continuous variable and fixed effect. The adjusted Risk Difference (aRD) will be also presented using the identity link. If covariate adjustment fails, we will follow the procedure reported in Section 9.1 Covariate adjustment.

9.6. Analysis methods – secondary outcomes

A template for reporting the secondary outcomes is given in Appendix D7: Secondary outcomes results.

- *Self-reported antibiotic for AECOPD and all cause steroid prescriptions at 12 months post randomisation*

Number of participants who reported at least one prescription for antibiotic due to an AECOPD will be summarised using frequencies and percentages by group and analysed as per the primary outcome. Moreover, the total number of antibiotic prescriptions due to an AECOPD per participant will be also analysed and medians and interquartile rages (IQR) will be reported alongside an adjusted incidence rate ratio (IRR) (and corresponding 95% confidence intervals) estimated using a Poisson regression model, or if there is evidence of over-dispersion a negative binomial regression model adjusting for the minimisation variables listed in section 4.6 apart from GP practice (see section 9.1). The natural logarithm of time in years from the date of randomisation to the date of trial last appointment will be added as an offset variable to incorporating exposure time.
Number of participants who reported at least one prescription for steroid for any reasons will be summarised using frequencies and percentages by group and analysed as per the primary outcome.

- *All cause hospital admission/ Readmission to hospital for AECOPD (30 days) / Readmission to hospital for AECOPD (90 days), participant self-report at 12 months post randomisation*

  These outcomes will be treated as binary outcomes and analysed as per the primary outcome.

- *Bed days due to AECOPD at 12 months post randomisation*

  Total number of bed days due to an AECOPD per participant among those who had at least one hospital admission will be presented only descriptively and summarised by group using means and standard deviations per group or medians (IQR) if data are skewed.

- *Mortality, as determined by the medical record at 12 months post randomisation*

  The mortality will be analysed using survival analysis techniques: Kaplan Meier curves with log rank test. Furthermore, cox regression model will be fitted to obtain hazard ratios (and corresponding 95% confidence intervals) estimated, adjusting for minimisation variables listed in section 4.6 apart from GP practice (see section 9.1). The time to mortality will be obtained at 12 months post randomisation, in which the date of censoring will be the date of either withdrawal-and patient wishes to withdraw completely i.e. no further data will be collected, loss-to-follow-up, or the exact occurs 12 months from the date of randomisation, whichever occurs first. The difference between this date of censoring and the date of randomisation will be time to censoring. If a participant has been withdrawn, or lost-to-follow-up prior to 12 months from the date of randomisation, they will be classed as a censored observation. If the mortality rate is observed to be low then mortality will be presented only descriptively and summarised by group using frequencies and percentages.

- Self-reported GP visits, for AECOPD at 12 months post randomisation

  For the total number of GP visits due to an AECOPD per participant medians and IQR will be reported alongside an aIRR (and corresponding 95% confidence intervals) estimated using a Poisson regression model, or if there is evidence of over-dispersion a negative binomial regression model adjusting for the minimisation variables listed in section 4.6 apart from GP practice (see section 9.1). The natural logarithm of time in years from the date of randomisation to the date of trial last appointment will be added as an offset variable to incorporating exposure time.

- *Self-reported prescriptions for 2nd courses of antibiotics within 14 days of self-reported event (defined as treatment failure)*

  This outcome will be treated as binary outcomes and analysed as per the primary outcome.

- *Self-reported prescriptions for anti-fungals (e.g. for oral thrush)*

  This outcome will be treated as binary outcomes and analysed as per the primary outcome.

- *QoL (COPD assessment test [CAT], Euroqol 5-Dimension 5-Level [EQ-5D-5L]) at 12 months post randomisation*

  The total CAT and EQ-5D-5L scores at 12 months post-randomisation will be analysed individually using a regression model adjusting for their baseline total scores and minimisation variables listed is section 4.6 apart from GP practice. Means and standard deviations will be reported alongside adjusted mean differences (with the corresponding 95% confidence intervals). The EQ VAS score will be presented descriptively and summarised by group using means and standard deviations per group or medians (IQR) if data are skewed.

- *Antibiotic resistance (from sputum culture at Baseline, First AECOPD and 12 months)*

  The antibiotic resistance outcome will be presented descriptively using simple summary statistics by intervention group (i.e. numbers and percentages for binary data and means (or medians) and standard deviations (or inter-quartile ranges) for continuous normal (or non-normal) data but further exploratory analyses will be conducted by a researcher to explore whether the same isolated antimicrobial was grown at each time-point among those who provided more than two sputum samples and compare antibiotic resistance; and also to assess potential antibiotic resistance with respect the antibiotic type that participants were taking in their rescue medication pack (e.g. beta-lactam, macrolides etc).

- *Health Resource Usage (HRU); self-reported by participant every 3 months, and submitted using a specific HRU CRF*

  Any item listed on the Healthcare economics form, which have not been reported as an individual outcome will be presented using simple summary statistics by intervention group (i.e. numbers and percentages for binary data and means (or medians) and standard deviations (or inter-quartile ranges) for continuous normal (or non-normal) data.

9.7. Analysis methods – exploratory outcomes and analyses

Any data that does not form a pre-specified outcome will be presented using simple summary statistics by intervention group (i.e. frequencies and percentages for binary data and means (or medians) and standard deviations (or inter-quartile ranges) for continuous normal (or non-normal) data.

Exploratory analyses to assess adherence, to explore whether the same isolated antimicrobial was grown at each time-point among those who provided more than two sputum samples and compare antibiotic resistance; and to assess potential antibiotic resistance with respect the antibiotic type that participants were taking in their rescue medication pack (e.g. beta-lactam, macrolides etc.) will be also conducted.

Analysis of e-diary AECOPD using the EXACT score will be conducted by a clinical lecturer. 'E-diary analysis will focus on diary defined AECOPD, both reported and unreported, and will use similar principles to those described for self-reported AECOPD in the secondary outcomes. Diary defined AECOPD will be split into reported and unreported according to methods described in prior trials.(11)

9.8. Safety data

The number and percentage of participants experiencing any adverse events, serious adverse events (SAEs) or Related Unexpected SAEs (RUSAEs) will be presented by intervention group. The total number of SAEs in each group will also be given along with a descriptive table of the events. A template for reporting this safety data is given in Appendix D8: Safety.

9.9. Planned subgroup analyses

Because the trial cessed early and no adequate sample size was collected no subgroup analysis will be performed.

9.10. Sensitivity analyses

The following sensitivity analyses will be limited to continuous outcomes and will consist of:

- A sensitivity analysis to assess distributional assumptions (where applicable, as described in section 9.2).
- A sensitivity analysis to assess outliers (where applicable, as described in section 9.2).

10. Analysis of sub-randomisations

Not Applicable.

11. Health economic analysis

Because the trial cessed early the health economic analysis will not be performed.

12. Statistical software

Statistical analysis will be undertaken in the following statistical software packages: SAS (version 9.4) or STATA (version 12 or higher).

13. References

1. The battle for breath - the impact of lung disease in the UK. London: British Lung Foundation, 2016.
2. Hurst JR, Vestbo J, Anzueto A, Locantore N, Mullerova H, Tal-Singer R, et al. Susceptibility to exacerbation in chronic obstructive pulmonary disease. N Engl J Med. 2010;363(12):1128-38.

3. Stone RA, Holzhauer BJ, Lowe D, Searle L, Skipper E, Welham S, et al. National COPD audit programme. COPD: Who cares matters. London, UK: Royal College of Physicians, 2015 Contract No.: eISBN 978-1-86016-559-7.

4. COPD Assessment Test, User Guide, Expert guidance on frequently asked questions Issue 6: May 2022

5. Ben van Hout, M.F. Janssen, You-Shan Feng, Thomas Kohlmann, Jan Busschbach, Dominik Golicki, Andrew Lloyd, Luciana Scalone, Paul Kind, A. Simon Pickard, Interim Scoring for the EQ-5D-5L: Mapping the EQ-5D-5L to EQ-5D-3L Value Sets, Value in Health, Volume 15, Issue 5, 2012, Pages 708-715, ISSN 1098-3015, https://doi.org/10.1016/j.jval.2012.02.008.

6. Merinopoulou E, Raluy-Callado M, Ramagopalan S, MacLachlan S, Khalid JM. COPD exacerbations by disease severity in England. Int J Chron Obstruct Pulmon Dis. 2016;11:697-709.

7. Gupta SK. Intention-to-treat concept: A review. Perspect Clin Res. 2011;2(3):109-112.

8. Piaggio G, Elbourne DR, Pocock SJ, Evens SJW, Altman DG. Reporting of Noninferiority and Equivalence Randomized Trials. JAMA 2012;V(308), No.24.

9. Altman DG, Dore CJ. Randomisation and baseline comparisons in clinical trials. Lancet 1990; 335:149-53.

10. Zou G. A modified Poisson regression approach to prospective studies with binary data. Am J Epidemiol. 2004;159(7):702-6.

11. Paul W. Jones, Rosa Lamarca, Ferran Chuecos, Dave Singh, Alvar Agustí, Eric D. Bateman, Gonzalo de Miquel, Cynthia Caracta, Esther Garcia Gil. European Respiratory Journal 2014 44: 1156-1165.

12. NICE URL: https://www.nice.org.uk/about/what-we-do/our-programmes/nice-guidance/technology-appraisal-guidance/eq-5d-5l. This link was accessed in Feb 2024.

Appendix A: Deviations from SAP

This report below follows the statistical analysis plan dated *<insert effective date of latest SAP>* apart from following:

**Section of report not following SAP**	**Reason**
<insert section >	<insert, e.g. exploratory analyses request by TMG>

Appendix B: Trial schema

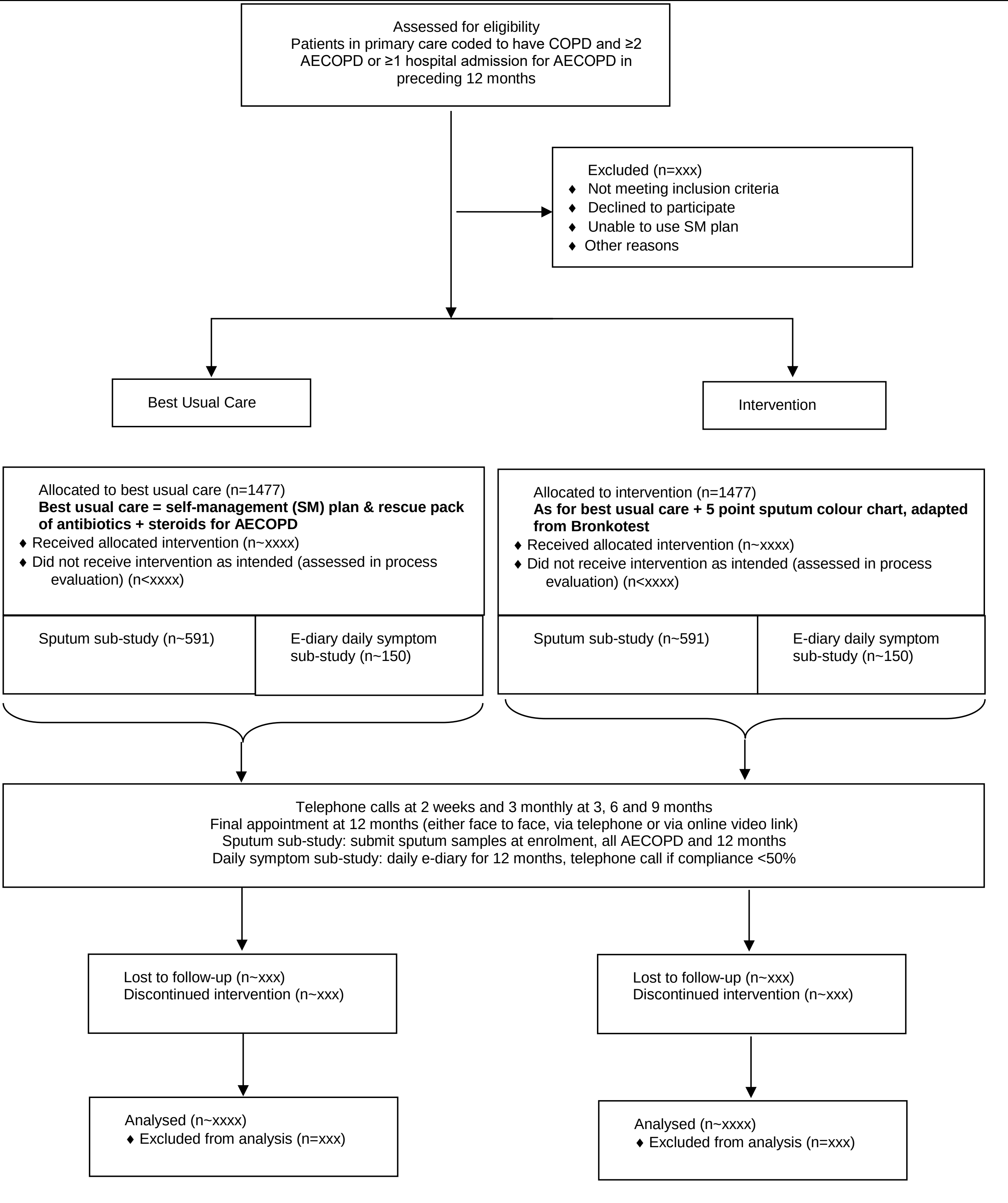

Appendix C: Schedule of assessments

Appointment	Pre-screening	Screening Appointment 1	Telephone Call TC1 *(E-diary sub-study and fidelity checks)*	Telephone Call TC2	Telephone Call TC3	Telephone Call TC4	End of Study Appointment 2
**Weeks**		**Day 0**	**2 weeks**	**3 months**	**6 months**	**9 months**	**12 months**
**Time window for appointment**			**+/- 7 days**	**+/- 4 weeks**	**+/- 4 weeks**	**+/- 4 weeks**	**+/- 2 month**
Review of Inclusion & Exclusion criteria	X	X					
Informed consent		X					
Randomisation		X					
Intervention		X					
Demographics		X					
Medical history		X					
Smoking status		X					X
Concomitant medication		X					X
Educational level		X					
Chronic bronchitis		X					
FEV1 & FVC		X					X
MRC score		X					X
Trial fidelity			X				
Adverse events				X	X	X	X
CAT score		X		X	X	X	X
EQ-5D-5L		X		X	X	X	X
AECOPD rate		X		X	X	X	X
Hospitalisation review		X					X
PROMIS questionnaires					X		

Appendix D1: CONSORT flow diagram

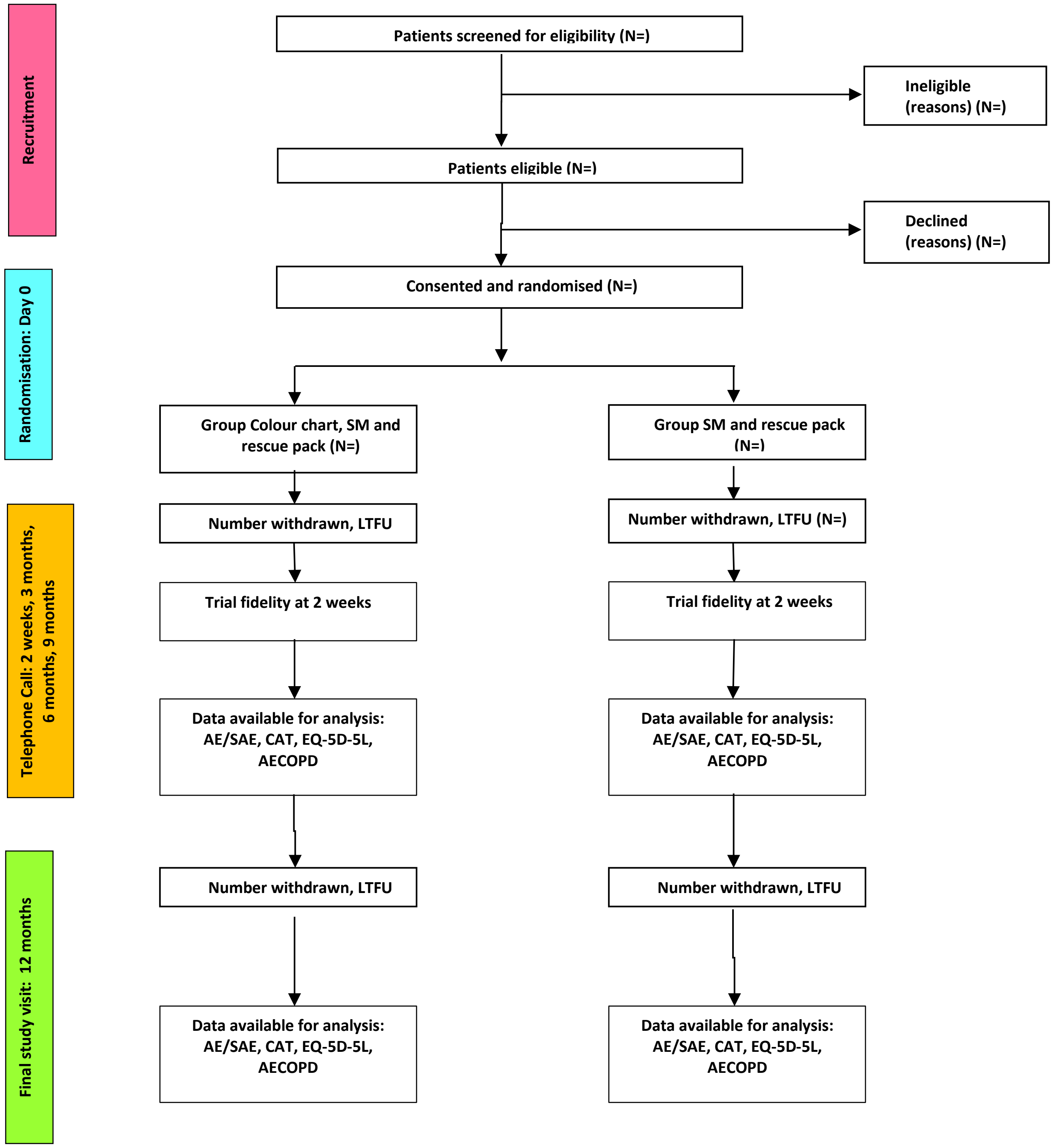

Key:
SM: Self Management
LTFU: Lost to Follow-Up
CAT: COPD assessment test; AE/SAE: Adverse event/Serious adverse event
AECOPD: Acute exacerbation of COPD; EQ-5D-5L

Appendix D2: Baseline characteristics

		Colour chart, SM and rescue pack (N=xxx)	SM and rescue pack (N=xxx)	Overall (N=xxx)
**Minimisation variables**				
Severity of COPD	Category 1	n (%)	n (%)	n (%)
	Category 2	n (%)	n (%)	n (%)
	…	…	…	…
Presence of chronic bronchitis	Category 1	n (%)	n (%)	n (%)
	Category 2	n (%)	n (%)	n (%)
	…	…	…	…
Prior COPD hospitalization	Category 1	…	…	…
	Category 2	…	…	…
	…			
Age	Category 1	…	…	…
	Category 2	…	…	…
	Category 3	…	…	…
**Demographic and other baseline variables (Baseline and 12 month follow up)**				
Age, years	Mean (SD)	…	…	…
	Missing	…	…	…
Weight, kg	Mean (SD)			
	Missing			
Height, cm	Mean (SD)			
	Missing			
BMI	Mean (SD)			
	Missing			
Ethnicity	White-British/English/Northern Irish/Scottish/Welsh	n (%)	n (%)	n (%)
	White-Gypsy/Traveller	n (%)	n (%)	n (%)
	…	…	…	…
Gender	Male	n (%)	n (%)	n (%)
	Female	n (%)	n (%)	n (%)
**Method of Delivery appointment 1 (screening)**				
	Face to face	n (%)	n (%)	n (%)
	Video consultation	n (%)	n (%)	n (%)
	Telephone with video	n (%)	n (%)	n (%)
	Telephone only	n (%)	n (%)	n (%)
**Method of Delivery appointment 2 (12 months follow up)**				
	Face to face	n (%)	n (%)	n (%)
	Video consultation	n (%)	n (%)	n (%)
	Telephone with video	n (%)	n (%)	n (%)
	Telephone only	n (%)	n (%)	n (%)
**Medical History (Baseline and 12 month follow up)**		…	…	…
	….			
Chronic bronchitis	Yes	n (%)	n (%)	…
Number of COPD exacerbations	Median(IQR)	…	…	…

Number of hospitalisation for COPD	Median(IQR)	…	…	…
Medical History (ICD-10)	List	…	…	…
**Smoking status (Baseline and 12 month follow up)**				
Current smoking status	Current	n (%)	n (%)	n (%)
	Ex-smoker	…	…	…
	Never smoked	…	…	…
Duration of smoking, years	Mean (SD)			
	Missing			
Do you smoke cigarettes	Yes	n (%)	n (%)	n (%)
Number of cigarettes / day	Mean (SD)			
Do you smoke tobacco	Yes	n (%)	n (%)	n (%)
Quantity of tobacco / week	Mean (SD)			
Do you smoke cigars	Yes	n (%)	n (%)	n (%)
Number of cigars / week	Mean (SD)			
Education level	None	n (%)	n (%)	n (%)
	GCSE	n (%)	n (%)	n (%)
	A/AS level	n (%)	n (%)	n (%)
	Degree or higher	n (%)	n (%)	n (%)
	Other	n (%)	n (%)	n (%)
**Medical measurement (Baseline and 12 month follow up)**				
FEV1 :Pre-bronchodilator	Mean (SD)			
FEV1 :Post-bronchodilator	Mean (SD)			
FVC :Pre-bronchodilator	Mean (SD)			
FVC :Post-bronchodilator	Mean (SD)			

		**Colour chart, SM and rescue pack (N=)**	**SM and rescue pack (N=)**	**Overall (N=xxx)**
**Healthcare Contact (3 month, 6 month, 12 month)**				
Symptoms of COPD	Yes	n (%)	n (%)	n (%)
NHS Service	GP or another	n (%)	n (%)	n (%)
	Practice nurse	n (%)	n (%)	n (%)
	Physiotherapist			
	…			
	…			
	Other	…	…	…
**A&E visits**				
Symptoms of COPD	Yes	n (%)	n (%)	n (%)
Number of visits	Median (IQR)			
Type of admission	Elective	n (%)	n (%)	n (%)
	Emergency	n (%)	n (%)	n (%)
Number of days spent in hospital	Median (IQR)			
Location of Patient during Admission(Mean (SD))	General ward			
	Acute level 1			
	HDU level 2			
	ITU level 3			
Investigation/procedure (Median (IQR))	X-ray			

CT scan	
Oxygen therapy	
Nebuliser	
Non-invasive ventilation	
Other	

Appendix D3: Description of the intervention(s)

	Allocated intervention	
	Colour chart, SM and rescue pack (N=)	SM and rescue pack (N=)
…		

Appendix D4: Adherence to allocated intervention

		Allocated intervention	
Time-point	Received intervention	Colour chart, SM and rescue pack (N=)	SM and rescue pack (N=)
xxx	Colour chart, SM and rescue pack	n (%)	n (%)
	SM and rescue pack	n (%)	n (%)
	None		
	Missing	n	n

For the e-diary sub study population only	Allocated intervention	
	Colour chart, SM and rescue pack (N=)	SM and rescue pack (N=)
Self-reported AECOPD equal to medical confirmed AECOPD	n (%)	n (%)
Self-reported AECOPD greater than medical confirmed AECOPD	n (%)	n (%)
Self-reported AECOPD less than medical confirmed AECOPD	n (%)	n (%)

Appendix D5: Protocol deviations

	Allocated intervention	
Protocol deviation	Colour chart, SM and rescue pack (N=)	SM and rescue pack (N=)
…	n (%)	n (%)
…	n (%)	n (%)
…	n (%)	n (%)

Appendix D6: Primary outcome results:

Primary Outcome		Allocated Treatment: Colour chart, SM and rescue pack (N=)	Allocated Treatment: SM and rescue pack (N=)	Adjusted Relative Risk[1,2] (95% CI)	Adjusted Risk Difference[3,2] (95%CI)
Number of participants with at least one hospital admission due to AECOPD over 12 months post randomisation	Yes	n (%)	n (%)		
	No	n (%)	n (%)		
	Missing	n	n		

[1] aRR<1 favours the Colour chart, SM and rescue pack.
[2] Adjusted comparisons taking into account all minimisation variables apart from GP practice and number of hospitalisations for COPD in previous year at baseline.
[3] aRD<0 favours the Colour chart, SM and rescue pack.

Appendix D7: Secondary outcomes results

Binary Secondary Outcomes at 12 months	Allocated Treatment: Colour chart, SM and rescue pack (N=)	Allocated Treatment: SM and rescue pack (N=)	Adjusted Relative Risk[1,2] (95% CI)	Adjusted Risk Difference[3,2] (95%CI)
Number of participants with at least one antibiotic prescriptions for AECOPD	n (%)	n (%)		
Number of participants with at least one steroid prescriptions	n (%)	n (%)		
Number of participants with at least one all cause hospital admission[4]	n (%)	n (%)		
Number of participants with at least one prescription for 2nd courses of antibiotics within 14 days of self-reported event (defined as treatment failure)	n (%)	n (%)		
Number of participants with at least one prescription for anti-fungals (e.g. for oral thrush)	n (%)	n (%)		
Number of participants with at least one readmission to hospital for AECOPD at 30 days[4]	n (%)	n (%)		
Number of participants with at least one readmission to hospital for AECOPD at 90 days[4]	n (%)	n (%)		

[1] aRR<1 favours the Colour chart, SM and rescue pack
[2] Adjusted comparisons taking into account all minimisation variables apart from GP practice.
[3] aRD<0 favours the Colour chart, SM and rescue pack.
[4] Also adjusted for number of hospitalisations for COPD in previous year at baseline.

Count Secondary Outcomes at 12 months	Allocated Treatment		
	Colour chart, SM and rescue pack (N=)	SM and rescue pack (N=)	Adjusted IRR[1,2] (95% CI)
Total number of antibiotic prescriptions due to AECOPD per participant	median (IQR)	median (IQR)	
Total number of GP visits due to COPD	median (IQR)	median (IQR)	

[1] aIRR<1 favours the Colour chart, SM and rescue pack
[2] Adjusted comparisons taking into account all minimisation variables apart from GP practice.

Continuous Secondary Outcomes at 12 months	Allocated Treatment		
	Colour chart, SM and rescue pack (N=)	SM and rescue pack (N=)	Adjusted Mean Difference [1,2] (95% CI)
Total CAT score[3]	mean (SD)	mean (SD)	
Total EQ-5D-5L[4]	mean (SD)	mean (SD)	
Bed days due to AECOPD[5]	mean (SD)	mean (SD)	N/A

[1] Adjusted Mean Difference<0 favours the Colour chart, SM and rescue pack
[2] Adjusted comparisons taking into account all minimisation variables apart from GP practice; and the baseline total score.
[3]The CAT score can range from 0 to 40. Higher scores indicate that participants' COPD has a greater impact on their overall health and well-being.
[4]The total score EQ-5D-5L was calculated using the mapping function developed by Van Hout et al. (2012) and the Crosswalk value sets for the UK; and it ranges from -0.594 to 1 with -0594 indicates unable to / extreme problems on all of the five dimensions and 1 indicates no problems on any of the five dimensions.
[5] Among those who had at least one hospital admission.

Continuous Secondary Outcomes at 12 months	Allocated Treatment		
	Colour chart, SM and rescue pack (N=)	SM and rescue pack (N=)	Adjusted Hazard Ratio [1,2] (95% CI)
Mortality	n (%)	n (%)	

[1] Adjusted Hazard Ratio<1 favours the Colour chart, SM and rescue pack
[2] Adjusted comparisons taking into account all minimisation variables apart from GP practice.

Appendix D8: Safety

	Colour chart, SM and rescue pack (N=)	SM and rescue pack (N=)
Total number of SAEs	n	n
Total number of participants experiencing an SAE	n (%)	n (%)
Total number of RUSAEs	n	n
Total number of participants experiencing an RUSAEs	n (%)	n (%)

Summary of SAE	Reason for Reporting	Causality	Action taken
**Colour chart, SM and recue pack (N=)**			
1 <insert description of SAE>			
2			
3			
4			
**SM and recue pack (N=)**			
1			
2			
3			
4			

Appendix D9: Subgroup and exploratory analysis for primary outcome

Not Applicable

Appendix D10: Analysis of sub-randomisations – EXACT items

Not Applicable

Appendix E: Data manipulations

The Trial Statistician will derive all responses from the raw data recorded in the database.

In the following manipulations, if the Healthcare form is missing at any of the 3, 6 or 9 months, it will not affect the manipulations because the data reported in the healthcare form are collected from the last appointment. We also expect not to have collected all Healthcare forms at 3, 6 or 9 months because the trial was stopped early and the data collection process changed for the Healthcare form at 3, 6 or 9 months, which was stopped being collected.

Outcome measures

Primary outcome:

The number of participants who had at least one hospital admission due to an AECOPD is a binary outcome and will be defined as

- 'YES' if at least on time the answer to question: *Since your last study contact, have you been admitted to hospital?* reported on Healthcare form (at 3, 6, 9, or 12 months) is 'YES' AND the primary reason for admission is ('1 – Infective exacerbation COPD' or '2 – non-infective exacerbation COPD') checking all hospital admission reported per participant.
- 'NO' if the answer to all questions: *Since your last study contact, have you been admitted to hospital?* reported on Healthcare form (at 3, 6, 9, or 12 months) is 'NO' OR it is 'YES AND the primary reason for admission is not ('1 – Infective exacerbation COPD' AND '2 – non-infective exacerbation COPD') checking all hospital admission reported per participant.
- 'MISSING' if an answer to question: *Since your last study contact, have you been admitted to hospital?* reported on Healthcare form (at 3, 6, 9, or 12 months) is 'MISSING' and no other hospital admission due to AECOPD is reported, OR if an answer to question: *Since your last study contact, have you been admitted to hospital?* reported on Healthcare form (at 3, 6, 9, or 12 months) is 'YES' but the primary reason for this admission was not provided and no other hospital admission due to AECOPD is reported.

Secondary outcomes:

Self-reported antibiotic prescriptions for AECOPD at 12 months post randomisation

The number of participants who had at least one antibiotic prescription due to AECOPD is a binary outcome and will be defined as

- 'YES' if at least on time the answer to question: *Since your last study contact, have you been prescribed any antibiotics?* reported on Healthcare form (at 3, 6, 9, or 12 months) is 'YES' AND the answer to question: *Were these for an AECOPD?* is also 'YES' checking all antibiotic prescriptions reported per participant.
- 'NO' if the answer to question: *Since your last study contact, have you been prescribed any antibiotics?* reported on Healthcare form (at 3, 6, 9, or 12 months) is 'NO' OR it is 'YES' AND the answer to question: *Were these for an AECOPD?* is also 'NO' checking all antibiotic prescriptions reported per participant.
- 'MISSING' if an answer to question: *Since your last study contact, have you been prescribed any antibiotics?* reported on Healthcare form (at 3, 6, 9, or 12 months) is 'MISSING' and no other antibiotic prescriptions reported due to AECOPD OR if an answer to question: *Since your last study contact, have you been prescribed any antibiotics?* Is 'YES' reported on Healthcare form (at 3, 6, 9, or 12 months) and the answer to question: *Were these for an AECOPD?* is 'MISSING' no other antibiotic prescriptions reported due to AECOPD.

The total number of antibiotic prescriptions due to AECOPD per participant is a count outcome and will be assessed by counting the number of antibiotic prescriptions only for AECOPD reported on the

Healthcare forms at 3, 6, 9, and 12 months by answering the question: *If yes, how many prescriptions for antibiotics have you had since your last study contact?,* among those who reported 'YES' to question: *Since your last study contact, have you been prescribed any antibiotics?,* AND 'YES' to question: *Were these for an AECOPD?,* checking all antibiotic prescriptions per participant.

Self-reported all cause steroid prescriptions at 12 months post randomisation

The number of participants who had at least one steroid prescription is a binary outcome and will be defined as

- 'YES' if at least on time the answer to question: *Since your last study contact, have you been prescribed any steroids?* reported on Healthcare form (at 3, 6, 9, or 12 months) is 'YES'.
- 'NO' if all answers to question: *Since your last study contact, have you been prescribed any steroids?* reported on all Healthcare form (at 3, 6, 9, or 12 months) are 'NO'.
- 'Missing' if the answer to question: *Since your last study contact, have you been prescribed any steroids?* reported on any Healthcare form (at 3, 6, 9, or 12 months) is 'MISSING' and all answers reported on the remaining forms are 'NO'.

All cause hospital admission from participant self-report at 12 months post randomisation

The number of participants who had at least one hospital admission for any reason is a binary outcome and will be defined as

- 'YES' if at least on time the answer to question: *Since your last study contact, have you been admitted to hospital?* reported on Healthcare form (at 3, 6, 9, or 12 months) is 'YES'.
- 'NO' if all answers to question: *Since your last study contact, have you been admitted to hospital?* reported on Healthcare form (at 3, 6, 9, or 12 months) are 'NO'.
- 'Missing' if the answer to question: *Since your last study contact, have you been admitted to hospital?* reported on any Healthcare form (at 3, 6, 9, or 12 months) is 'MISSING' and all answers reported on the remaining forms are 'NO'.

Readmissions to hospital for AECOPD at 30 days from participant self-report at 12 months post randomisation

The number of participants who had at least one readmission to hospital within 30 days due to an AECOPD is a binary outcome and will be defined as

- 'YES' if at least on time, on Healthcare form (at 3, 6, 9, or 12 months), two consecutive hospital admission due to an AECOPD have been reported AND the time in days between these two admissions assessed by the formula Time_days= Date of second hospital admission- Date of first hospital admission, is ≤30.
- 'NO' if either the time in days of the two consecutive hospital admissions, which are described above is >30 OR no readmission is reported.
- 'Missing' if for any of two consecutive hospital admissions, which are described above a date of hospital admission is missing AND (the time in days of the remaining consecutive hospital admissions is >30 OR only these two admissions are reported).

Readmissions to hospital for AECOPD at 90 days from participant self-report at 12 months post randomisation

The number of participants who had at least one readmission to hospital within 90 days due to an AECOPD is a binary outcome and will be defined in the same manner as the outcome of readmissions to hospital for AECOPD at 30 days, with the only difference being that time in days will be compared to 90 days instead of 30 days.

Bed days due to AECOPD at 12 months post randomisation

The total number of bed days is reported on the Healthcare form (at 3, 6, 9, or 12 months) by answering the questions:

- *Since your last study contact, have you been admitted to hospital?*

- *If yes, how many times were you admitted to hospital?*
- *Primary reason for admission*
- *Number of days you spent in hospital*

And it will be derived only among those who reported at least one hospital admission due to an AECOPD by adding the *'number of days you spent in hospital'* for each reported hospital admission due to an AECOPD (when the *Primary reason for admission is* '1 – Infective exacerbation COPD' or '2 – non-infective exacerbation COPD').

Mortality, as determined by the medical record at 12 months post randomisation

Time to death is reported on the SAE form by answering the question:

- *Date of death*

And on randomisation form by answering the question:

- *Date of randomisation*

Time to death (months)=(Date of death – Date of randomisation)/30.4

For any participants leaving the study before the end of the trial, the censoring variables will be reported on the Trial exit/change of status form:

- *Has patient withdrawn from main trial?*
- *Date of withdrawal*
- *Patient wishes to withdraw completely i.e. no further data will be collected*

Or on the healthcare form at 3, 6, 9 or 12 months b y answering the question:

- *Date of trial appointment*

The date of censoring will be the date of either withdrawal (for participants who wishes to withdraw completely), or last trial appointment (for participants lost-to-follow-up), or the exact dated 12 months from the date of randomisation, whichever occurs first. The difference between this date of censoring and the date of randomisation divided by 30.4 will be time to censoring in months.

Self-reported GP visits, for AECOPD at 12 months post randomisation

The total number of GP visits due to an AECOPD is reported on Healthcare form at 3, 6, 9, and 12 months by answering the questions:

- *Date of trial appointment*
- *Since your last trial appointment have you attended the following community NHS services for symptoms related to your COPD? (please do not include any sessions or treatments that you attended as part of the study). For GP visits, please also include appointments that have taken place remotely (via telephone or video)*
- *Number of visits to 'your GP or another GP'*

and on the randomisation form by answering the question:

- *Date of randomisation*

and it will be calculated by adding the number of visits to GP recorded on the 3, 6, 9, 12 months form.
If the answer to the first question is 'NO' then the number of visits to GP will be 0.

Time in years since the last appointment will be calculated by the formula:
Time (years)= (Date of trial appointment - Date of randomisation)/365.25

Self-reported prescriptions for 2nd courses of antibiotics within 14 days of self-reported event (defined as treatment failure) at 12 months post randomisation

The number of participants who had at least one prescriptions for 2nd courses of antibiotics due to an AECOPD within 14 days is a binary outcome and will be defined as

- 'YES' if at least on time, on Healthcare form (at 3, 6, 9, or 12 months), two consecutive prescriptions of antibiotics due to an AECOPD have been reported AND the time in days between these two prescriptions assessed by the formula Time_days= start date of second prescription - start date of first prescription , is ≤14.
- 'NO' if either the time in days of the two consecutive prescriptions, which are described above is >14 OR only one prescription reported.
- 'Missing' if for any of two consecutive prescriptions, which are described above a start date is missing AND (the time in days of the remaining consecutive prescriptions is >14 OR only these two prescriptions are reported).

Self-reported prescriptions for anti-fungals (e.g. for oral thrush) at 12 months post randomisation

The number of participants who had at least one anti-fungals prescription is a binary outcome and will be defined as

- 'YES' if at least on time the answer to question: *Since your last study contact, have you been prescribed any anti-fungals (e.g. for oral thrush)?* reported on Healthcare form (at 3, 6, 9, or 12 months) is 'YES'.
- 'NO' if all answers to question: *Since your last study contact, have you been prescribed any anti-fungals (e.g. for oral thrush)?* reported on all Healthcare form (at 3, 6, 9, or 12 months) are 'NO'.
- 'Missing' if the answer to question: *Since your last study contact, have you been prescribed any anti-fungals (e.g. for oral thrush)?* reported on any Healthcare form (at 3, 6, 9, or 12 months) is 'MISSING' and all answers reported on the remaining forms are 'NO'.

COPD Assessment Test (CAT)

The CAT response scale reported on the Healthcare form at 12 months only are coded as follows:

Question 1: 0-I never cough=0, 1=1, 2=2, 3=3, 4=4, 5=I cough all the time

Question 2: 0-I have no phlegm on my chest at all=0, 1=1, 2=2, 3=3, 4=4, 5=My chest is full of phlegm

Question 3: 0-My chest does not feel right at all=0, 1=1, 2=2, 3=3, 4=4, 5=My chest feels very tight

Question 4: 0-When I walk up a hill or a flight of stairs I am not out of breath=0, 1=1, 2=2, 3=3, 4=4, 5=When I walk up a hill or a flight of stairs I am completely out of breath

Question 5: 0-I am not limited to doing any activity at home =0, 1=1, 2=2, 3=3, 4=4, 5=I am completely limited to doing all activities at home

Question 6: 0-I am confident leaving my home despite my lung condition=0, 1=1, 2=2, 3=3, 4=4, 5=I am not confident leaving my home at all because of my lung condition

Question 7: 0-I sleep soundly=0, 1=1, 2=2, 3=3, 4=4, 5=I do not sleep soundly because of my lung condition

Question 8: 0-I have lots of energy=0, 1=1, 2=2, 3=3, 4=4, 5=I have no energy at all

The CAT total score will be derived by taking the sum of these items. If any of the above questions is missing the outcome will be considered as missing.

Note: Because the healthcare form at 3, 6, and 9 months stopped being collected any possible responses to the CAT questionnaire will not be analysed.

EQ-5D-5L
Reported on the Healthcare form at 12 months by answering the questions:
MOBILITY
SELF-CARE
USUAL ACTIVITIES (e.g. work, study, housework, family or leisure activities)
PAIN / DISCOMFORT
ANXIETY / DEPRESSION
The EQ-5D-5L questionnaire essentially consists of the EQ-5D descriptive system and the EQ visual analogue scale (EQ VAS). The descriptive system comprises five dimensions: mobility, self-care, usual activities, pain/discomfort and anxiety/depression. Each dimension has 5 levels: 1 = no problems, 2 = slight problems, 3 = moderate problems, 4 = severe problems and 5 = extreme problems.
A unique health state is defined by combining one level from each of the five dimensions. A total of 3125 possible health states is defined in this way. Each state is referred to by a 5-digit code. For example, following the order: Mobility, Self-care, Usual activities, Pain/Discomfort, and Anxiety/Depression, state 12345 indicates no problems with mobility, slight problems with washing or dressing, moderate problems with doing usual activities, severe pain or discomfort and extreme anxiety or depression, while state 11111 indicates no problems on any of the five dimensions. For deriving the EQ-5D-5L score, the position statement of the National Institute for Health and Care Excellence (NICE) (12) will be followed, which indicates to derive the 5L score by mapping the 5L descriptive system data onto the 3L value set. No missing data items are permitted in order to compute a score.

Antibiotic resistance (determined by sputum culture at baseline, all AECOPD and 12 months)
All the items being collected in the antibiotic resistant form will be summarised by treatment arms.

Health Resource Usage (HRU); self-reported by participant every 3 months, and submitted using a specific HRU Case Report Form (CRF) (at 3, 6, 9 and 12 months post randomisation respectively).
All the items being collected on the healthcare form at 3, 6, 9, and 12 months resistant form will be summarised by treatment arms.

Other outcomes:

- Age at randomisation = (Randomisation date - date of birth (DOB)) / 365.25
- Body Mass Index (BMI) = Weight (kg) / $[\text{Height (m)}]^2$
