# Supplementary material for: Sputum colour charts to guide antibiotic self-treatment of acute exacerbation of chronic obstructive pulmonary disease: the Colour-COPD RCT
Source: BMJ Open Respir Res. 2025 Oct 10;12(1):e003615. doi: 10.1136/bmjresp-2025-003615 (PMC12517013; doi:10.1136/bmjresp-2025-003615)
Supplement: online supplemental file 3 [file bmjresp-12-1-s003.docx]

**Supplementary material**

**Methods**

***Sputum sub-study***

Data collection

Subjects consented to the sub-study were asked to send a sputum sample at baseline, 12-month follow up and during any exacerbation. Subjects completed a sputum receipt form which requested information on date and time of sample expectoration, contents of rescue pack medication and the colour of their sputum. Intervention subjects were also asked to report their sputum colour according to the 5-point BronkoTest. Samples were posted, by subjects, using Royal Mail SafeBox™ First Class service and were received at the University of Birmingham Research Laboratories at the University Hospitals Birmingham site. Upon receipt, the sputum colour and weight was recorded by laboratory personnel. 0.2g of sample was diluted at 1:1 ratio with dithiothreitol (DTT) and stored at -80oC for further microbiome analysis at a later date. Where sufficient sample was remaining (a minimum of 0.4g), quantitative culture was performed. Sputum was diluted at a 1:1 ratio with DTT and incubated at room temperature for 15 minutes. The sample was then serially diluted, in sterile 0.9% sodium chloride, to give 10-3, 10-4 and 10-5 concentrations. 10ml of each dilution was plated on Columbia Chocolate Agar with 5% Horse Blood (Scientific Laboratory Supplies Ltd, Nottingham, UK). 10ml of 10-4 dilution was also plated on Columbia agar with 5% Horse Blood (Scientific Laboratory Supplies, Nottingham, UK). Where insufficient sputum weight was available for quantitative culture, a primary streak plate was performed using a single loop full of sputum. Agar plates were incubated for 24-48 hours in 5% CO2 at 37oC. The morphology of bacterial isolates was examined and, where a potentially-pathogenic bacteria (PPB) was suspected, an initial Gram stain was performed. The Gram status and shape of bacteria was used to dictate subsequent biochemical identification tests.

Where a PPB was confirmed, antibiotic sensitivity was performed. A bacterial suspension was prepared by inoculating 1ml sterile phosphate-buffered saline with 2-3 colonies. Using a sterile swab, the inoculum was spread equally across the surface of a nutrient agar plate. For Haemophilus and Moraxella species, Iso-Sensitest Agar with Horse Blood and 20mg/L NAD (Fisher Scientific Ltd, Leicester, UK) was used. Antibiotic discs, impregnated with 30mg amoxicillin/clavulanic acid, 10mg ciprofloxacin and 30mg doxycycline (Oxoid Ltd, Reading, UK), were placed equidistantly on the agar. Plates were incubated, as described above, and zones of inhibition were measured for each disc to determine antibiotic susceptibility.

***E-diary sub-study***

Data collection

A sub-study compared self-reported AECOPD in “real-time” between the two study arms using an e-diary among patients who had suitable devices and agreed to take part. Whilst daily diaries can be done on paper they are most reliable and cost-effective when done electronically (1). Our e-diary assessed AECOPD using the EXACT score, symptoms and Anthonisen types of AECOPD (2). The EXACT score reliably detects AECOPD (3). E-diary data therefore described unreported (untreated) AECOPD rates as well as treated events, and potentially could have enhanced power to detect AECOPD rate if these events are numerous. Furthermore it would have provided additional data on patient behaviour with respect to antibiotic use in relation to daily symptoms (for example whether they are taken in a timely manner relative to symptom changes). The EXACT score does not ask about change in sputum colour, we therefore asked this as an additional question, (separate to the EXACT 14-item questionnaire so scoring is unaffected). Intervention participants were asked on the e-diary if they have noticed a change in their sputum colour and what number on their sputum chart represents their sputum colour. They received a colour chart to use alongside this question, provided on a card (as for the whole intervention group) and the colours numbered to ensure that screen settings did not affect interpretation of colour by the patient.

E-diary users were enrolled consecutively from the start, randomising as usual, aiming for n=300 (10% of the total). Patients were approached about the sub-study at appointment 1 and, if eligible, informed consent was obtained. They were asked to complete the e-diary daily; estimated time to complete each day was 3 minutes. Data was linked to a pseudo anonymous patient identifier.

Statistical analysis

All eDiary data was analysed independent of additional data collection to avoid bias on recognition of exacerbations. Baseline patient demographics of those patients enrolled in the eDiary sub study and exacerbations features were summarised; means ±SD were used for normally distributed continuous variables, and medians (IQR) for non-normal continuous variables. Frequency and percentages were used for categorical variables. Follow up was calculated from the first entry to final entry chronologically, with data missingness calculated as all missing values in between these dates.

The eDiary symptom score was calculated from the individual daily responses and compared graphically on an individual basis. Exacerbation episodes were assessed from 14 days before and to 14 days after the start date of an exacerbation. Imputation was used for any missing data, with percentage improvement in the overall missingness of data calculated. A respiratory clinician assessed the daily responses to determine if an exacerbation was likely to have occurred, including whether this was treated with antibiotics and/or prednisolone to identify untreated as well as reported (treated) exacerbations. A proportion of entries were crossed checked against that of another respiratory doctor to ensure agreement. Exacerbation episodes were assessed graphically individually and by mean score across the entire cohort, with means and standard errors of the mean calculated. These scores were then stratified for treated vs untreated exacerbations. Low numbers of participants meant that planned analyses about whether self-management was conducted meaningfully and promptly were not conducted, as no conclusions could be drawn.

***Main trial statistical analysis plan***

All primary analyses (primary and secondary outcomes including safety outcomes) were by intention-to-treat (ITT). Participants were analysed in the intervention group to which they were randomised, and all participants were included whether or not they received the allocated intervention. This is to avoid any potential bias in the analysis. Further supportive/sensitivity analyses, such as per-protocol analysis or subgroup analysis, were not presented as the trial was stopped early and data are insufficient to perform such analyses. Results are displayed as estimates and 95% confidence intervals derived from appropriate log binomial regression model / generalised linear models. Confidence intervals around observed differences were planned to be compared to the pre-specified non-inferiority margin but were not performed due to early cessation. For all outcome measures, appropriate summary statistics are presented by intervention group (frequency count and percentages for categorical data, mean and standard deviation for continuous data). Intervention effects were adjusted for the minimisation variables where possible. No adjustment for multiple comparisons was made.

### Primary Outcome Measure

For the analysis of the primary outcome measure frequencies and percentages by group summarise the number of participants who had at least one hospital admission due to an AECOPD, and a log-binomial model was used to estimate a Risk Ratio (RR) along with 95% confidence intervals. Adjusted comparisons taking into account all minimisation variables, apart from GP practice were performed but resulted to convergence issues and thus were not used. Further analyses of the episodes of AECOPD over 12 months using generalised estimating equations were considered in our plan, but not done due to early cessation. Non-inferiority statistics were not considered due to early cessation.

### Secondary Outcome Measures

*Self-reported antibiotic for AECOPD, all cause steroid prescriptions and total number of GP visits due to COPD at 12 months post randomisation*

Number of participants who reported at least one prescription for antibiotic due to an AECOPD was summarised using frequencies and percentages by group and analysed as per the primary outcome, with the only difference being that the model with the adjusted comparison taking into account all minimisation variables apart from GP practice, converged. Severity of COPD and presence or absence of chronic bronchitis were included as fixed effects; and age at randomisation and number of hospitalisations for COPD in previous year were included as continuous variables. Total number of antibiotic prescriptions due to an AECOPD per participant and total number of GP visits due to COPD are reported alongside an adjusted incidence rate ratio (IRR) (and corresponding 95% confidence intervals) estimated using a negative binomial regression model due to evidence of over-dispersion, adjusting for the minimisation variables as before. The natural logarithm of time in years from the date of randomisation to the date of trial last appointment was added as an offset variable to incorporating exposure time. Number of participants who reported at least one prescription for steroid was analysed in a similar way.

*All cause hospital admission/ Readmission to hospital for AECOPD (30 days) / Readmission to hospital for AECOPD (90 days)*, *participant self-report at 12 months post randomisation*

These outcomes were treated as binary outcomes and analysed as per the primary outcome.

*Bed days due to AECOPD* *at 12 months post randomisation*

Total number of bed days due to an AECOPD per participant among those who had at least one hospital admission was presented only descriptively and summarised by group using medians and Interquartile range (IQR) because the data were deemed skewed.

*Mortality, as determined by the medical record* *at 12 months post randomisation*

Mortality was not analysed due to lack of events. Since the mortality rate was observed to be low, mortality is presented only descriptively and summarised by group using frequencies and percentages.

*Self-reported prescriptions for 2nd courses of antibiotics within 14 days of self-reported event (defined as treatment failure) and for anti-fungals*

This outcome was treated as binary outcomes and analysed as per the primary outcome.

*QoL (COPD assessment test [CAT], Euroqol 5-Dimension 5-Level [EQ-5D-5L]) at 12 months post randomisation*

The total CAT and EQ-5D-5L scores at 12 months post-randomisation were analysed individually using a regression model adjusting for baseline total scores and minimisation variables as before. Means and standard deviations are reported alongside adjusted mean differences (with the corresponding 95% confidence intervals). The EQ VAS score is presented descriptively and summarised by group using means and standard deviations per group or medians (IQR) if data are skewed.

*Health Resource Usage (HRU) and any other data*

This is presented using simple summary statistics by intervention group (i.e. numbers and percentages for binary data and means (or medians) and standard deviations (or inter-quartile ranges) for continuous normal (or non-normal) data.

**Results**


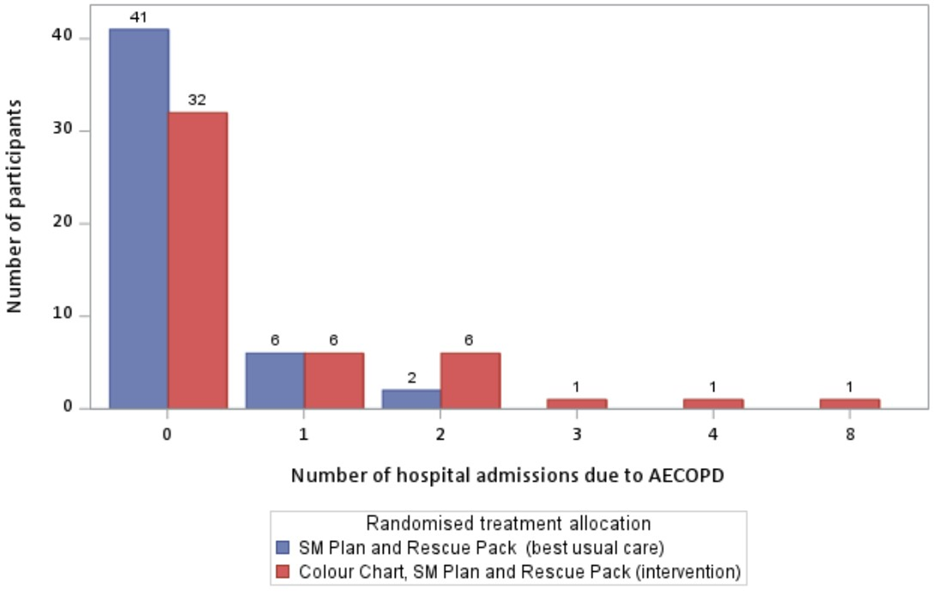


Figure S1: Number of hospital admissions per patient

| **Binary Secondary Outcomes at 12 months** |  | **Allocated Treatment** | | **Adjusted**  **Relative Risk^1,2,3^**  **(95% CI)** | **Adjusted Risk Difference^2,3,4^ (95%CI)** |
| --- | --- | --- | --- | --- | --- |
|  |  | **Colour Chart, SM Plan and Rescue Pack (intervention)**  **N=57** | **SM Plan and Rescue Pack**  **(best usual care)**  **N=58** |  |  |
| **Number of participants with at least one antibiotic prescription for AECOPD** | Yes/N (%) | 37/46  (80) | 43/50  (86) | 0.98  (0.82 to 1.16) | -0.02  (-0.17 to 0.12) |
| **Number of participants with at least one steroid prescription** | Yes/N (%) | 38/46  (83) | 42/50  (84) | 1.02  (0.86 to 1.20) | 0.01  (-0.13 to 0.15) |
| **Number of participants with at least one all cause hospital admission** | Yes/N (%) | 19/47  (40) | 14/49  (29) | 1.47  (0.85 to 2.54)^5^ | 0.11  (-0.07 to 0.29)^6^ |
| **Number of participants with at least one prescription for 2^nd^ course of antibiotics for AECOPD within 14 days** | Yes/N (%) | 15/44  (34) | 8/44  (18) | 1.80  (0.85 to 3.79)^5^ | 0.15  (-0.03 to 0.33)^6^ |
| **Number of participants with at least one prescription for anti-fungals (e.g. for oral thrush)** | Yes/N (%) | 6/45  (13) | 5/48  (10) | 1.19  (0.37 to 3.89)^5^ | 0.02  (-0.11 to 0.16)^6^ |
| **Number of participants with at least one readmission to hospital for AECOPD at 30 days** | Yes/N (%) | 3/47  (6) | 0/49  (0) | Analysis was not performed due to lack of events | |
| **Number of participants with at least one readmission to hospital for AECOPD at 90 days** | Yes/N (%) | 4/47  (8) | 0/49  (0) | Analysis was not performed due to lack of events | |

Table S1: Binary secondary outcome summary statistics

*^1^*aRR<1 favours the Colour Chart, SM Plan and Rescue Pack (intervention).

^2^Log-Binomial regression model.

^3^Adjusted comparisons taking into account all minimisation variables apart from GP practice. Severity of COPD and presence or absence of chronic bronchitis were included as fixed effects; and age at randomisation and number of hospitalisations for COPD in previous year were included as continuous variables.

^4^aRD<0 favours the Colour Chart, SM Plan and Rescue Pack (intervention).

^5^Instead of a log-Binomial regression model, a Poisson regression model with robust error variance was used due to convergence issues.

^6^Instead of a Binomial regression model with identity link function, a logistic regression model with robust standards errors was used, followed by the standardisation approach for covariate adjustment.

| **Count Secondary Outcomes at 12 months** |  | **Allocated Treatment** | | **Adjusted IRR^1,2,3^**  **(95% CI)** |
| --- | --- | --- | --- | --- |
|  |  | **Colour Chart, SM Plan and Rescue Pack (intervention)**  **N=57** | **SM Plan and Rescue Pack**  **(best usual care)**  **N=58** |  |
| **Total number of antibiotic prescriptions due to AECOPD per participant^3^** | Median  [N; IQR] | 2  [47; 1, 4] | 3  [52; 1 , 4] | 1.03^4^ (0.73 to 1.43) |
| **Total number of GP visits due to COPD** | Median  [N; IQR] | 2  [46; 0 , 5] | 1  [51; 0 , 4] | 1.28^5^ (0.69 to 2.39) |

Table S2: Count based secondary outcome summary statistics

^1^aIRR<1 favours the Colour chart, SM and rescue pack

^2^Adjusted comparisons taking into account all minimisation variables apart from GP practice. Severity of COPD and presence or absence of chronic bronchitis were included as fixed effects; and age at randomisation and number of hospitalisations for COPD in previous year were included as continuous variables.

^3^Three participants for whom the binary outcome: Number of participants with at least one antibiotic prescription for AECOPD was missing because they were not followed up for 12 months were included in the outcome: Total number of antibiotic prescriptions due to AECOPD per participant until the time point they were followed up.

^4^Because there was evidence of over-dispersion, it was considered more appropriate to use a negative binomial regression model. Dispersion parameter (95% CI)= 0.32 (0.17 to 0.62) with p-value<0.001 according to the L likelihood-ratio (LR) test.

^5^Because there was evidence of over-dispersion, it was considered more appropriate to use a negative binomial regression model. Dispersion parameter (95% CI)= 1.81 (1.19 to 2.75) with p-value<0.001 according to the L likelihood-ratio (LR) test.

| **Continuous Secondary Outcomes at 12 months** | | **Allocated Treatment** | | **Adjusted Mean Difference^1^**  **(95% CI)** |
| --- | --- | --- | --- | --- |
|  |  | **Colour Chart, SM Plan and Rescue Pack (intervention)**  **N=57** | **SM Plan and Rescue Pack**  **(best usual care)**  **N=58** |  |
| **Total CAT score^2,3^** | Mean (N, SD) | 19.9 (30, 8.4) | 24.5 35, (6.0) | -2.95  (-5.93 to -0.04) |
| **Total EQ-5D-5L score^4,5^** | Mean (N, SD) | 0.57 (27, 0.30) | 0.54 (33, 0.24) | 0.06  (-0.09 to 0.15) |
| **EQ VAS score** | Mean (N, SD) | 49.0 (26, 22.8) | 56.1 (32, 22.9) | N/A |
| **Bed days due to AECOPD^6^** | Number of participants ≥1 hospitalisation for AECOPD | 15 | 8 |  |
|  | Median [N; IQR] | 7.0  [13; 6.0 , 12.0] | 6.5  [6; 5.0 , 15.0] | N/A |

Table S3: Continuous secondary outcomes summary statistics

^1^Adjusted Mean Difference<0 favours the Colour chart, SM and rescue pack (intervention).

^2^Adjusted comparisons taking into account all minimisation variables apart from GP practice. Severity of COPD and presence or absence of chronic bronchitis were included as fixed effects; and age at randomisation, number of hospitalisations for COPD in previous year and baseline CAT score were included as continuous variables.

^3^The CAT score can range from 0 to 40. Higher scores indicate that participants’ COPD has a greater impact on their overall health and well-being.

^4^The total score EQ-5D-5L was calculated using the mapping function developed by Van Hout et al. (2012) and the Crosswalk value sets for the UK; and it ranges from -0.594 to 1 with -0594 indicates unable to / extreme problems on all of the five dimensions and 1 indicates no problems on any of the five dimensions.

^5^Adjusted comparisons taking into account all minimisation variables apart from GP practice. Severity of COPD and presence or absence of chronic bronchitis were included as fixed effects; and age at randomisation, number of hospitalisations for COPD in previous year and baseline EQ-5D-5L score were included as continuous variables.

^6^Among those who had at least one hospital admission due to AECOPD.

^7^Four participants who were included in the primary outcome because they reported at least one hospital admission due to an AECOPD were not included in the bed days outcome because this detail was not collected on the SAE form


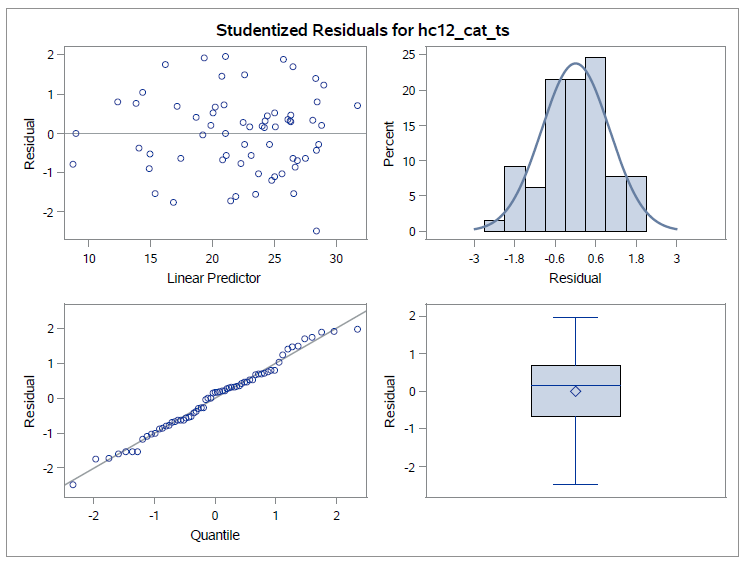


Figure S2: Studentised regression residuals assessing CAT scores’ distributional assumptions


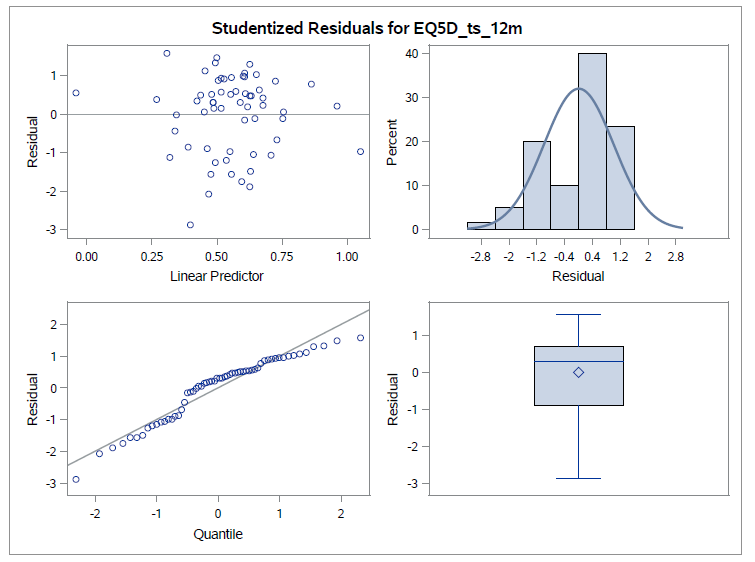


Figure S3: Studentised regression residuals assessing EQ-5D-5L scores’ distributional assumptions

| **Summary of SAE** | **Severity** | **Life threatening** | **Relatedness** | **Expectedness** |
| --- | --- | --- | --- | --- |
| **Colour chart** | | | |  |
| Stroke | Severe | No | Unrelated | Unexpected |
| Small Cell Lung Cancer | Fatal | Yes | Unrelated | Unexpected |
| Constipation | Moderate | No | Unrelated | Unexpected |
| Covid | Severe | No | Unrelated | Unexpected |
| Heart failure | Moderate | No | Unrelated | Expected |
| Cor pulmonale | Moderate | No | Unrelated | Expected |
| Musculoskeletal chest pain | Moderate | No | Unrelated | Unexpected |
| Metastatic breast cancer | Fatal | . | Unrelated | Unexpected |
| Shortness of breath | Moderate | No | Unrelated | Unexpected |
| Drug reaction (rash) | Mild | No | Unrelated | Unexpected |
| **Usual care** | | | | |
| Heart failure | Severe | No | Unrelated | Expected |
| Covid | Severe | No | Unrelated | Unexpected |
| Heart block | Mild | Yes | Unrelated | Unexpected |
| DVT | Moderate | No | Unrelated | Unexpected |
| Heart failure | Moderate | No | Unrelated | Expected |
| Heart failure | Moderate | No | Unrelated | Unexpected |
| Covid | Fatal | No | Unrelated | Expected |

Table S4: Serious adverse events

***Sputum sub-study***

36 samples contained only mixed normal flora of the upper respiratory tract and a further 4 samples were missing lab receipt form data. 10/42 (24%) stable samples were positive for a PPB, including 4 Moraxella catarrhalis, 3 Pseudomonas species, 2 Haemophilus influenzae and 1 Staphylococcus aureus. Of the 15 exacerbation samples, 7 were positive for a PPM (47%): 3 Moraxella catarrhalis, 1 Haemophilus influenzae, 1 Pseudomonas spp and 2 yeast.Antibiotic sensitivity was performed on 13 bacterial isolates. 4/13 (31%) isolates displayed resistance to at least one antibiotic. 2/4 instances of resistance were to amoxicillin/clavulanic acid (1 *H. influenzae* and 1 *Citrobacter* freundii) and 1/4 to doxycycline (*Proteus spp.*). 1/4 (*Pseudomonas spp.*) was resistant to both amoxiclav and doxycycline. In only 1 case, the recovered pathogen was not susceptible to the antibiotic in the patient’s rescue pack. There was no difference in resistance between trial arms.

|  | **Colour Chart, SM Plan and Rescue Pack (intervention)** | **SM Plan and Rescue Pack (best usual care)** | **Total** |
| --- | --- | --- | --- |
|  | **N=19** | **N=19** | **N=38** |
| **Demographic and other baseline variables** |  |  |  |
| Age at randomisation (years) |  |  |  |
| Mean (SD, N) | 66.7 (7.3, 19) | 65.9 (10.5, 19) | 66.3 (8.9, 38) |
| Gender, n (%) |  |  |  |
| Male | 10 (53%) | 11 (58%) | 21 (55%) |
| Female | 9 (47%) | 8 (42%) | 17 (45%) |
| Ethnicity, n (%) |  |  |  |
| White- British/English/Northern Irish/Scottish/Welsh | 19 (100%) | 17 (89%) | 36 (95%) |
| Asian and Asian British- Indian | 0 (0%) | 1 (5%) | 1 (3%) |
| Black and Black British- African Caribbean | 0 (0%) | 1 (5%) | 1 (3%) |
| BMI (kg/m²) |  |  |  |
| Mean (SD, N) | 27.4 (6.8, 19) | 27.5 (6.5, 19) | 27.5 (6.6, 38) |
| Education level, n (%) |  |  |  |
| No formal education | 5 (26%) | 5 (26%) | 10 (26%) |
| GCSE, CSE, O level or equivalent | 8 (42%) | 8 (42%) | 16 (42%) |
| A-level/AS level or equivalent | 2 (11%) | 2 (11%) | 4 (11%) |
| Degree level or higher | 4 (21%) | 2 (11%) | 6 (16%) |
| Other please specify | 0 (0%) | 2 (11%) | 2 (5%) |
| **Medical History (Baseline)** |  |  |  |
| Hospitalisations for COPD in previous year |  |  |  |
| Median [IQR, N] | 0.0 [0.0-2.0,19] | 0.0 [0.0-0.0,19] | 0.0 [0.0-1.0,38] |
| Most recent eosinophil count |  |  |  |
| Mean (SD, N) | 0.2 (0.1, 12) | 0.2 (0.1, 14) | 0.2 (0.1, 26) |
| Chronic asthma, n (%) | 4 (21%) | 5 (26%) | 9 (24%) |
| Bronchiectasis, n (%) | 7 (37%) | 5 (26%) | 12 (32%) |
| Diabetes, n (%) | 1 (5%) | 5 (26%) | 6 (16%) |
| CVA/Stroke/TIA, n (%) | 1 (5%) | 1 (5%) | 2 (5%) |
| Osteoporosis, n (%) | 6 (32%) | 2 (11%) | 8 (21%) |
| Hypertension, n (%) | 4 (21%) | 9 (47%) | 13 (34%) |
| Arthritis, n (%) | 6 (32%) | 6 (32%) | 12 (32%) |
| Coronary Heart Disease, n (%) | 2 (11%) | 3 (16%) | 5 (13%) |
| Depression/Anxiety, n (%) | 4 (21%) | 7 (37%) | 11 (29%) |
| GORD, n (%) | 7 (37%) | 5 (26%) | 12 (32%) |
| **Smoking status (Baseline)** |  |  |  |
| Current smoking status, n (%) |  |  |  |
| Current smoker | 4 (21%) | 4 (21%) | 8 (21%) |
| Ex-smoker | 9 (47%) | 13 (68%) | 22 (58%) |
| Never smoked | 6 (32%) | 2 (11%) | 8 (21%) |
| Duration of smoking (years) |  |  |  |
| Mean (SD, N) | 41.2 (12.5, 12) | 35.8 (18.6, 17) | 38.0 (16.3, 29) |
| **Medical measurement (Baseline)** |  |  |  |
| FEV₁: Pre-bronchodilator (liters) |  |  |  |
| Mean (SD, N) | 1.5 (0.7, 8) | 1.2 (0.4, 11) | 1.3 (0.6, 19) |
| FEV₁: Post-bronchodilator (liters) |  |  |  |
| Mean (SD, N) | 1.7 (0.5, 11) | 1.6 (0.7, 11) | 1.7 (0.6, 22) |
| FVC: Pre-bronchodilator (liters) |  |  |  |
| Mean (SD, N) | 3.7 (1.5, 7) | 2.9 (1.0, 11) | 3.2 (1.2, 18) |
| FVC: Post-bronchodilator (liters) |  |  |  |
| Mean (SD, N) | 3.1 (0.6, 10) | 3.0 (1.0, 11) | 3.0 (0.8, 21) |
| MRC Breathlessness Scale, n (%) |  |  |  |
| Grade 2 | 5 (26%) | 2 (11%) | 7 (18%) |
| Grade 3 | 4 (21%) | 6 (32%) | 10 (26%) |
| Grade 4 | 8 (42%) | 9 (47%) | 17 (45%) |
| Grade 5 | 2 (11%) | 2 (11%) | 4 (11%) |

Table S5: Characteristics of sputum study participants

***E-diary sub-study***

The median completion percentage of the eDiary from the day of first entry to the day of last entry was 49.4% [1^st^ quartile 36.7, 3^rd^ quartile 70.8], with an improvement of median data completion to 71.6% after imputation. Median follow up was 10.6 months [1^st^ quartile 4.3, 3^rd^ quartile 12.0]. One participant withdrew from the study early due to the perceived burden of study questionnaires and another stopped the sub-study early as he thought it had ended.

Treated exacerbations tended to have a lower baseline symptom score and a higher rise in symptoms score compared to untreated exacerbations. For example, the day prior to a symptoms defined exacerbation (Day -1) had a mean symptom score of 29.4 (±5.7) for untreated episodes vs 20.5 (±5.9). This may suggest that patients with a higher burden of symptoms day to day may experience symptom defined exacerbations but fail to recognise their significance. There was also a higher symptom peak for treated exacerbations with a difference of 7.6 compared to 5.5 in untreated episodes, which likely informed the decision to treat. There was a trend towards a slower recovery in patients with untreated exacerbations. For the results in Figure S2, there were no statistically significant differences due to low patient numbers, though the trend aligns with data from a previous study of exacerbations in AATD related lung disease (4).

|  | **Colour Chart, SM Plan and Rescue Pack (intervention)** **N = 5** | **SM Plan and Rescue Pack (best usual care)**  **N = 5** | **Total**  **N = 10** |
| --- | --- | --- | --- |
| Age (years) | 68.6 (4.6, 5) | 65.7 (4.0, 5) | 67.1 (4.3, 10) |
| Male (%) | 4 (80%) | 3 (60%) | 7 (70%) |
| BMI (kg/m²) | 27.8 (6.3, 5) | 27.3 (8.2, 5) | 27.5 (6.9, 10) |
| Current smoking status, n (%) |  |  |  |
| Current smoker | 0 (0%) | 1 (20%) | 1 (10%) |
| Ex-smoker | 4 (80%) | 4 (80%) | 8 (80%) |
| Never smoked | 1 (20%) | 0 (0%) | 1 (10%) |
| Duration of smoking (years) | 43.8 (15.5, 4) | 42.6 (11.5, 5) | 43.1 (12.5, 9) |
| Cigarettes per day | 25.0 (., 1) | 11.7 (7.6, 3) | 15.0 (9.1, 4) |
| COPD GOLD stage |  |  |  |
| Category C^1^ | 2 (40%) | 0 (0%) | 2 (20%) |
| Category D^2^ | 3 (60%) | 5 (100%) | 8 (80%) |
| Most recent blood eosinophils | 0.2 (0.1, 3) | 0.2 (0.1, 5) | 0.2 (0.1, 8) |
| FEV1: Post-bronchodilator (L) | 1.8 (., 1) | 1.6 (., 1) | 1.7 (0.1, 2) |
| FVC: Pre-bronchodilator (L) | 3.9 (0.9, 4) | 2.9 (., 1) | 3.7 (0.9, 5) |
| MRC Breathlessness Scale, n (%) |  |  |  |
| Grade 2 | 3 (60%) | 0 (0%) | 3 (30%) |
| Grade 3 | 0 (0%) | 1 (20%) | 1 (10%) |
| Grade 4 | 2 (40%) | 4 (80%) | 6 (60%) |

Table S6: Characteristics of e-diary study participants

^1^CAT<10, 2 or more exacerbations in the last 12 months OR 1 hospital admission for an exacerbation.

^2^CAT>=10, 2 or more exacerbations in the last 12 months OR 1 hospital admission for an exacerbation.

exacerbation.
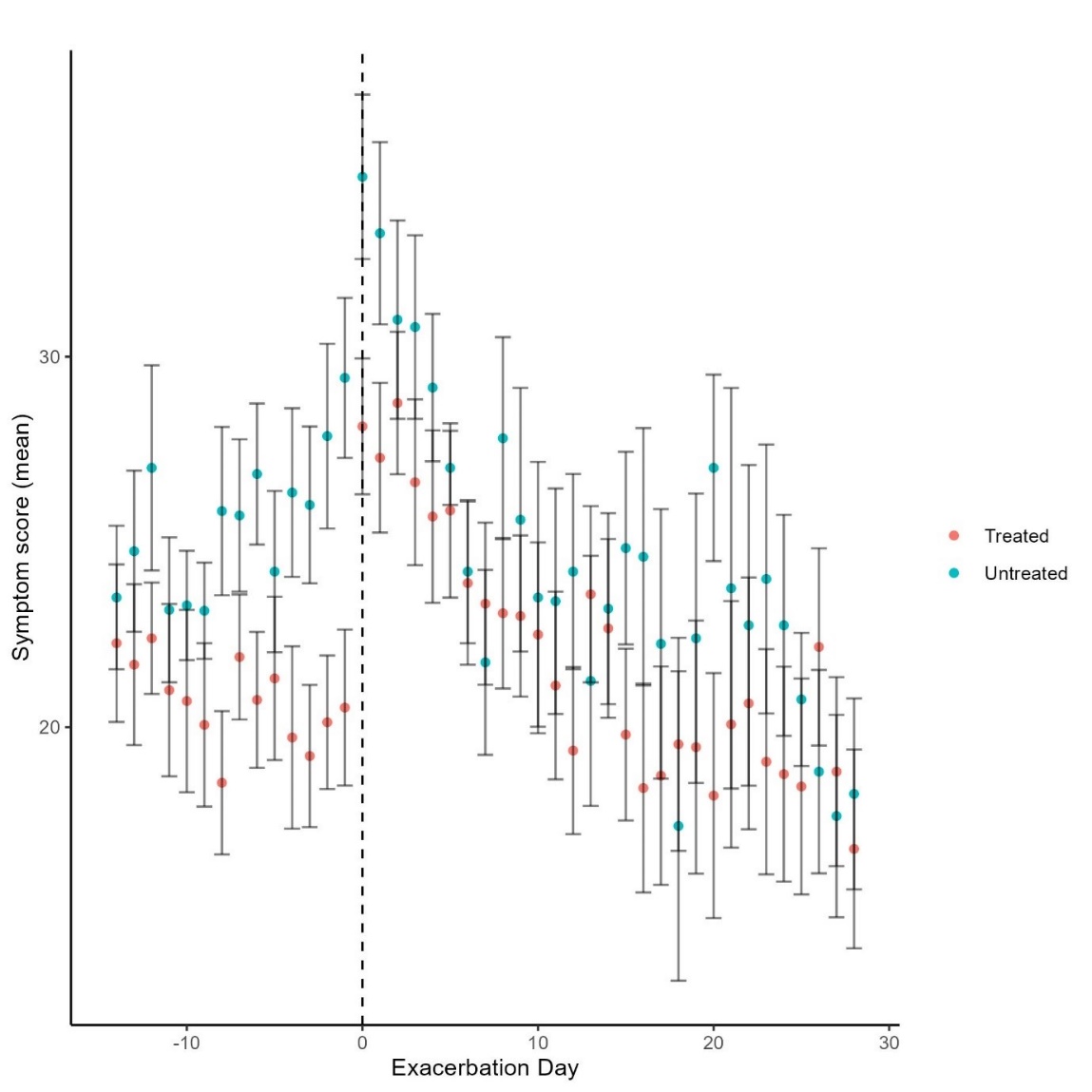


Figure S4: Mean symptom diary score across the time course of an exacerbation

Day 0 = onset of exacerbation. Error bars represent standard error of the mean.


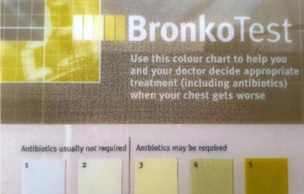


Figure S5: Bronkotest colour chart

|  | | **Colour Chart, SM Plan and Rescue Pack (intervention)** | **SM Plan and Rescue Pack**  **(best usual care)** | **Total** |
| --- | --- | --- | --- | --- |
|  |  | **N=57** | **N=58** | **N=115** |
| **Concomitant medication at baseline** | | | | |
| Nebulised drugs, n (%) | No | 42 (74%) | 46 (79%) | 88 (77%) |
|  | Yes | 15 (26%) | 12 (21%) | 27 (23%) |
| Among those who take Nebulised drugs, types of Nebulised drug, n (%) | | | | |
| Bronchodilators only |  | 10 (67%) | 8 (67%) | 18 (67%) |
| Saline only |  | 1 (7%) | 0 (0%) | 1 (4%) |
| Colomycin only |  | 0 (0%) | 1 (8%) | 1 (4%) |
| Unknown |  | 1 (7%) | 0 (0%) | 1 (4%) |
| Bronchodilators and Saline |  | 3 (20%) | 3 (25%) | 6 (22%) |
| Types of Inhaled drug, n (%) | No | 1 (2%) | 2 (3%) | 3 (3%) |
|  | Yes | 56 (98%) | 56 (97%) | 112 (97%) |
| Among those who take Inhaled drugs, types of Inhaled drug, n (%) | | | | |
| LAMA |  | 0 (0%) | 1 (2%) | 1 (1%) |
| LAMA/LABA |  | 7 (13%) | 4 (7%) | 11 (10%) |
| LABA/ICS |  | 6 (11%) | 4 (7%) | 10 (9%) |
| LAMA/LABA/ICS |  | 40 (73%) | 43 (77%) | 83 (75%) |
| SABA |  | 1 (2%) | 3 (5%) | 4 (4%) |
| ICS |  | 1 (2%) | 0 (0%) | 1 (1%) |
| LABA |  | 0 (0%) | 1 (2%) | 1 (1%) |
| Missing |  | 1 | 0 | 1 |
| Mucolytic, n (%) |  | 29 (51%) | 26 (45%) | 55 (48%) |
| Prophylactic Antibiotic, n (%) |  | 7 (13%) | 10 (18%) | 17 (15%) |
|  | Missing | 2 | 1 | 3 |
| Theophylline, n (%) |  | 6 (11%) | 7 (12%) | 13 (11%) |
| Long-Term Oxygen Therapy (LTOT), n (%) |  | 3 (5%) | 0 (0%) | 3 (3%) |
| Ambulatory Oxygen, n (%) |  | 5 (9%) | 4 (7%) | 9 (8%) |
| Domiciliary Non-Invasive Ventilation, n (%) |  | 5 (9%) | 1 (2%) | 6 (5%) |
| Doxycyline (content of rescue pack), n (%) |  | 21 (37%) | 27 (47%) | 48 (42%) |
| Amoxicillin (content of rescue pack), n (%) |  | 30 (53%) | 25 (43%) | 55 (48%) |
| Co-amoxiclav (content of rescue pack), n (%) |  | 2 (4%) | 6 (10%) | 8 (7%) |
| Ciprofloxacin (content of rescue pack), n (%) |  | 0 (0%) | 2 (3%) | 2 (2%) |
| Clarithromycin (content of rescue pack), n (%) |  | 3 (5%) | 2 (3%) | 5 (4%) |

Table S7: Concomitant medication summary at baseline

**References**

1. Jose NC, Langel K. ePRO vs paper. Applied clinical trials. 2010;19:1-5.

2. Anthonisen NR, Manfreda J, Warren CP, Hershfield ES, Harding GK, Nelson NA. Antibiotic therapy in exacerbations of chronic obstructive pulmonary disease. Ann Intern Med. 1987;106(2):196-204.

3. Mackay AJ, Donaldson GC, Patel AR, Singh R, Kowlessar B, Wedzicha JA. Detection and severity grading of COPD exacerbations using the exacerbations of chronic pulmonary disease tool (EXACT). Eur Respir J. 2014;43(3):735-44.

4. Ellis P, Parekh G, Duvoix A, Watson L, Sharp A, Mobeen F, et al. Characteristics of alpha-1 antitrypsin deficiency related lung disease exacerbations using a daily symptom diary and urinary biomarkers. PLoS One. 2024;19(2):e0297125.
