# Supplementary material for: Sputum colour charts to guide antibiotic self-treatment of acute exacerbation of chronic obstructive pulmonary disease: the Colour-COPD RCT
Source: BMJ Open Respir Res. 2025 Oct 10;12(1):e003615. doi: 10.1136/bmjresp-2025-003615 (PMC12517013; doi:10.1136/bmjresp-2025-003615)
Supplement: online supplemental file 4 [file bmjresp-12-1-s004.pdf]

TRIAL PROTOCOL

Colour COPD

Sputum colour charts to guide antibiotic self-treatment of acute exacerbation of COPD (Colour COPD)

A 2 arm, multi-centre, open label, parallel-group randomised designed trial investigating the use of Sputum Colour Charts to guide antibiotic self-treatment of acute exacerbation of COPD in patients with COPD – Colour COPD

This protocol has regard for the HRA guidance and is compliant with SPIRIT

**Version Number:**	7.0
**Version Date:**	16 March 2023

Protocol development

Protocol Amendments				
The following amendments and/or administrative changes have been made to this protocol since the implementation of the first approved version.				
**Amendment number**	**Date of amendment**	**Protocol version number**	**Type of amendment**	**Summary of amendment**
included as part of initial ethics re-submission	03/11/2020	2.0	Part of initial application	Remote delivery of study intervention added and changes made throughout to reflect this.
SA_1	19/01/2021	3.0	Substantial	Data at all follow ups pertaining to healthcare usage and medications will be self-reported by the participant. Trail and TSC members updated, ISRCTN reference number added, additional case report forms (CRFs) have been included, changes made to make it more clear what the source data is for all data types.
NSA_02	04/03/2021	4.0	Non-substantial	The person carrying out the telephone calls for non-completion of the e-diary has been changed to the Data Manager at BCTU. Additionally, the 50% non-completion level has been replaced with regularly failing to complete the e-diary.

SA_03	01/07/2021	5.0	Substantial	1) updated individual's titles as appropriate; 2) Data Monitoring Committee (DMC) and Data Monitoring and Ethics Committee (DMEC) had been used interchangeably. This has been standardised to DMC throughout.; 3) changes made to the process of approaching and screening potential participants for inclusion into the trial by the Research for the Future initiative (Greater Manchester only) as outlined in changes 1 and by supporting documents in change 2 .; 4) Changes made to the E-diary sub-study section to clarify which questions are additional to those found in the EXACT questionnaire; 5) Inclusion criteria changed to age 18 or above (previously stated greater than 18); 6) Stated the process for taking informed consent when appointments take place remotely; 7) Increased fidelity call time window to plus or minus 7 days on Trial Flowchart to allow more flexibility; 8) Updated Adverse Events reporting in line with study requirements and regulations; 9) Minimisation variables made more clear to show that only COPD GOLD categories C and D will be included and that hospitalisations are 1 or more (as per eligibility criteria); 10) corrected minor typographical/formatting errors.

SA_06	13/10/2022	6.0	Substantial	Updates made to DMC and TMG staff. Additional abbreviations added to list. Clinical aim and objective added. Approaching patients via SMS text messages about the trial is also added to the Protocol- standardised text for this has been submitted with this amendment. Acceptability of intervention updated to include multiple long term conditions (MLTCs) which includes barriers to participating in the trial for patients who have MLTCs and reasons for non-participation, including a short online/telephone questionnaire for those patients who are willing. Addition of PROMIS self-efficacy questionnaires included in acceptability of intervention section as well as in the 6 month follow up telephone call schedule of events. Exploratory analyses of PROMIS questionnaires added and appendices with screen shots of these questionnaires added. MLTC add-on funding has been approved and is described. Patients who decline to participate will be asked by the site if they are willing to discuss their reasons for declining to take part in the trial with a researcher.
SA_07	16/03/2023	7.0	Substantial	Wording added to recruitment and follow up telephone calls sections to clarify these will no longer take place as the funder has withdrawn funding and the trial is due to close.

Funding and Support in Kind	
**Funder (s)**<br>(Names and contact details of all organisations providing funding and/or support in kind for this trial)	**Financial and non-financial support given:**
National Institute of Health Research (NIHR)	Financial support to run the study
Funding Scheme (if applicable)	NIHR HTA
Funder's reference number	17/128/04
The funder of the trial has had no role in the trial design, data collection, data analysis or data interpretation.	

Protocol Sign Off

CI Signature Page

The undersigned confirm that the following protocol has been agreed and accepted and that the Chief Investigator agrees to conduct the trial in compliance with the approved protocol.

I agree to ensure that the information contained in this document will not be used for any other purpose other than the evaluation or conduct of the clinical investigation without the prior written consent of the Sponsor.

I also confirm that I will make the findings of the study publicly available through publication or other dissemination tools without any unnecessary delay and that an honest accurate and transparent account of the study will be given; and that any discrepancies from the study as planned in this protocol will be explained.

This protocol has been approved by:

Trial Name:	**Colour COPD**
Protocol Version Number:	Version: 7.0
Protocol Version Date:	30 March 2023
CI Name:	Professor Alice Turner
Trial Role:	Chief Investigator
Signature and date:	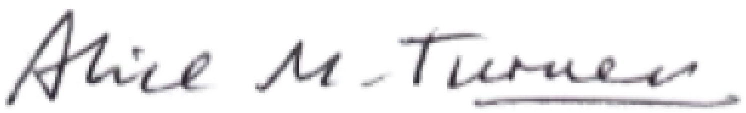 _ ______________________ __ 30__ / _03_ / 2023_ __ __
Team Leader Name:	Sarah Tearne
Signature and date:	______________________ __ __ / __ __ / __ __ __ __
Trial Statistician Name:	Rajnikant Mehta
Signature and date:	______________________ __ __ / __ __ / __ __ __ __

Sponsor statement:

Where the University of Birmingham takes on the sponsor role for protocol development oversight, the signing of the IRAS form by the sponsor will serve as confirmation of approval of this protocol.

Compliance statement:

This protocol describes the **Colour COPD** trial only. The protocol should not be used as a guide for the treatment of participants not taking part in the **Colour COPD** trial.

The study will be conducted in compliance with the approved protocol, UK Policy Framework for Health and Social Care Research 2017, the Data Protection Act 2018, and the principals of Good Clinical Practice as defined by the European Good Clinical Practice (GCP) Directive. Every care has been taken in the drafting of this protocol, but future amendments may be necessary, which will receive the required approvals prior to implementation.

PI Signature Page

The undersigned confirm that the following protocol has been agreed and accepted and that the Principal Investigator agrees to conduct the trial in compliance with the approved protocol.

I agree to ensure that the confidential information contained in this document will not be used for any other purpose other than the evaluation or conduct of the clinical investigation without the prior written consent of the Sponsor.

This protocol has been approved by:

Trial Name:	**Colour COPD**
Protocol Version Number:	Version 7.0
Protocol Version Date:	30 March 2023
PI Name:	
Name of Site:	
Signature and date:	___________________________ __ __ / __ __ / __ __ __ __

**Reference Numbers**	
EudraCT number	N/A
Sponsor number	RG_18-088
ISRCTN reference number	14955629
IRAS reference number	263204

Administrative Information

Sponsor	
University of Birmingham	The University of Birmingham<br>Edgbaston<br>Birmingham<br>B15 2TT
Contact Details:	Dr Birgit Whitman<br>Head of Research Governance and Integrity<br>Phone: 0121 415 8011<br>Email:<br>researchgovernance@contacts.bham.ac.uk

Chief Investigator	
Prof Alice Turner	Professor of Respiratory Medicine
University of Birmingham<br>Institute of Applied Health Research<br>Murray Learning Centre<br>Edgbaston<br>B15 2TT	+44 (0) 121 371 3885<br>a.m.turner@bham.ac.uk

Data Monitoring Committee – DMC
Chair: Professor Stephen Bourke – Consultant in respiratory medicine and honorary senior lecturer, Newcastle University<br><br>Independent Members<br>Dr Sarah Deacon - Consultant Physician in Respiratory and General Medicine, Worcestershire Royal Hospital<br><br>Dr Victoria Harris, Medical Statistician, University of Oxford, Clinical Trials Unit, Oxford.

Trial Steering Committee - TSC
Chair: Dr Linzy Houchen-Wolloff – Senior Research Associate, University Hospitals of Leicester NHS Trust<br><br>Independent Members

Dr Nick Hopkinson - Reader in Respiratory Medicine at Imperial College, Honorary Consultant Chest Physician at The Royal Brompton Hospital<br><br>Dr Dipak Kotecha - National Institute for Health Research (NIHR) Career Development Fellow, University of Birmingham, UK, Consultant Cardiologist, UHB and SWBH NHS Trusts, Birmingham, UK<br><br>Dr Tim Harries- School of Population Health and Environmental Sciences, King's College London, Addison House, Guy's Campus, SE1 1UL. General Practitioner and Honorary contract with Kings College London.<br><br>Mrs Sue Boex – PPI Representative

Trial Management Group - TMG

Prof Alice Turner	Chair
Mrs Sarah Tearne	Trial Management Team Leader
Mrs Sarah Moorlock	Senior Trial Manager
Mrs Reshma Ali	Senior Data Manager
Mrs Suzanne Johnston	Data Manager
Mr Rajnikant Mehta	Senior Trial Statistician
Mrs Eleni Gkini	Trial Statistician
Prof Sue Jowett	Economist
Prof Nicola Gale	Social Scientist
Dr Rachel Adams	Social Scientist
Prof Peymane Adab	Co-applicant
Dr Rachel Jordan	Co-applicant
Mr David Edwards	Analyst Programmer
Dr Naomi Campton	Senior Analyst Programmer
Prof Nawar Bakerly	Co-applicant, Northern Care Alliance

Trial Office Contact Details

Mrs Sarah Moorlock	Tel: 0121 414 3105<br>Email: S.J.Moorlock@bham.ac.uk
The University of Birmingham<br>Edgbaston<br>Birmingham<br>B15 2TT	Tel: 0121 414 3105<br>ColourCOPD@trials.bham.ac.uk
Randomisation website/ telephone number	https://bctu-redcap.bham.ac.uk/
Trial website	www.birmingham.ac.uk/ColourCOPD
Trial social media	https://twitter.com/ColourCOPD

ABBREVIATIONS

Abbreviation	Term
**ABPI**	Association of British Pharmaceutical Industry
**AECOPD**	Acute exacerbation of COPD
**BCTU**	Birmingham Clinical Trials Unit
**CCG**	Clinical Commissioning Group
**CI**	Chief Investigator
**COPD**	Chronic Obstructive Pulmonary Disease
**CPRD**	Clinical Practice Research Datalink
**CRF**	Case Report Form
**CRN**	Clinical Research Network
**DCF**	Data Clarification Form
**DMC**	Data Monitoring Committee
**EG**	For example
**EQ-5D-5L**	Euroqol questionnaire
**FEV**	Forced Expiratory Volume
**FVC**	Forced Vital Capacity
**GOLD**	Global Obstructive Lung Disease
**GCP**	Good Clinical Practice
**GP**	General Practitioner
**HEAP**	Health Economics Analysis Plan
**HES**	Hospital Episode Statistic
**HRA**	Health Research Authority
**HRU**	Health Resource Utilisation
**ICF**	Informed Consent Form
**ICH**	International Conference on Harmonisation
**ISF**	Investigator Site File
**MLTC**	Multiple long-term conditions
**NHS**	National Health Service
**PI**	Principal Investigator

**PIC**	Participant Identification Centres
**PIS**	Participant Information Sheet
**PPI**	Public and Participant Involvement
**PROMIS**	Patient-Reported Outcomes Measurement Information System.
**QA**	Quality Assurance
**QALY**	Quality Adjusted Life Years
**QMS**	Quality Management System
**RCT**	Randomised Controlled Trial
**REC**	Research Ethics Committee
**RGT**	Research Governance Team
**RP**	Rescue Pack
**RGT**	Research Governance Team
**SAE**	Serious Adverse Event
**SAP**	Statistical Analysis Plan
**SAR**	Serious Adverse Reaction
**SM**	Self-Management
**SGRQ**	St George's Respiratory Questionnaire
**SUSAR**	Suspected Unexpected Serious Adverse Reaction
**UoB**	University of Birmingham
**UK**	United Kingdom

DEFINITIONS

Term	Abbreviation	Description
Acute exacerbation of COPD	**AECOPD**	Worsening of respiratory symptoms for patients suffering with COPD, beyond normal day-to-day variations and leading to a change in medication.
Chronic Obstructive Pulmonary Disease	**COPD**	Common day to day symptoms include breathlessness, which is typically worse on exertion, and cough productive of sputum. COPD is defined by airflow

		obstruction on spirometry, this being a ratio less than 0.7 and lower than the lower limit of normal for age in the forced expiratory volume in 1 second (FEV_1) and forced vital capacity (FVC) after administration of a bronchodilator.
Treatment Failure		Ongoing symptoms and/or requirement for treatment in the 14 days after a self-managed event.
Unreported AECOPD		Daily symptom change in the absence of input from a healthcare professional, or reporting symptom change to a healthcare professional.
Chronic Bronchitis		Self-reported sputum production for at least 3 months in each of 2 consecutive years or more.

TRIAL SUMMARY

Title: Sputum colour charts to guide antibiotic self-treatment of acute exacerbation of COPD (Colour COPD)

The primary objective of this study is to determine if a sputum colour chart can aid patient self-management of COPD exacerbations, such that use of the chart is non-inferior to usual care with respect to hospital admissions. A range of other secondary objectives are described in the main protocol. An integral pilot phase is included and described in the main protocol. An economic evaluation and process evaluation are also included.

Trial Design: A 2 arm, multi-centre, open, non-inferiority parallel-group, individually randomised designed trial
Setting: recruiting from approximately 80 GP practices across England, predominantly from Birmingham and Greater Manchester areas

Participant Population and Sample Size: 2954 patients with COPD who have had ≥2 AECOPD or ≥1 hospital admission for AECOPD in the preceding year before screening

Eligibility Criteria:

Included patients will have:

- Clinically diagnosed COPD, confirmed by a medical record of post-bronchodilator spirometry denoting obstruction
- ≥2 AECOPD in the 12 months prior to screening according to the patient or ≥1 hospital admission for AECOPD (i.e. GOLD C or D; Figure 1)
- Able to safely use SM plan in the view of their usual care practitioner
- Able to use sputum colour chart; this will be confirmed by a sight test if there is any doubt on initial assessment by the usual care or research team. Patients who report being colour blind will have their ability to use the chart tested at the screening visit.
- Written Informed consent given
- Additionally, to participate in the E-diary sub-study
  - Access to smartphone/tablet and an email address
- Additionally, to participate in the Sputum sub-study
  - Chronic bronchitis, defined by self-reported sputum production for at least 3 months in each of 2 consecutive years or more.

Exclusion criteria will be:

- Household member already participating in the study

Interventions

Experimental Arm: use of the 5 point sputum colour chart, adapted from Bronkotest® a self-management (SM) plan and rescue pack (RP) containing 5 days of antibiotic and steroid treatment

Control Arm: use of the plan and pack alone (best usual care)

Outcome Measures

Primary Outcome:

Hospital admission rates at 12 months after enrolment where the primary reason for admission is AECOPD

Secondary Outcomes:

- Self-reported AECOPD every 3 months
- Self-reported antibiotic and steroid prescriptions for AECOPD
- All cause hospital admission
- Readmissions to hospital for AECOPD at 30 and 90 days
- Bed days due to AECOPD
- Mortality
- Unscheduled GP visits for AECOPD
- Self-reported prescriptions for 2nd courses of antibiotics within 14 days of self-reported event
- Self-reported prescriptions for oral anti-fungals
- Quality of life score (COPD assessment test [CAT], EQ-5D-5L) at 3 monthly intervals
- Antibiotic resistance
- Healthcare Resource Utilisation (HRU).

Further details on time points for each measure are shown in the outcomes section of the protocol.

Trial Schema

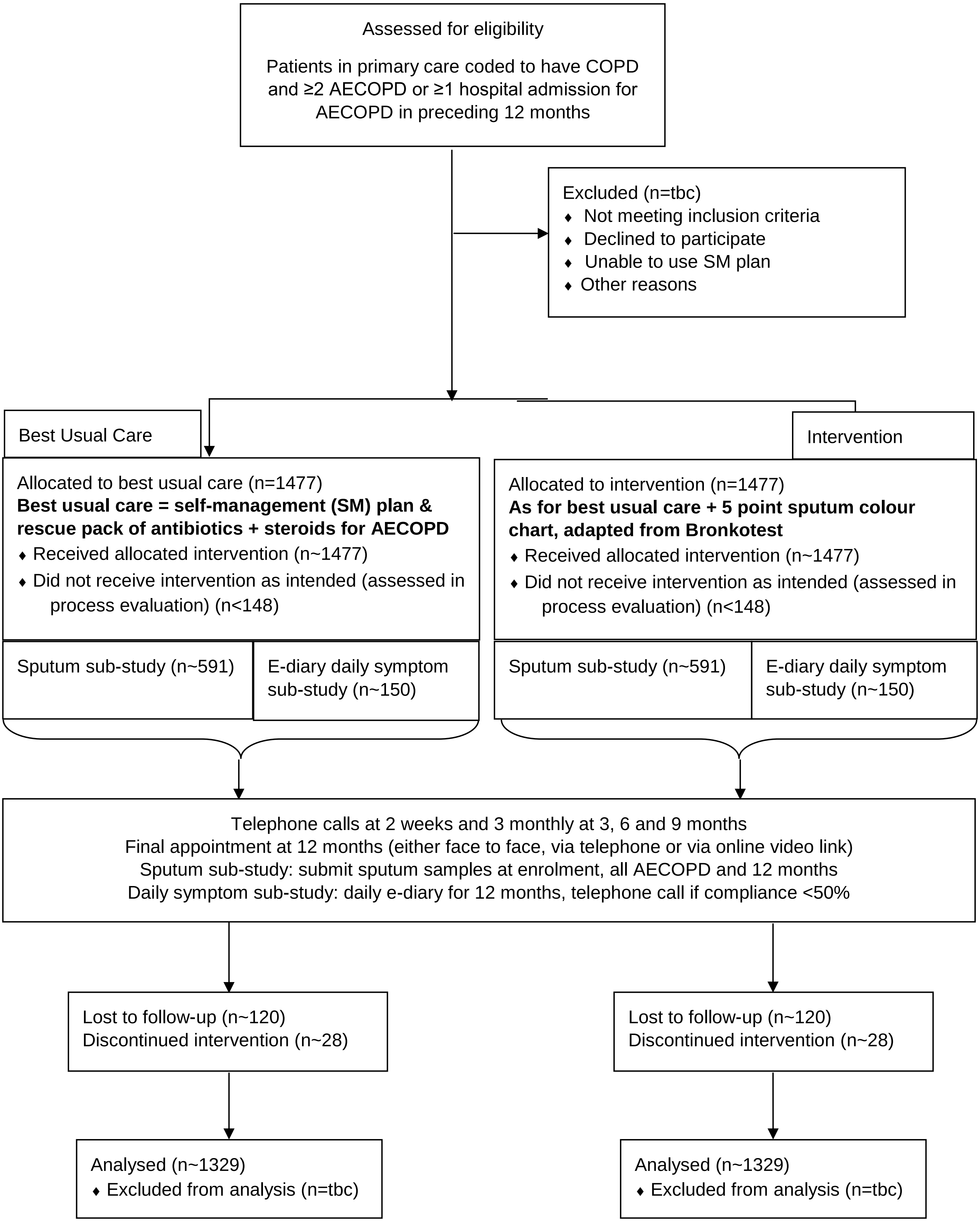

TABLE OF CONTENTS

1. BACKGROUND AND RATIONALE **18**
1.1 Background 18
1.2 Trial Rationale 19
1.2.1 Justification for participant population *20*
1.2.2 Justification for design *21*
1.2.3 Choice of intervention *22*
2. AIMS AND OBJECTIVES **23**
2.1. Pilot Stage Objectives 23
2.2. Main Trial Objectives 23
2.2.1 Clinical Aims and Objectives *23*
2.2.2 Economic Aims and Objectives *24*
3. TRIAL DESIGN AND SETTING **24**
3.1. Trial Design 24
3.2. Trial Setting 25
3.3. Identification of participants 25
3.4. Sub-studies 26
3.5. Assessment of Risk 28
4. ELIGIBILITY **28**
4.1. Included patients will have: 28
4.2. Exclusion criteria will be: 28
5. Co-enrolment **28**
6. CONSENT **29**
7. ENROLMENT AND RANDOMISATION **30**
7.1. Enrolment and Screening 30
7.2. Randomisation 30
7.2.1. Randomisation Methodology *30*
7.2.2. Blinding *30*
7.2.3. Randomisation Process *30*
7.2.4. Randomisation Records *31*
7.3. Informing Other Parties 31
8. TRIAL TREATMENT / INTERVENTION **31**
8.1. Intervention(s) and Schedule 31
8.2. Continuation after the Trial 31
9. OUTCOME MEASURES AND STUDY PROCEDURES **32**
9.1.2. Schedule of Assessments *32*
Trial Flowchart 35
9.2. Main Trial Outcomes 36

9.2.2. Primary Outcomes 36
9.2.3. Secondary Outcomes 36
9.3. Participant Withdrawal and Changes of Status Within Trial 37
10.3.1 Patient Mortality 38
ADVERSE EVENT REPORTING 39
10.0 Definitions 39
10.1 Adverse Event General Recording Requirements 39
10.2 Adverse Events Reporting Requirements in Colour COPD 40
10.3 Serious Adverse Advents (SAE) Reporting in Colour COPD 40
10.3.1 Events not requiring reporting to BCTU on an SAE form 41
10.4 Reporting procedure 41
10.4.1 Reporting procedure for Serious Adverse Events by sites 41
10.4.2 Provision of follow-up information 42
10.5 Assessment of relatedness 42
10.6 Assessment of Expectedness by the CI 43
10.6.1 Protocol defined expected SAEs 43
10.7 Reporting SAEs to third parties 43
10.8 Urgent Safety Measures 44
10.9 Monitoring pregnancies for potential Serious Adverse Events 44
11.0 DATA HANDLING AND RECORD KEEPING 44
11.1 Source Data 44
11.2 Case Report Form (CRF) Completion 45
11.3 Participant completed Questionnaires 47
11.4 Data Management 47
11.5 Data Security 48
11.6 Archiving 49
12.0 QUALITY CONTROL AND QUALITY ASSURANCE 49
12.1 Site Set-up and Initiation 49
12.2 Monitoring 49
12.3 Onsite Monitoring 49
12.4 Central Monitoring 49
12.5 Audit and Inspection 50
12.6 Notification of Serious Breaches 50
13.0 END OF TRIAL DEFINITION 50
14.0 STATISTICAL CONSIDERATIONS 51
14.1 Sample Size 51
14.2 Analysis of Outcome Measures 51
14.2.1 Primary Outcome Measure 52
14.2.2 Secondary Outcome Measures 52
14.2.3 Subgroup Analyses 54
14.2.4 Missing Data and Sensitivity Analyses 54

14.2.5 Exploratory Analyses 54
14.3 Planned Interim Analysis 54
14.4 Planned Final Analyses 54
15.0 Health Economic Evaluation 55
16.0 TRIAL ORGANISATIONAL STRUCTURE 56
16.1 Sponsor 56
16.2 Coordinating Centre 56
16.3 Trial Management Group 56
16.4 Trial Steering Committee 56
16.5 Data Monitoring Committee 56
16.6 Finance 57
17.0 ETHICAL CONSIDERATIONS 57
18.0 CONFIDENTIALITY AND DATA PROTECTION 57
19.0 FINANCIAL AND OTHER COMPETING INTERESTS 58
20.0 INSURANCE AND INDEMNITY 58
21.0 POST-TRIAL CARE 58
22.0 PUBLICATION POLICY 59
23.0 ACCESS TO FINAL DATA SET 59
24.0 REFERENCE LIST 60
25.0 APPENDICES 64

1. BACKGROUND AND RATIONALE

1.1 Background

<u>What is the problem being addressed?</u>
chronic obstructive pulmonary disease (COPD) is a chronic condition affecting 2 million people in the UK (2), causing over 140,000 hospital admissions and 1.7% of UK hospital bed days per year (2). Common day to day symptoms include breathlessness, which is typically worse on exertion, and cough productive of sputum. COPD is defined by airflow obstruction on spirometry, this being a ratio less than 0.7 and lower than the lower limit of normal for age in the forced expiratory volume in 1 second (FEV_1) and forced vital capacity after administration of a bronchodilator.
COPD severity is determined by the degree of FEV_1 impairment relative to a normal individual of the same age, sex and height, expressed as the % predicted for age (3). A variety of studies have derived these normal values and this study will use global lung function initiative equations (5). It is also possible to define severity as described by the Global Obstructive Lung Disease outcomes strategy (7), and illustrated in Figure 1 in this system category C and D patients have ≥2 exacerbations/year or ≥1 which results in hospital admission, and are distinguished by their degree of breathlessness (MRC score) or quality of life (CAT score). Patients in category D have a high symptom burden, as well as exacerbations, whereas category C patients have fewer day to day symptoms. This grading system allows greater personalization of daily therapy, based on exacerbation rate (7). People who have had exacerbations before are those more likely to have them again, thus are an important group to target when educating patients to self-manage such events.

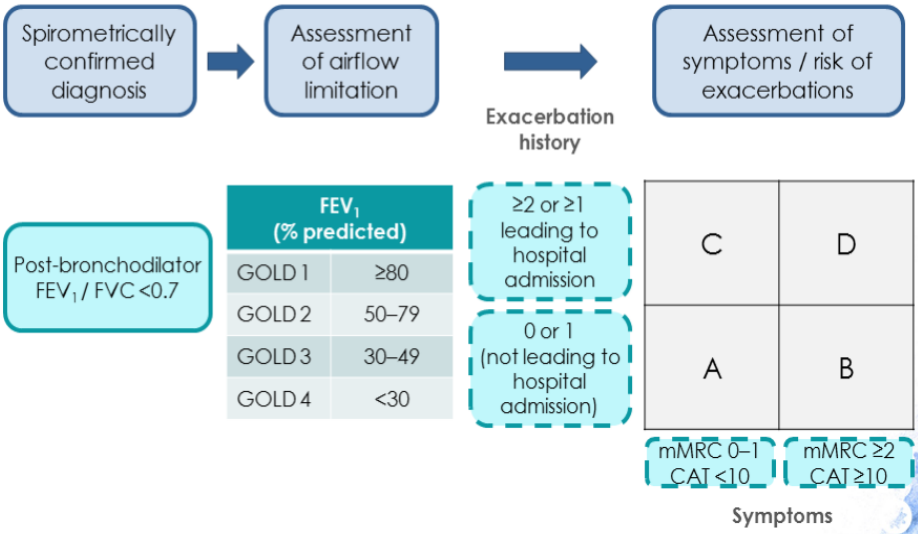

<u>Figure 1: Severity of COPD according to Global Obstructive Lung Disease outcomes strategy</u>

Most admissions to hospital in COPD patients are for exacerbations of COPD (AECOPD). Exacerbations are defined by 'worsening of respiratory symptoms beyond normal day-to-day variations and leading to a change in medication' (7). Cardinal symptoms include altered sputum volume and/or colour and worsening dyspnoea (8). Exacerbation pathophysiology is not fully understood, but includes elements of infection and of environmental triggers (9, 10). A systematic review in 2012 found bacteria in just 46% of events (11), suggesting antibiotics will effectively manage only half of AECOPD episodes - nevertheless they are used in the

majority of events. Sub-types of exacerbation can be classified according to symptoms (type 1 = increased volume and purulence of sputum and worsening breathlessness, type 2 = 2 out of 3 of these symptoms and type 3 = worsening breathlessness with no other symptom (8)) or health care use (mild = increased inhaler use alone, moderate = primary care visit, antibiotic or steroid use, severe = hospitalization, or unreported (12). Data from trials and cohort studies suggest that 50-70% of exacerbations identified through daily diary reporting are unreported and there is evidence that patients with unreported exacerbations have poorer prognosis with faster lung function and health status decline compared with those who have no unreported events (12-15). Increasing AECOPD frequency generally leads to faster disease progression(16), and hospitalization for AECOPD predicts mortality (17-19). The evidence on whether prognosis differs according to the underlying cause of AECOPD (bacterial, viral or non-infective) is inconsistent (20). Therefore it is important for trials where AECOPD are an outcome to capture overall AECOPD rate, including hospitalized and unreported events.

One approach to reduce the impact of exacerbations is the use of self-management (SM) plans, alongside a pack of antibiotics and steroids (rescue pack)(3). Evidence for SM as a means of reducing admission in COPD is inconsistent (21, 22) and heterogeneous (23). Multidisciplinary SM support programmes including action plans for AECOPD management are effective in reducing admissions when they include iterative feedback to patients (24). A systematic review conducted by our group attempted to delineate the effect of each component of SM, of which simple action plans for AECOPD management are one; we did not find an effect of any one component on outcomes, including hospital admissions and AECOPD rates (23, 25).

In the UK patients are usually given an action plan alongside a pack and advised to use both antibiotic and steroid components together for all AECOPD. Iterative feedback is not usually provided and instead most use a traffic light system to determine whether medical help should be sought or treatment taken. Furthermore, UK health professionals have identified numerous training needs to deliver iterative SM (26). Based on the evidence from trials, SM plans as currently used in usual care in the UK (i.e. simple action plans with little ongoing support), are unlikely to reduce hospitalisations. This evidence that SM plans as used in usual NHS care do not alter hospital admission rates is the primary reason for choosing a non-inferiority design in this trial, rather than hypothesizing that a colour chart within a SM plan could influence admissions either by increasing or decreasing them.

1.2 **Trial Rationale**

Why is this research important?

This study addresses the problem of personalising and optimising effectiveness of AECOPD management. It is now widely accepted that each year around half of all patients with COPD have frequent AECOPD (≥2 per year (27)), and in the UK 44-85% of patients with this AECOPD rate were hospitalised again within 12 months (28). AECOPD admissions drive the economic burden of COPD, estimated at £1.8bn in direct costs alone (29). Rapid readmissions are also common – the national COPD audit has shown that 43% of patients with COPD who were admitted are back in hospital within 90 days (30), and up to 71% by 12 months(31). Intuitively early recognition and treatment of AECOPD would reduce exacerbation severity and duration, and improve prognosis; evidence for this is limited but supportive (32).

Usual care for COPD patients in the UK consists of agreeing a SM plan which includes provision of a "rescue pack" of both antibiotics and steroids, which patients are advised to take when they suspect they have an exacerbation. However, only half (11) of all exacerbations are

bacterial and would require treatment with antibiotics, suggesting that overuse of antibiotics occurs. Inappropriate use or overuse increases the long-term risk of antibiotic resistance (33), and reducing antibiotic resistance through appropriate stewardship is a recognised NHS priority (34). In hospitalised AECOPD patients resistance occurs in up to 66% of cases, and relates to past antibiotic use (35), suggesting those with prior hospitalisation or frequent antibiotic courses are a key group to target for interventions aimed at reducing resistance.

Sputum colour is a marker of neutrophilic inflammation and bacterial infection (36), suggesting it could be used to guide antibiotic treatment and reduce inappropriate use. In studies conducted in Birmingham in approximately 100 patients over a year (36) there was 94% probability that infectious exacerbations of COPD had green sputum (sensitivity of green sputum = 94%). Specificity of green sputum for bacterial infection was 77%. This suggests sputum colour is a tool with potential to reduce inappropriate antibiotic use, and a definitive study to test this is required.

<u>Why this research is needed now</u>

The evidence presented above demonstrates that use of a sputum colour chart could be a simple way to guide antibiotic treatment and enhance SM by COPD patients. However, given that the intervention could reduce antibiotic use in some patients and deviate from current routine practice, it is important to assess its safety as well as the potential to reduce future antibiotic resistance problems. Since previously hospitalized COPD patients have high rates of resistance already (35) they represent an ideal population in whom to maximize power to detect effects on bacterial resistance in a trial. In addition they are a particularly important group for study since hospitalization rates are rising (37). An adequately powered non-inferiority study will evaluate whether a colour chart based strategy is safe and does not adversely impact on hospitalization compared to usual care. The trial will also be able to test whether personalization of treatment using a colour chart, compared to best usual care, impacts on other important outcomes for both personal and public health, including QOL and antibiotic resistance rates.

1.2.1 Justification for participant population

The patient population will be drawn from UK primary care. We have chosen a patient population who we can be confident have COPD by including those who have a previous spirometry indicative of COPD, and who are at risk of hospital admissions by including a prior history of frequent exacerbations. Any past post-bronchodilator spirometry confirming airflow obstruction will be sufficient to determine eligibility, since a recent FEV_1 is not required to determine COPD severity in the current GOLD model (Figure 1). In practice this means we will screen patients coded to have COPD, and record their spirometry from the record at the time of the first visit (or telephone/video link appointment). Spirometry may be entered to the GP record in a variety of places which makes searching for this centrally prior to inviting patients potentially impractical.

We recognize that keeping inclusion as broad as possible, in order to ensure results are likely to be generalisable to the whole COPD population in Britain, is desirable and for this reason have not included any other severity features (e.g. % predicted FEV_1) or prior treatment expectations. In addition, patients with co-existent asthma or bronchiectasis will be eligible to participate providing these conditions are not deemed in the opinion of the treating physician to be the main condition(s), although this will be recorded and explored within the data to examine if sub-group effects are present. Such individuals would commonly be excluded from

drug trials, but constitute a large proportion of the COPD population in primary care; 22% of COPD patients are also coded to have asthma (38) and 29% are found to have bronchiectasis on CT scan (39). Including such patients keeps our results generalisable.

It was also necessary to choose a population who would enable adequate power in the study. We have selected a population at risk of admissions, as delineated by data from UK primary care. The clinical practice research datalink (CPRD) has been linked to hospital episode statistics (HES) for 44201 patients and the annual AECOPD rate per person per year assessed according to COPD severity(28). In GOLD C patients AECOPD occurred at a rate of 1.78 and in GOLD D 2.51 per person per year, with a COPD specific hospitalization rate of 0.44 and 0.85 per person per year respectively in the most recent year for which data was available. AECOPD rates were lower in GOLD A and B patients, and hospital admissions markedly lower in GOLD A patients. We have therefore selected patients in GOLD C and D severities (Figure 1) as our eligible population, and these published rates have informed our power calculations. CPRD data also shows that these GOLD C and D patients comprise nearly 46% of registered COPD patients in primary care, indicating that this should not limit recruitment. Changing definitions of COPD severity within GOLD, specifically the removal of FEV_1 % predicted as a severity criterion, has potential to alter this prevalence(40), however in UK data the proportions do not appear to change markedly when using AECOPD rate instead of spirometry to determine severity (41). We also consulted the national COPD audit to verify data on admission rates in the period after a hospitalization(37); the most recent report includes data from 183 hospitals and 13414 patients in England and Wales, and the rates indicated a similar risk to the CPRD data such that we were confident in the veracity of the data with respect to the whole UK.

1.2.2 Justification for design

This study is a pragmatic, individually randomized trial, set in primary care, comparing usual care to the use of a sputum colour chart in patients at risk of hospital admission for AECOPD, with the hypothesis that use of a colour chart will be non-inferior to usual care with respect to hospital admission rate after 12 months of follow-up, this being our primary outcome measure. Use of a chart might reduce antibiotic use, and thus might also change patterns of antibiotic resistance long term, such that these are important secondary outcomes. There is an internal pilot phase, a detailed process evaluation and a cost-effectiveness study. We have also included two sub-studies, one using a daily electronic symptom diary capable of picking up symptom defined (but potentially unreported) AECOPD, since these are an important prognostic marker (section 3), and one collecting sputum from patients to assess patterns of antibiotic resistance.

The option of superiority or non-inferiority was fully reviewed at the funding application stage, and the non-inferiority choice was recognised as appropriate by the funder and reviewers. Our non-inferiority design looks primarily at whether the addition of the sputum colour chart is safe (i.e. there is no increase in hospital admissions over 12 months relative to best usual care). We have powered using hospitalisations as the primary outcome and chosen as small a non-inferiority threshold as feasible so that we can be sure safety is robustly examined. Within our secondary outcomes we will assess whether the intervention is superior to best usual care in terms of reducing inappropriate antibiotic use, however as this is a secondary outcome it would be inappropriate to power on this a priori. We have added work packages on acceptability of the intervention and on health economics in order to understand how it might be used practically within the UK health economy if the trial demonstrated it was clinically effective.

1.2.3 Choice of intervention

5 point sputum colour chart, adapted from Bronkotest®, alongside a standardised self-management (SM) plan and rescue pack

This trial addresses an NIHR commissioned call which requested use of a sputum colour chart as part of a self-management intervention that enables patients with COPD to determine whether they have an exacerbation, and whether this requires antibiotic treatment. Several sputum charts are available. We have chosen the 5 point sputum colour chart, adapted from Bronkotest® from amongst these because:

1. The 5 point sputum colour chart, adapted from Bronkotest® is the only one that has been validated for use in COPD
2. It has been validated against sputum bacterial load - 84% of purulent samples (darker green colour) contained bacteria compared to 38% of mucoid (lighter colour) samples (36).
3. The 5 point sputum colour chart, adapted from Bronkotest® is commercially available and inexpensive (<£2 per patient)
4. It was practical to use in UK primary care by patients to guide therapy (36, 42); patients using the chart to guide antibiotic use rarely experienced treatment failure(42)
5. Bronkotest® resources are available to train healthcare providers on its use; this will facilitate smooth study set up, and will be used at study commencement and site initiations. In short patients will be advised only to use the antibiotic component of their pack if their sputum is green, or significantly changed from their baseline colour. All patients will be advised to use oral steroids from their pack if they have symptoms consistent with a non-infective AECOPD (sputum not green).

Other available colour charts have a lower degree of validation, or are less practical to use, as described in the commissioning brief. For example the colour chart developed by Allegra *et al* was found to be too complex in clinical practice, and there was insufficient agreement between physicians' and patients' colour ratings (43). Another chart designed primarily for nasal mucus, with the purpose of enhancing antibiotic stewardship has no published validation studies supporting its use(44).

The ***comparator is best usual care for COPD***. In primary care this currently consists of a simple, written SM plan and rescue pack of 5 days antibiotic and steroid treatment with the duration of treatment being based on evidence from systematic reviews (45). However we are mindful that whilst this is recommended usual care, there is variation in how it is implemented in practice. For example, analysis of our data from the Birmingham COPD cohort study including around 1000 patients from primary care showed that fewer than half (45%) had an agreed SM plan at recruitment in 2012. Since then a local pay for performance incentive is likely to have increased rates, but variation remains likely (46). In order to standardize the SM component of treatment for patients in both arms, we have adapted the current NHS Salford SM plan as a template. We have chosen this plan because the pages on AECOPD management are derived from a nationally (British Lung Foundation) endorsed resource (47), are simple to read and are already in use in one of the two major areas we plan to recruit from. Patients in the usual care group will be advised to treat any AECOPD with both rescue pack elements, whereas intervention group patients will use their chart to guide antibiotic use.

2. AIMS AND OBJECTIVES

The main aim is to answer the research question: What is the effectiveness of sputum colour charts alongside a SM plan to guide antibiotic self-treatment by patients with AECOPD?

2.1. Pilot Stage Objectives

There is a 9 month internal pilot among 50% of practices and 15% of participants (n=444).

The internal pilot has the following purposes.

(i) To assess recruitment rate and exclusions. We aim for 50% of sites enrolling at 9 months from study approval by the relevant authorities, and target an overall recruitment rate of 6 patients per site per month. This target is an average across all sites, not per site and informs progression.

(ii) To assess spirometry in the GP record, specifically the rate of recording of raw values (which are required alongside age and sex to calculate predicted values), its ease of remote electronic extraction and any inconsistency between coded diagnosis of COPD and spirometry values indicative of airflow obstruction.

(iii) To determine practicality of hospital episode statistic HES data. We will do this by reviewing incidence of hospitalisations for AECOPD and AECOPD rate (including unreported events in the e-diary study) in the HES record and compare to self-reported events by the patient. Timeliness of obtaining data and degree of alignment to patient reported events will be used to determine if HES should continue to be used.

2.2. Main Trial Objectives

2.2.1 Clinical Aims and Objectives

1. The primary objective is to assess whether use of the 5 point sputum colour chart, alongside a SM plan and rescue pack containing 5 days of antibiotic and steroid treatment (the intervention) is safe, as defined by being not substantially worse compared to use of the plan and pack alone (best usual care) for rate of hospital admission for AECOPD at 12 months post enrolment (defined by randomisation time point).

2. Assess whether the intervention is safe, as defined by the rate of 30 and 90 day AECOPD readmissions, rate of treatment failure (defined by ongoing symptoms and/or requirement for treatment in the 14 days after a self-managed event), and time to next AECOPD after a self-managed event.

3. Determine whether use of the intervention is effective at 12 months after enrolment in terms of reducing self-reported antibiotic use when compared to best usual care, including rescue pack, as well as reducing adverse events related to antibiotics (e.g. oral thrush).

4. Describe the effect of the intervention on number of unscheduled GP attendances, for AECOPD, in the 12 months post enrolment.

5. Describe the effect of the intervention on unreported AECOPD rate through a sub-study using daily symptom diaries. Unreported AECOPD are defined by daily symptom change in the absence of input from a healthcare professional, or reporting symptom change to a healthcare professional. This sub-study will allow us to determine if unreported AECOPD are impacted upon by the intervention, and their rate in UK primary care; it also allows us to assess adherence to the intervention's advice accurately.

6. Describe the effect of the intervention on antibiotic resistance patterns in sputum of people with COPD. This sub-study also allows us to assess the appropriateness of antibiotic use by objectively confirming sputum colour at AECOPD, and confirming presence of bacteria.

7. To assess fidelity of delivery of the intervention by use of a checklist inquiring on critical features of education around colour chart and self-management plan use.

8. To assess adherence to SM plan advice by comparing use of AECOPD treatment to daily symptoms (e-diary subgroup only).

9. To explore patient self-efficacy using two short PROMIS (Patient Reported Outcomes Measurement Information System) questionnaires recorded at 6 months.

10. To explore social acceptability and practical responses to the intervention by interviewing both staff delivering the intervention and participants receiving the intervention.

2.2.2 Economic Aims and Objectives

1. Determine the cost-effectiveness and cost-utility of using a colour chart as part of a SM plan.

3. TRIAL DESIGN AND SETTING

3.1. Trial Design

Colour COPD is a 2 arm, multi-centre, open, parallel-group non-inferiority randomised controlled trial investigating the use of Sputum Colour Charts to guide antibiotic self-treatment of acute exacerbation of COPD in patients with COPD. There is an integral pilot,

sub-studies of acceptability of the intervention, antibiotic resistance patterns in sputum, daily symptom control and an economic analysis.

3.2. Trial Setting

The study is set in primary care, enrolling from GP practices predominantly within Birmingham and Greater Manchester areas. The initial approach to patients will be facilitated by the clinical research network (CRN), in conjunction with GP practices.

3.3. Identification of participants

The patient population will be drawn from UK primary care, enrolling from approximately 80 GP practices predominantly within Birmingham and Greater Manchester areas. Patients may also be identified from secondary care and study appointments may take place in Community Clinics within a primary care setting.

In the Greater Manchester region only, Salford Royal NHS Foundation Trust may also recruit patients via radio advertising by inviting people to register their interest in taking part in Respiratory research studies on the Research for the Future (RFTF) volunteer database, an NIHR Clinical Research Network Greater Manchester (NIHR CRN GM) initiative (https://www.researchforthefuture.org/). Potentially eligible participants, who identify as having COPD, will be sent the RFTF welcome information via post or email and a volunteer invite. Acceptance of this invite will signpost people to a named RFTF contact person. A RFTF team member will complete an initial screening via telephone to find out whether interested people may be eligible for inclusion in the trial. Potentially eligible participants, recruited through the radio advert or from the existing cohort of registered volunteers on the RFTF database, will then be sent a Participant Information Sheet (PIS) via post or email to give further information about the trial. Once the patient has been given at least as much time as they need to consider the trial, a screening appointment will be scheduled which will take place either at their usual GP practice, or a clinic setting in Secondary care or remotely via telephone/online video link. This appointment will be conducted by a healthcare professional who will take informed consent and confirm eligibility.

Patients coded in the GP record as having COPD will be identified by pre-screening performed by their usual medical and nursing care team in conjunction with the CRN to aid assessment of eligibility. Participant Identification Centres (PIC) sites may be used where appropriate.
Potentially eligible patients will be contacted by their usual care team and a Participant Information Sheet (PIS) will be sent out to them by post or email to give further information about the trial. Once adequate time has been allowed to consider the study a screening appointment will be scheduled which will take place either at their usual GP practice, over the telephone or via an online video link.

Patients may be contacted by SMS text message via their GP Practice or secondary care provider. This could be as a follow up to the participant invite letter (sent in the post) or as an initial recruitment approach. Patients will be asked to contact their usual health care provider if they have any questions or are interested in taking part in the trial (standardised text to be used for text messages is provided elsewhere). Where it is an initial recruitment approach, patients will be provided with the PIS before the usual consent procedure is followed.

Prior to the screening appointment, if the patient has expressed an interest in participating in the sputum sub-study, they will be asked to collect a sputum collection pot from their local practice and bring a sputum sample with them to the screening visit (if feasible and they are attending the Practice for a face-to-face visit). Although sputum collection is part of best standard practice for a COPD review, we recognise it may not be done universally. Consent for this element only (baseline sputum collection for the sputum sub-study) will initially be verbal, and documented by the practice team, before written informed consent is then taken at the screening visit itself or formally via telephone or online video link. This will allow the patient to produce their sputum sample at their convenience (e.g. in the morning when this may be easier) and in the comfort of their own home. If the patient is unable to produce sputum on that day or does not bring in a sample, they will have the option to do this at their first convenience and post the sample directly to the lab. If a patient brings a sample to the screening visit but does not enter either the sputum sub-study or the main trial, the patient and GP will have the option for this sample to be used clinically, or for it to be discarded; it will not be sent to the lab or used in trial analysis.

The screening appointment will be similar to the usual annual COPD review, thus aligns well to usual care; informed consent will be obtained prior to fully assessing the inclusion/exclusion criteria and delivering the intervention.

Recruitment to the study has now closed due to the withdrawal of funding by the NIHR.

3.4. Sub-studies

E-diary sub-study for unreported AECOPD: Exacerbations are most accurately identified in "real-time" using a symptom diary. A sub-study will compare self-reported AECOPD in "real-time" between the two study arms using an e-diary among patients who have suitable devices and agree to take part.

Whilst daily diaries can be done on paper they are most reliable and cost-effective when done electronically (50). We will assess e-diary AECOPD using the EXACT score, symptoms and Anthonisen types of AECOPD (8). The EXACT score reliably detects AECOPD (51). E-diary data will therefore describe unreported AECOPD rates, and potentially enhance power to detect AECOPD rate if these events are numerous. Furthermore it will provide additional data on patient behaviour with respect to antibiotic use in relation to daily symptoms (for example whether they are taken in a timely manner relative to symptom changes). The EXACT score does not ask about change in sputum colour, we will therefore ask this as an additional question, (separate to the EXACT 14-item questionnaire so scoring is unaffected). Intervention participants will be asked on the e-diary if they have noticed a change in their sputum colour and what number on their sputum chart represents their sputum colour. They will receive a colour chart to use alongside this question; it will be provided on a card (as for the whole intervention group) and the colours numbered to ensure that screen settings do not affect interpretation of colour by the patient.

E-diary users will be enrolled consecutively from the start, randomising as usual, aiming for n=300 (10% of the total). Although using patients' own devices reduces costs, it may mean the e-diary and main trial population differ. Analysis of the main trial and sub-studies is described in the statistical analysis plan

Sputum sub-study for antibiotic resistance: Antibiotic resistance in potentially pathogenic organisms will be compared between the two trial arms, using collected sputum

samples, the trial data, and data extracted from the patient's primary care records. About 40% of COPD patients produce sputum when stable (52), hence antibiotic resistance testing will be limited to these people. Sputum will be posted by patients using pre-paid next day delivery to the study team at enrolment, during all AECOPD and at 12 months after enrolment, processed as described in our previous work (53). We have established that postal delivery does not affect test accuracy in this population (54). If 70% of our sputum producers comply we will have 2481 sputum samples, half in each of intervention and control groups, thus ample data to describe patterns of bacteriology and any changes in antibiotic resistance patterns. We ascertained in 2 pre-application PPI meetings that posting sputum was acceptable to patients.

The analyses planned within the e-diary and sputum sub-studies are described in the statistical analysis plan.

Acceptability of intervention: To explore social acceptability and practical responses to the intervention (55), we will conduct qualitative interviews with a range of clinical and non-clinical staff as required to reach saturation to understand attitudes to the intervention and the overall goal of reducing unnecessary antibiotic use. We will also explore whether or not patients with multiple long-term conditions (MLTCs) experience SM differently. The analyses planned for this are outlined in the Statistical Analysis Plan (SAP). In addition, we will interview ~50 patients after enrolment, with follow-up interviews a few months later, splitting the sample equally between intervention and control groups and ensuring diversity in the sample, we will also purposively sample patients with a range of MLTCs. The topic guide will be developed drawing on existing literature and theories on attitudes to and practices around antibiotic prescription and use, symptom interpretation, confidence in SM of COPD and use of healthcare providers during exacerbation events, and in conjunction with our PPI group. Prior to being interviewed patients will be asked to complete both the Patient Reported Outcomes Measurement Information System (PROMIS) "General Self-Efficacy – Short Form 4a" and "Self-Efficacy for Managing Chronic Conditions - Managing Medications and Treatment – Short Form 8a" (see appendices). Patients will only be asked to do this once with the qualitative team.

We will also explore barriers to participation in trials for those with MLTCs. All those declining to participate in the trial will be invited to complete a very brief questionnaire online or over the phone. The usual healthcare provider will ask patients who decline to participate in the trial if they would be happy to answer some questions about their decision with a member of the research team. Patients who agree to this will be provided with a contact telephone number to call the researcher so they can self-identify. Before the questionnaire is completed, patients will be informed that it is a one-off phone call/online survey and that they are free to discontinue the survey at any point without their care being affected. Consent will be implicit by virtue of the fact that a) they have chosen to contact us and b) that they choose to answer the questions. Responses provided by the patient will be recorded on the decliners' questionnaire (online or via telephone call). The survey will be hosted by an approved University of Birmingham survey platform (JISC Online Survey Tool available at: https://www.onlinesurveys.ac.uk/). The researcher will not have access to any identifiable data from those completing the questionnaire. Data will be stored securely on the JISC Online Survey until data collection is complete, at which point it will be downloaded for analysis then deleted from the survey platform. Interviews will be audio recorded and transcribed verbatim, prior to qualitative analysis using the Framework method, as described in our previous work (56), which is a systematic approach well suited to interdisciplinary health research and to working with clinical and lay collaborators. The average self-efficacy scores for the

interviewees will be presented descriptively together with that of the average PROMIS self-efficacy scores of the trial participants at 6 months.

Some members of our PPI group will additionally be actively involved as co-researchers in the analysis stage of the research, through a series of data workshops, to help ensure that the patient voice remains central to this part of the work. The research with staff will also refine the longer term outcome measures from the usual care record, as we recognise a smaller dataset will be more practical in the period beyond the main study.

Recruitment to the sub studies has now closed due to the withdrawal of funding by the NIHR.

3.5. Assessment of Risk

All clinical trials can be considered to involve an element of risk and, in accordance with trials unit operating procedures this trial has been risk assessed, to clarify any risks relating uniquely to this trial. This risk assessment concluded that the risks of the study are low, since the intervention is likely to enhance the appropriateness of strategies patients choose to self-manage AECOPD.

4. ELIGIBILITY

4.1. Included patients will have:

- Clinically diagnosed COPD, confirmed by a medical record of post-bronchodilator spirometry denoting obstruction.
- ≥2 AECOPD in the previous 12 months according to the patient or ≥1 hospital admission for AECOPD (i.e. GOLD C or D; Figure 1).
- Able to safely use SM plan in the view of their usual care practitioner.
- Able to use sputum colour chart; this will be confirmed by a sight test if there is any doubt on initial assessment by the usual care or research team. Patients who report being colour blind will have their ability to use the chart tested at the screening appointment
- Age ≥18, with no upper limit.
- Informed consent given.
- Additionally, to participate in the E-diary sub-study
    - Access to smartphone/tablet and an email address
- Additionally, to participate in the Sputum sub-study
    - Chronic bronchitis, defined by self-reported sputum production for at least 3 months in each of 2 consecutive years or more.

4.2. Exclusion criteria will be:

Household member already participating in the study

5. Co-enrolment

Co-enrolment is only allowed for non-interventional studies, such as cohort studies.

6. CONSENT

It will be the responsibility of the Investigator or delegate, which may include a research nurse or other suitably trained individual (as determined by local site) who is not medically trained, to obtain written informed consent for each participant prior to performing any trial related procedure. This applies to the main study and all sub-studies. All participants will be approached for the sputum sub-study and e-diary sub-study, with enrolment for the e-diary study ceasing once 300 have consented to this aspect.

A Participant Information Sheet (PIS) will be provided to facilitate this process. Investigators or delegate(s) will ensure that they adequately explain the aim, trial intervention, anticipated benefits and potential hazards of taking part in the trial to the participant. They will also stress that participation is voluntary and that the participant is free to refuse to take part and may withdraw from the trial at any time. The participant will be given at least as much time as they need to read the PIS and to discuss their participation with others outside of the site research team.

As described in section 3.3, consent for provision of the baseline sputum sample for those participating in the sputum sub-study will initially be provided verbally, and documented by the practice team, before written informed consent is then taken at the screening appointment.

The participant will be given the opportunity to ask questions before signing and dating the latest version on the Informed Consent Form (ICF). If the participant expresses an interest in participating in the trial they will be asked to sign and date the latest version of the ICF. As direct access to participant medical records is required, the participant must give explicit consent for the regulatory authorities, members of the research team and/ or representatives of the sponsor to be given direct access to the participant's medical records.

The Investigator or delegate(s) will then sign and date the Informed Consent Form (ICF). Where appointments take place remotely (via telephone or video link) the investigator (or delegate) will initial the consent form on behalf of the participant and take a scanned copy of the ICF before forwarding it to the participant in the post (with a pre-paid self-addressed envelope) for their signature. A copy of the ICF will be given to the participant, a copy will be filed in the medical notes, and the original placed in the Investigator Site File (ISF). Once the participant is entered into the trial, the participant's trial number will be entered on the ICF, maintained in the ISF. If the participant has given explicit consent, copies of the participant's signed ICF may be requested by the BCTU trials team.

At each appointment the participant's willingness to continue in the trial will be ascertained and documented in the medical notes. Throughout the trial the participant will have the opportunity to ask questions about the trial. Any new information that may be relevant to the participant's continued participation will be provided. Where new information becomes available which may affect the participants' decision to continue, participants will be given time to consider and if happy to continue will be re-consented. Re-consent will be documented in the medical notes. The participant's right to withdraw from the trial will remain.

Electronic copies of the PIS and ICF will be available from the Trials Office and will be localised to reflect the recruiting site. Details of all participants approached about the trial will be recorded on a Colour COPD **Enrolment and screening Log** and with the participant's prior consent, their General Practitioner (GP) will also be informed that they are taking part in the trial.

7. ENROLMENT AND RANDOMISATION

7.1. Enrolment and Screening

The screening appointment will be conducted either as a face-to-face visit at the patients' GP practice or via telephone or online video link where their usual medical team will take informed consent and assess eligibility based on the study inclusion and exclusion criteria.

7.2. Randomisation

7.2.1. Randomisation Methodology

Participants will be randomised by computer (or telephone if practices have poor online access) at the level of the individual in a 1:1 ratio to either 5 point sputum colour chart, adapted from Bronkotest® colour chart or usual care as described previously, and this will be conducted by the BCTU team.

A minimisation algorithm will be used within the online randomisation system to ensure balance in the treatment allocation over the following variables, which centre on factors influencing AECOPD and admission

- Severity of COPD see Figure 1(7, 28)
    - C: CAT<10, 2 or more exacerbations in the last 12 months OR ≥1 hospital admission for an exacerbation
    - D: CAT≥10, 2 or more exacerbations in the last 12 months OR ≥1 hospital admission for an exacerbation
- Presence or absence of chronic bronchitis (57)
- Prior COPD hospitalization (yes or no within the 12 months prior to enrolment)
- Age, as defined by <65 years, 65-80 years, >80 years

In addition GP practice will be included to balance for effect of this.

A 'random element' will be included in the minimisation algorithm, so that each participant has a probability (unspecified here), of being randomised to the opposite treatment that they would have otherwise received. Full details of the randomisation specification will be stored in a confidential document at BCTU.

7.2.2. Blinding

The study is not blinded at the patient or investigator level.

7.2.3. Randomisation Process

After patient eligibility has been confirmed and informed consent has been received, the patient can be randomised into the trial. Randomisation Notepads will be provided to investigators and may be used to collate the necessary information prior to randomisation. All

questions and data items on the Randomisation Notepad must be answered before a Trial Number can be given.

Randomisation will be provided by a secure online randomisation system at the trials unit (available at https://bctu-redcap.bham.ac.uk/). Unique log-in usernames and passwords will be provided to those who wish to use the online system and who have been delegated the role of randomising participants into the study as detailed on Colour COPD Trial Signature and Delegation Log. These unique log-in details must not be shared with other staff and in no circumstances should staff at sites access either the randomisation process or trial database using another person's login details. The online randomisation system will be available 24 hours a day, 7 days a week, apart from short periods of scheduled maintenance. A telephone toll-free randomisation service (0044) 0800 953 0274) is available Monday to Friday, 09:00 to 17:00 UK time, except for Bank Holidays and University of Birmingham closed days.

7.2.4. Randomisation Records

Following randomisation, a confirmatory e-mail will be sent to the responsible clinician, randomiser, local PI, local Research Nurse, and trial co-ordinator.

Investigators will keep their own study file log which links participants with their allocated trial number in the Colour COPD Participant Recruitment and Identification Log. The Investigator must maintain this document, which is not for submission to the Trials Office. The Investigator will also keep and maintain the Colour COPD Participant Screening/Enrolment Log which will be kept in the ISF, and should be available to be sent to the Trials Office upon request. The Colour COPD Participant Recruitment and Identification Log and Colour COPD Participant Screening and Enrolment Log should be held in strict confidence.

7.3. Informing Other Parties

No other parties outside of the trial team will be routinely informed of the participants' entry into the study.

If the participant has agreed, and they are under specialist hospital follow up for their COPD, their hospital consultant should be notified that they are in Colour COPD trial, using the Colour COPD healthcare professional letter.

8. TRIAL TREATMENT / INTERVENTION

8.1. Intervention(s) and Schedule

The intervention is the 5 point sputum colour chart, adapted from Bronkotest® 5 colour sputum chart, issued alongside a standardised SM plan and rescue pack at the time of randomisation only. Instructions on how to use the intervention are available from the manufacturer and are shown briefly in the patient self-management plan, which is standardised.

8.2. Continuation after the Trial

As the 5 point sputum colour chart, adapted from Bronkotest® colour chart is issued as a one off guidance, participants are free to continue use after the study if they wish to, under the supervision of their usual care team.

9. OUTCOME MEASURES AND STUDY PROCEDURES

9.1.2. Schedule of Assessments

Pre-screening and patient approach
See sections 3.3 and 6 for details on the initial patient screening, identification and approach.

For Appointment 1 (screening) and Appointment 2 (12 month follow up) one of the following four delivery methods will be used (in order of preference, and to cover all eventualities):
1) Face to face
2) Video consultation
3) Telephone with video links sent via email to all parties to assist with the delivery of the intervention i.e. instructions on how to use the SM Plan +/- sputum colour chart
4) Telephone only (with written instructions posted to intervention participants)

Screening / Appointment 1
Potential patients identified by pre-screening will be invited to their COPD review appointment which will be conducted via one of the methods outlined above. Informed consent will be taken prior to any study assessments being conducted. As detailed in the Schedule of Assessments, Appointment 1 will be similar to the normal annual COPD review. It will include assessment of the inclusion/exclusion criteria, a review of medical history and concomitant medication, completion of CAT and EQ-5D-5L questionnaires, MRC score and lung function measurements FEV_1 and FVC, and randomisation to either intervention or best usual care. Where this appointment takes place remotely via telephone or online video link, the CAT and EQ-5D-5L questionnaires and MRC Score will be completed by reading the questions to the patient and recording their response.

Telephone Calls
A telephone call will be made to the participant by the research team at 2 weeks, 3 months, 6 months and 9 months from Appointment 1. At each time point the participant will be asked if they are happy to reconfirm consent to continue with the study .The call at 2 weeks will be to assess fidelity of the intervention. Those in the E-Diary sub-study will also have access to technical support with the app during the 2 week phone call and they will be encouraged to use the diary if they are not actively doing so by that point.
At the 3, 6, and 9 month telephone calls information regarding any acute exacerbations of COPD since last study contact will be collected and recorded. Adverse events (as defined in Section 10.0) will also be recorded and the CAT and EQ-5D-5L questionnaires will be completed by reading the questions to the patient and recording their response. At 6 months two short questionnaires measuring self-efficacy will also be completed: PROMIS "General Self-Efficacy – Short Form 4a" and PROMIS "Self-Efficacy for Managing Chronic Conditions - Managing Medications and Treatment – Short Form 8a").

Telephone calls will no longer be completed due to the withdrawal of funding by the NIHR

Appointment 2
Appointment 2 will take place 12 months after enrolment and as per Appointment 1 it will be similar to the normal annual COPD review and will take place either face-to-face at their usual GP practice, over the telephone or via an online video link. Following reconfirming consent, CAT and EQ-5D-5L questionnaires will be completed. Smoking status and concomitant medication will be recorded, together with MRC score and lung function measurements FEV_1 and FVC. Information regarding any acute exacerbations of COPD since last study contact will be collected and recorded. Adverse events (as defined in Section 10.0) will also be recorded.

Outcomes such as hospitalisations will be collected from HES (see also pilot section) as well as self-reported by the participant.

Self-reported AECOPD will be compared to that confirmed in the medical record, however the medically confirmed value will be assumed as the true number for the purpose of our secondary outcome analysis of AECOPD rate and subsequent economic evaluation.

E-diary sub-study: Patients will be approached about this sub-study at appointment 1 and if eligible and consent is obtained they will be given access to the e-diary via an app on their mobile device or tablet. At face-to face appointments a demonstration of the app will be given using instruction materials provided by the e-diary provider and a written copy of these materials will also be given to patients to take away. Where appointments take place remotely (over the telephone or video link) participants will be talked through how to set up and use the app and written instructions will be sent to them via email or post. Patients who agree to take part will be sent an email invite, including a link to the 'TakePart' app, by the central research team. They will be asked to enter a unique code which links their diary entries with their trial participant number. The fidelity call at 2 weeks post enrolment will be used to address any technical issues that arise from downloading or using the app. They will be asked to complete the e-diary on a daily basis and it will take them less than 3 minutes to enter their answers. If the patient is regularly failing to complete the e-diary, the central BCTU research team will receive an alert. Having already received consent from the patient to be contacted by BCTU, such alerts can be followed up by phone call accordingly.

Sputum sub-study: Those patients who have chronic bronchitis will be approached for the sputum sub-study. As described in section 3.3, participants that express an interest in participating in the sputum sub-study will be asked to bring a sputum sample to the screening appointment if they attend the Practice for a face-to-face visit. Consent for this element will initially be verbal, and documented by the practice team. If the patient is unable to produce sputum on that day or does not bring in a sample, they will have the option to do this at their first convenience and post the sample directly to the lab. If a patient brings a sample to the screening visit but does not enter either the sputum sub-study or the main trial, the patient and GP will have the option for this sample to be used clinically, or for it to be discarded; it will not be sent to the lab or used in trial analysis.

If the patient consents to take part in the sputum sub-study at the screening appointment, they will be provided with sputum pots and materials to post the samples in to the central lab at The University of Birmingham, together with an instruction leaflet detailing how and when to send samples. Samples will be requested at the start and end of the study, and also at AECOPD, with sputum pots being replenished via their usual care provider for any additional ones required at AECOPD. Sputum will be processed centrally at the University of Birmingham, and data transferred securely to the trials unit regularly, or immediately if a pathogen is

present and may require clinical action. The research team will contact patients with any positive results by their chosen communication method (e.g. telephone or email) and a follow up letter will also be posted to the patient and copied to their GP. Test results that do not indicate an infection will be given to patients only via telephone, letter or email. Data will be collected on sputum colour as determined by the laboratory staff against the 5 point sputum colour chart, adapted from Bronkotest®, pathogens present, and number of colony forming units/ml of each potential pathogen seen, as well as any antibiotic resistance seen on routine sensitivity testing. In addition, data relating to the participant's COPD and lung function, including routine blood results such as eosinophil count, will be extracted from existing primary care records by a member of the central research team and used in the sub-study analysis.

Trial Flowchart

**Appointment**	**Pre-screening**	**Screening Appointment 1**	**Telephone Call TC1 *(E-diary & fidelity checks)***	**Telephone Call TC2**	**Telephone Call TC3**	**Telephone Call TC4**	**End of Study Appointment 2**
**Weeks**		**Day 0**	**2 weeks**	**3 months**	**6 months**	**9 months**	**12 months**
**Time window for appointment**			**+/- 7 days**	**+/- 4 weeks**	**+/- 4 weeks**	**+/- 4 weeks**	**+/- 2 month**
Review of Inclusion & Exclusion criteria	X	X					
Informed consent		X					
Randomisation		X					
Intervention		X					
Demographics		X					
Medical history		X					
Smoking status		X					X
Concomitant medication		X					X
Educational level		X					
Chronic bronchitis		X					
FEV1 & FVC		X					X
MRC score		X					X
Trial fidelity			X				
Adverse events				X	X	X	X
CAT score		X		X	X	X	X
EQ-5D-5L		X		X	X	X	X
AECOPD rate		X		X	X	X	X
Hospitalisation review		X					X
PROMIS questionnaires					X		

Pilot Stage Outcomes

- **Progression criteria based on recruitment and data received**

  A traffic light system of green (go), amber (amend) and red (stop) is being used and summarised in the table below. If a red or amber criterion is hit the trial steering committee will be consulted. The expectation is that sites would be assisted to address amber criteria and red might trigger the trial to stop, depending on steering committee views.

Criteria	Green	Amber	Red
Recruitment rate (average per site)	>=5 per month	3-4 per month	<3 per month
% excluded due to perceived unsuitability for self-management	<30%	30-59%	≥60%
Completeness of critical data	>90%	80-90%	<80%

- **Completeness of spirometry in the GP record**

This will be described based on the ability of this to be extracted electronically from the GP record, the rate of discordance between recorded spirometry and diagnosis of COPD. If extraction is not possible, or thought to be inaccurate based on the values seen, then this route of obtaining data will not be pursued in the main trial. The data monitoring committee will make decisions on this element, in conjunction with the trial steering committee if required.

- **To determine practicality of hospital episode statistic HES data.**

We will do this by reviewing incidence of hospitalisations for AECOPD and AECOPD rate (including unreported events in the e-diary study) in the HES record and compare to self-reported events by the patient. Timeliness of obtaining data and degree of alignment to patient reported events will be used to determine if HES should continue to be used, as determined by the trial steering committee. HES has been shown to pick up more events in other respiratory conditions (specifically pneumonia) but whether this will apply in COPD is not yet clear (58).

9.2. Main Trial Outcomes

9.2.2. Primary Outcomes

A binary outcome assessing incidence of at least one AECOPD over 12 months post randomisation where patients needed hospitalisation (defined by hospital discharge letter/coding). Data will be obtained from the medical record NHS digital (hospital episode statistics; HES) which ensures any admissions not reported to the GP are picked up.

9.2.3. Secondary Outcomes

There are a number of secondary outcomes Comparisons will occur for the 12 month time point only, unless otherwise stated, and where data is collected more frequently the summed data at 12 months will be used for between arm comparisons.

- Self-reported AECOPD (including those for which admission is required) obtained by telephone calls to patients every 3 months
- Self-reported antibiotic and steroid prescriptions for AECOPD
- All cause hospital admission, from HES and/or participant self-report
- Readmissions to hospital for AECOPD at 30 and 90 days from HES and participant self-report
- Bed days due to AECOPD
- Mortality, as determined by the medical record
- Self-reported unscheduled GP visits, for AECOPD
- Self-reported prescriptions for 2nd courses of antibiotics within 14 days of self-reported event (defined as treatment failure)
- Self-reported prescriptions for anti-fungals (e.g. for oral thrush)
- Quality of life (COPD assessment test [CAT], EQ-5D-5L) at 3 monthly intervals.
    - The CAT score can range from 8 to 40 and the total score will be used to compare between groups. There are no subscales within it
    - The EQ-5D-5L generates a score from 5 to 25, the total score will be used for the economic evaluation only
- Antibiotic resistance (determined by sputum culture at baseline, all AECOPD and 12 months)
- Health Resource Usage (HRU); self-reported by participant every 3 months , and submitted using a specific HRU CRF

9.3. Participant Withdrawal and Changes of Status Within Trial

Informed consent, defined as the process of learning the key facts about a clinical trial before deciding whether or not to participate, is a continuous and dynamic process and participants will be asked about their ongoing willingness to continue participation at each appointment and telephone call.

Participants will be made aware at the beginning of the trial that they can freely withdraw (discontinue participation) from the trial at any time. A participant who withdraws from the trial does so completely (i.e. from trial treatment and all follow up) and is not willing to have any of their data, including that already collected, to be used in any future trial analysis.

A participant who wishes to cease to participate *in a particular aspect of the trial*, will be considered as having changed their status within the trial

The changes in status within trial are categorised in the following ways:

- <u>No trial intervention</u>: The participant would no longer like to receive the trial intervention, but is willing to be followed up in accordance with the schedule of assessments and if applicable using any central UK NHS bodies for long-term outcomes (i.e. the participant has agreed that data can be collected and used in the trial analysis)

- <u>No trial related follow-up</u>: The participant would no longer like to receive the trial intervention AND does not wish to attend trial appointments in accordance with the schedule of assessments but is willing to be followed up at standard clinical appointments and if applicable using any central UK NHS bodies for long-term outcomes (i.e. the participant has agreed that data can be collected at standard clinical

appointments and used in the trial analysis, including data collected as part of long-term outcomes)

- No further data use: The participant would no longer like to receive the trial intervention AND is not willing to be followed up in any way for the purposes of the trial AND does not wish for any further data to be collected (i.e. only data collected prior to the withdrawal can be used in the trial analysis)

The details of either withdrawal or change of status within trial (date, reason and category of status change) will be clearly documented in the source data.

With regard to withdrawal:

- If participants, in the view of their local care provider, lose capacity to self-manage their COPD, they will be withdrawn from the study. They may also withdraw for other reasons, and if so these will be recorded.
- If subjects withdraw from the study, reason for withdrawal will be recorded, where provided, and whether the patient consents to use of already collected data and subsequent extraction of usual care data via NHS digital. A specific CRF for this purpose will be used by trial staff
- No plans to replace withdrawn subjects have been made, as the sample size calculation allows for an anticipated rate of withdrawal
- Follow up of subjects that have withdrawn from the trial will be through their usual care practitioner, and data extracted via NHS digital for study outcomes as per the protocol, unless consent for this activity has also been withdrawn
- All patients who consent to use of their data will be analysed as per intention to treat analysis in the main study report. They would not, however, be eligible for a per protocol analysis
- Participants who are subsequently found to be ineligible will be withdrawn from the trial.

10.3.1 Patient Mortality

Participant deaths must be reported to the BCTU team promptly using the Trial exit/change of status CRF to avoid the central research team inappropriately contacting the deceased participant's family. A monthly notification will be sent to a nominated practice contact as a reminder to let the trials team know if a Colour COPD participant in their practice dies. In addition, an alert will be added onto participant's medical records to inform Practice staff that these patients are taking part in the Colour COPD Trial and to contact the Trials Team in the event of the patient's death.

ADVERSE EVENT REPORTING

10.0 Definitions

The following definitions apply:

**Adverse Event**	AE	Any untoward medical occurrence in a participant or clinical trial subject participating in the trial which does not necessarily have a causal relationship with the intervention received.
**Related Event**		An event which resulted from the administration of any of the research procedures.
**Serious Adverse Event**	SAE	An untoward occurrence that:<br>• Results in death<br>• Is life-threatening*<br>• Requires hospitalisation or prolongation of existing hospitalisation<br>• Results in persistent or significant disability or incapacity<br>• Consists of a congenital anomaly/ birth defect<br>• Or is otherwise considered medically significant by the Investigator**
**Unexpected and Related Event**		An event which meets the definition of both an Unexpected Event and a Related Event
**Unexpected Event**		The type of event that is not listed in the protocol as an expected occurrence.

* The term life-threatening is defined as diseases or conditions where the likelihood of death is high unless the course of the disease is interrupted

**medical events that may not be immediately life-threatening or result in death or hospitalisation but may jeopardise the patient or may require intervention to prevent one of the other outcomes listed in the definitions above.

10.1 Adverse Event General Recording Requirements

The collection and reporting of Adverse Events (AEs) will be in accordance with the UK Policy Framework for Health and Social Care (2017) and the requirements of the Health Research Authority (HRA). Definitions of different types of AEs are listed in the table of definitions in section 10.0.

It is routine practice to record Adverse Events in the patient's medical notes and it is also recommended that this includes the documentation of the assessment of severity and seriousness and also for causality (relatedness) in relation to the intervention(s) in accordance with the protocol.

10.2 Adverse Events Reporting Requirements in Colour COPD

The safety profile for this trial population and interventions are well established so although it is recommended that the severity, seriousness and causality of all AEs should be recorded in the source data, a strategy of targeted reporting of AEs will therefore not affect the safety of participants. The reporting of only the following subset of AEs which are potentially related to antibiotic use will be reported via the CRFs, from randomisation until the End of Study (12 month follow up appointment), is consistent with the aims of the trial.

Exacerbations of COPD will not be reported as Adverse Events in this study as they are expected in this patient population and form the basis of the trial design. Adverse Events potentially related to antibiotic use are the only events we intend to capture, which will be done alongside other healthcare utilisation data pertinent to the economic analysis. Patients will be questioned about Adverse Events at the 3, 6 and 9 month telephone calls and at the 12 month follow up appointment.

10.3 Serious Adverse Advents (SAE) Reporting in Colour COPD

We are not expecting any SAEs as a result of the intervention and so the study will not require SAE reporting in an expedited manner.
Only reports of SAEs that are:

- **related** to the study (i.e. they resulted from administration of any of the research procedures) and
- **unexpected** (i.e. not listed in the protocol as an expected occurrence)

should be submitted to the REC using the Non-CTIMP safety report to REC form.

Participants with COPD may require additional treatment for an Acute Exacerbations of COPD (AECOPD) during the study period and for this reason **the following SAE would not be regarded as unexpected for the purpose of this trial:**

- Admissions for treatment of AECOPD, unless the condition is life threatening or proves fatal

Hospitalisations and deaths will still be recorded as part of the trial data set.

10.3.1 Events not requiring reporting to BCTU on an SAE form

At whatever time they occur during an individual's participation, from randomisation to end of participant follow-up, the following are not considered to be critical to evaluations of the safety of the trial

- Pre-planned hospitalisation

All events which meet the definition of serious must be recorded in the participant notes, including the causality and severity, throughout the participant's time on trial, including follow-up, but for trial purposes these events do not require reporting on the SAE Form. Such events are "safety reporting exempt".

10.4 Reporting procedure

10.4.1 Reporting procedure for Serious Adverse Events by sites

On becoming aware that a participant has experienced an SAE, the site Investigator should report the SAE to the study Sponsor in accordance with Good Clinical Practice.

To report an SAE the Investigator must complete, date and sign the trial specific BCTU SAE form. The completed form together with any other relevant, appropriately anonymised, data should be faxed, or scanned, to the BCTU trials team using one of the numbers listed below in accordance with the timelines given in section 10.3; -in accordance with the protocol for non-expedited SAEs and no later than 24 hours after first becoming aware of the event for expedited SAEs:

To report an SAE, fax the SAE Form to:
0121 414 3050
or scan and email the SAE Form to:
colourcopd@trials.bham.ac.uk

Where an SAE Form has been completed by someone other than the Investigator initially, the original SAE form will need to be countersigned by the Investigator to confirm agreement with the causality and severity assessments.

On receipt of an SAE form, the BCTU trials team will allocate each SAE a unique reference number and return this via fax or email to the site as proof of receipt. The site and the BCTU trials team should ensure that the SAE reference number is quoted on all correspondence and follow-up reports regarding the SAE and filed with the SAE in the Site File.

If the site has not received confirmation of receipt of the SAE from the BCTU or if the SAE has not been assigned a unique SAE identification number within 1 working day, the site should contact the BCTU trials team.

10.4.2 Provision of follow-up information

Following reporting of an SAE for a participant, the participants should be followed up until resolution or stabilisation of the event. Follow-up information should be provided using the SAE reference number provided by the BCTU trials team. Once the SAE has been resolved, all critical follow-up information has been received and the paperwork is complete, the final version of the original SAE form completed at site must be returned to the BCTU trials office and a copy kept in the Site File.

10.5 Assessment of relatedness

When completing the SAE form, the site PI will be asked to define the causality (relatedness) and the severity of the AE. In defining the causality the PI (or Clinically trained delegate) must consider if any concomitant events or medications may have contributed to the event and, where this is so, these events or medications should be reported on the SAE form. It is not necessary to report concomitant events or medications which do not contribute to the event.

Category	Definition	Causality
**Definitely**	There is clear evidence to suggest a causal relationship, and other possible contributing factors can be ruled out.	Related
**Probably**	There is evidence to suggest a causal relationship, and the influence of other factors is unlikely.	
**Possibly**	There is some evidence to suggest a causal relationship. However, the influence of other factors may have contributed to the event (e.g. the participant's clinical condition, other concomitant events or medication)	
**Unlikely**	There is little evidence to suggest there is a causal relationship. There is another reasonable explanation for the event (e.g. the participant's clinical condition, other concomitant events or medication).	Unrelated
**Not related**	There is no evidence of any causal relationship.	

On receipt of an SAE Form the Trials Office will forward it, with the unique reference number, to the Chief Investigator (CI) who will independently review the causality of the SAE. An SAE judged by the PI or CI to have a reasonable causal relationship with the intervention will be regarded as a related SAE (SAR). The causality assessment given by the PI will not be downgraded by the CI. If the CI disagrees with the PI's causality assessment, the opinion of both parties will be documented, and where the event requires further reporting, the opinion will be provided with the report.

10.6 Assessment of Expectedness by the CI

The CI will also assess all related SAEs for expectedness with reference to the following criteria.

Category	Definition
**Expected**	An adverse event that is consistent with known information about the trial related procedures or that is clearly defined in the relevant safety information
**Unexpected**	An adverse event that is not consistent with known information about the trial related procedures.

The CI will not overrule the severity or causality assessment given by the site Investigator but may add additional comment on these. This study is a non-CTIMP and therefore if the event is unexpected (i.e. is not defined in the protocol as an expected event) it will be classified as an Unexpected and Related SAE.

10.6.1 Protocol defined expected SAEs

The following events are expected as a consequence of the participant's clinical condition:

- Hospital admissions for treatment of AECOPD

The events below are expected as a consequence of the trial intervention

- There are no events expected as a consequence of the trial intervention

The CI will undertake review of all SAEs and may request further information from the clinical team at site for any given event(s) to assist in this.

10.7 Reporting SAEs to third parties

The independent Data Monitoring Committee (DMC) may review any SAEs at their meetings.

BCTU will report all events categorised as Unexpected and Related SAEs to the main Research Ethics Committee (REC) and Research Governance Team (RGT) at the University of Birmingham within 15 days.

The main REC and RGT will be notified immediately if a significant safety issue is identified during the course of the trial.

Details of all Unexpected and Related SAEs and any other safety issue which arises during the course of the trial will be reported to PIs. A copy of any such correspondence should be filed in the site file and TMF.

10.8 Urgent Safety Measures

If any urgent safety measures are taken, the BCTU shall immediately, and in any event no later than 3 days from the date the measures are taken, give written notice to the REC of the measures taken and the circumstances giving rise to those measures.

10.9 Monitoring pregnancies for potential Serious Adverse Events

This is a non-CTIMP and there is no identified risk of congenital anomalies or birth defects in the offspring of participants as a result of their participation in the trial.

11.0 DATA HANDLING AND RECORD KEEPING

11.1 Source Data

Source data, defined as all information in original records and certified copies of original records of clinical findings, observations, or other activities necessary for the reconstruction and evaluation of the trial, will be kept in the participants' medical notes at their usual GP practice.

A summary of the data collected in this trial and its source is summarised below.

Data	***Source***
Participant Reported Outcomes	The participant's responses as transcribed onto the electronic CRF in the REDCap database is the source data and sites will upload electronic copies of the e-CRFs to the trial record. Where this isn't possible copies will be sent from BCTU to be kept with the participant's trial record at site. In the e-diary sub-study data within the diary also constitutes a participant reported outcome as it collects their daily symptoms.
Lab results	The original lab report (which may be electronic) is the source data and will be kept and maintained, in line with normal local practice. Information will be transcribed onto the CRF.
Clinical event data	The original clinical annotation is the source data. This may be found on clinical correspondence, or electronic or paper participant records. Clinical events reported by the participant, either in or out of clinic (e.g. phone calls), must be documented in the source data.
Health Economics data	Often obtained by interview directly with the participant for transcription onto the CRF

Health status data	The responses to the questionnaires will be entered onto the Trial database and are the source data. Data will be uploaded into the participant's trial record at site. Where this isn't possible copies will be printed and sent to BCTU to be kept with the participant's trial record at site.
Recruitment	The original record of the randomisation is the source. It is held on BCTU servers as part of the randomisation and data entry system.
Drop out	Where a participant expresses a wish to withdraw, the conversation must be recorded in the source data.
e-Diary Sub-study	e-Diary

11.2 Case Report Form (CRF) Completion

A CRF is required and should be completed for each individual subject. The data held on the completed original CRFs are the sole property of the respective PIs whilst the data set as a whole is the property of the Sponsor and should not be made available in any form to third parties except for authorised representatives or appropriate regulatory authorities without written permission from the sponsor. Appropriate data sharing requests will be considered by the sponsor.

It will be the responsibility of the investigator to ensure the accuracy of all data entered in the CRFs and confirm accordingly. The Colour COPD **Site Signature & Delegation Log** will identify all those personnel with responsibilities for data collection.

The CRFs will comprise (but will NOT be limited to) the following Forms (Table 1):

Table 1: Data Collection Forms

Form Name	***Schedule for submission***
Eligibility CRF	At the point of eligibility screening
Informed Consent Form	At the point of consent
Randomisation Notepad	At the point of randomisation
Participant Contact Details Form	After consent at Baseline appointment
Baseline CRF	As soon as possible after consent
Telephone CRF	As close to the 3 month, 6 month and 9 month time point as possible
12 month CRF	As close to the 12 month follow up time point as possible

Trial Exit/Change of Status Form	At the point of change of status, withdrawal or death
SAE reporting Form	At the point of being aware of an SAE
Exacerbation log	At each follow up time point
e-Diary CRF	Two weeks after baseline appointment
Fidelity Checklist	As close to 2 weeks after enrolment as possible
Sputum sample receipt, Lab CRF and Lab Antibiotic resistance CRF (lab forms completed by lab staff)	At baseline, all AECOPD and 12 month follow up
PROMIS short form Questionnaires (self-efficacy)	6 month follow up

Data reported on each form will be consistent with the source data and any discrepancies will be explained. Staff delegated to complete CRFs will be trained initially via a site initiation meeting or by other trained members at each site to adhere to procedures for:

- CRF completion and corrections;
- Date format and partial dates;
- Time format and unknown times;
- Rounding conventions;
- Trial-specific interpretation of data fields;
- Entry requirements for concomitant medications (generic or brand names);
- Which forms to complete and when;
- What to do in certain scenarios, for example when a participant withdraws from the trial;
- Missing/incomplete data;
- Completing SAE forms and reporting SAEs; and
- Protocol and GCP non-compliances.

All missing and ambiguous data will be queried. The following guidance applies to data and partial data:

- Time format and unknown times – all times should be in accordance with the 24hr clock
- Trial-specific interpretation of data fields – where guidance is needed additional information will be supplied
- Entry requirements for concomitant medications (generic or brand names) – generic names should be used where possible
- Missing/incomplete data – should be clearly indicated – all blank fields will be queried by the trial office

In all cases it remains the responsibility of the site's PI to ensure that the CRF has been completed correctly and that the data are accurate. This will be evidenced by the signature of the site's PI on the CRF.

11.3 Participant completed Questionnaires

Validated health status questionnaires (MRC, CAT and EQ-5D-5L) will be completed by the patient in clinic or via telephone or online video link during Appointment 1 and Appointment 2. The nursing staff will oversee the completion of the questionnaires and check that no data is missing.

At telephone scheduled calls TC1, TC2 and TC3 the questionnaires will be completed by verbal interview with the member of the research team reading out the questions and asking the patient to indicate their response.

11.4 Data Management

Processes will be employed to facilitate the accuracy of the data included in the final report. These processes will be detailed in the trial specific Data Management Plan. Coding and validation will be agreed by the trial team and the trial database will be signed off once the implementation of these has been assured.

Electronic Case Report Forms will be entered online at https://bctu-redcap.bham.ac.uk/ Authorised staff at sites (and at the trials office) will require an individual secure login username and password to access this online data entry system. Those entering data will receive written work instructions on the process (a copy of which should be filed in the ISF and TMF). CRFs should be filed within the ISF.

If changes need to be made to a CRF that has already been entered and submitted on to the database, the site should contact the Colour COPD trial office so that the form can be checked out to them and an explanation of the errors entered.

Data reported on each CRF should be consistent with the source data or the discrepancies should be explained. If information is unknown, this must be clearly indicated on the CRF. Completed CRFs will be reviewed by the Colour COPD trial office for completeness. All missing and ambiguous data will be queried.

Data queries will be generated on a regular basis by Colour COPD trial office staff and reported to the site for clarification within 28 days. The process of entering data on to the database itself forms a data quality check, as ranges are put in place to ensure that only viable data values can be input. It will be the responsibility of the Principal Investigator to ensure the accuracy of all data entered in the CRFs. These responsibilities may be delegated to an appropriate member of trial site staff. Delegated tasks must be documented on a Delegation Log and signed by all those named on the list prior to undertaking applicable trial-related procedures. The Colour COPD trial Delegation Log will identify all those personnel with responsibilities for data collection.

Questionnaires completed remotely by the participants will be received by the BCTU and will be transcribed directly onto the database. Given that these are patient reported outcomes, a data query process cannot be implemented.

CRFs may be amended and the versions updated by the Colour COPD trial office after formal review, as appropriate, throughout the duration of the trial. Whilst this may not constitute a protocol amendment, new versions of the CRFs must be implemented by participating sites immediately on receipt.

11.5 Data Security

The security of the System is governed by the policies of the University of Birmingham. The University's Data Protection Policy and the Conditions of Use of Computing and Network Facilities set out the security arrangements under which sensitive data should be processed and stored. All studies at the University of Birmingham have to be registered with the Data Protection Officer and data held in accordance with the Data Protection Act. The University will designate a Data Protection Officer upon registration of the study. The Study Centre has arrangements in place for the secure storage and processing of the study data which comply with the University of Birmingham policies.

The System incorporates the following security countermeasures:

- Physical security measures: restricted access to the building, supervised onsite repairs and storages of back-up tapes/disks are stored in a fire-proof safe.
- Logical measures for access control and privilege management: including restricted accessibility, access controlled servers, separate controls used non-identifiable data etc.
- Network security measures: including site firewalls, antivirus software, separate secure network protected hosting etc.
- System Management: the System shall be developed by the BCTU Programming Team and will be implemented and maintained by the BCTU Programming Team.
- System Design: the system shall comprise of a database and a data entry application with firewalls, restricted access, encryption and role based security controls.
- Operational Processes: the data will be processed and stored within the Study Centre (University of Birmingham).
- Data processing: Statisticians will have access to anonymised data.
- System Audit: The System shall benefit from the following internal/external audit arrangements:
    - Internal audit of the system
    - Periodic IT risk assessments
- Data Protection Registration: The University of Birmingham has Data Protection Registration to cover the purposes of analysis and for the classes of data requested. The University's Data Protection Registration number is Z6195856.

Where an external provider is used (e-diary) the sponsor is responsible for ensuring that their security is adequate and in line with the above.

11.6 Archiving

Archiving will be authorised by BCTU on behalf of the Sponsor following submission of the end of trial report.

It is the responsibility of the PI to ensure all essential trial documentation and source documents (e.g. signed ICFs, Investigator Site Files, Pharmacy Files, participants' hospital notes, copies of CRFs etc.) at their site are securely retained for at least 10 years.

No documents should be destroyed without prior approval from the Trials Office.

12.0 QUALITY CONTROL AND QUALITY ASSURANCE

12.1 Site Set-up and Initiation

All PIs will be asked to sign the necessary agreements including a Site Signature & Delegation Log between the PI and the CTU, and supply a current CV and GCP certificate to BCTU. All site staff who are performing trial specific tasks are required to sign the Site Signature and Delegation Log, which details which tasks have been delegated to them by the PI.

Prior to commencing recruitment, each recruiting site will undergo a process of initiation, either a meeting or a teleconference, at which key members of the site research team are required to attend, covering aspects of the trial design, protocol procedures, adverse event reporting, collection and reporting of data and record keeping. Sites will be provided with an Investigator Site File containing essential documentation, instructions, and other documentation required for the conduct of the trial. The BCTU trials team must be informed immediately of any change in the site research team.

12.2 Monitoring

The monitoring requirements for this trial have been developed following trial specific risk assessment by BCTU and as documented in the monitoring plan.

12.3 Onsite Monitoring

Any monitoring activities will be reported to the trials team and any issues noted will be followed up to resolution. Additional on-site monitoring visits may be triggered, for example by poor CRF return, poor data quality, low SAE reporting rates, excessive number of participant withdrawals or deviations (also defined in the monitoring plan). Investigators will allow the Colour COPD trial staff access to source documents as requested. The monitoring will be conducted by trained BCTU staff.

12.4 Central Monitoring

Trials staff will check incoming ICFs and CRFs for compliance with the protocol, data consistency, missing data and timing at a frequency and intensity determined by the Data Management Plan. Sites will be sent DCFs requesting missing data or clarification of inconsistencies or discrepancies.

If the participant has given explicit consent copies of the participant's signed ICF may be requested by the BCTU trials team for in-house review when the participant is discharged from the care of the site.

12.5 Audit and Inspection

The Investigator will permit trial-related monitoring, audits, ethical review, and regulatory inspection(s) at their site, providing direct access to source data/documents. The investigator will comply with these visits and any required follow-up. Sites are also requested to notify BCTU of any relevant inspections.

12.6 Notification of Serious Breaches

A "serious breach" is a breach which is likely to effect to a significant degree

1. The safety or physical or mental integrity of the subjects of the trial; or
2. The scientific value of the trial

This study is a non-CTIMP being conducted in the UK and as such the sponsor is responsible for notifying the REC of any serious breach of the conditions and principles of GCP in connection with that trial or the protocol relating to that trial. Sites are therefore requested to notify the Trials Office of any suspected trial-related serious breach of GCP and/or the trial protocol. Where the Trials Office is investigating whether or not a serious breach has occurred sites are also requested to cooperate with the Trials Office in providing sufficient information to report the breach to the REC where required and in undertaking any corrective and/or preventive action.

Sites may be suspended from further recruitment in the event of serious and persistent non-compliance with the protocol and/or GCP, and/or poor recruitment. Any major problems identified may be reported to the Trial Management Group, Trial Steering Committee, and the REC. This includes reporting serious breaches of GCP and/or the trial protocol to the REC. A copy is sent to the University of Birmingham Clinical Research Compliance Team at the time of reporting to the REC.

13.0 END OF TRIAL DEFINITION

The end of trial will be 6 months after the last data capture. This will allow sufficient time for the completion of protocol procedures, data collection and data input. The BCTU trial team will notify the main REC and RGT within 90 days of the end of trial. Where the trial has terminated early, the Trials Office will inform the REC within 15 days of the end of trial. The Trials Office will provide them with a summary of the clinical trial report within 12 months of the end of trial.

A copy of the end of trial notification as well as the summary report will be sent to the main REC.

14.0 STATISTICAL CONSIDERATIONS

14.1 Sample Size

We have used hospitalisation rates from the clinical practice research database (CPRD) to determine event rate for our primary outcome (28). Assuming a 1-sided significance level of 2.5% and a rate of admission in each group of 65% of that in the referenced data, with a non-inferiority margin of 6 percentage points, we would need to enrol 1329 patients in each of the intervention and control groups (2658 in total) to have 90% power for determining whether the results in the usual care group were non-inferior to those in the intervention group. Assuming dropout/lost to follow-up/non-adherence rate of 10% we therefore need to recruit 2954 patients. We have selected the 6% non-inferiority margin on the basis of clinical judgment that this was a reasonable threshold for a trade-off between a decrease in hospital admissions and other desired outcomes (reduced antibiotic use and resistance) whilst also being feasible to recruit to relatively quickly. To show non-inferiority the two-sided 95% CI of the difference between hospitalisation rates should not exceed the pre-specified non-inferiority margin of 6%; the significance level set for this non-inferiority test is set at 0.025 (one-sided). A 5% or 4% non-inferiority threshold would require >4200 and >6500 patients respectively; these margins necessitate large numbers of sites which lowers feasibility unless trial duration is extended. This is undesirable as changes in COPD management and/or health service design might affect our outcomes. With our chosen non-inferiority threshold we require around 80 GP practices, assuming that we target larger practices whose list sizes are likely to have >70 eligible patients, and thus around 35 recruits.

A systematic review of non-inferiority studies, as well as a textbook of sample sizes for clinical trials suggest that a one sided test is the appropriate one to use in a study design with a clear hypothesis (59, 60). In this case we do not believe our intervention is better than the standard arm with respect to hospital admissions (which is the primary outcome specified by the brief) because data from our own systematic review of self-management in COPD has not shown any association between such interventions and reduced admission rates (23), consequently we hypothesise that the two arms will not differ in their admission rates. A non-inferiority design allows us to power appropriately to assess safety – it remains possible that use of a colour chart will prompt patients to take less treatment than usual care, and this has potential to increase admissions if done inappropriately for the clinical setting.

14.2 Analysis of Outcome Measures

A separate Statistical Analysis Plan will be produced and will provide a more comprehensive description of the planned statistical analyses. A brief outline of these analyses is given below:

The primary comparison groups will be composed of those allocated to Usual care with 5 point sputum colour chart, adapted from Bronkotest, versus those allocated to Usual care. In the first instance, all analyses will be based on the intention to treat principle, i.e. all patients will be analysed in the treatment group to which they were randomised irrespective of compliance or other protocol deviation. For this non-inferiority trial, 'Per-Protocol' (PP) analysis will also be conducted excluding patients with major violations. The rational for this is that including such patients may potentially dilute the observed difference between groups and increasing the chance of demonstrating non-inferiority. Results will be displayed as estimates and 95% confidence intervals derived from appropriate log binomial regression model / generalised linear models. Confidence intervals around observed differences will then be compared to the pre-specified non-inferiority margin (64) (65). For all outcome measures,

appropriate summary statistics will be presented by intervention group (frequency count and percentages for categorical data, mean and standard deviation for continuous data). Intervention effects will be adjusted for the minimisation variables listed in section 7.2 where possible. No adjustment for multiple comparisons will be made.

14.2.1 Primary Outcome Measure

The primary outcome: assessing incidence of at least one episode of AECOPD over 12 months that required patient hospitalisation, will be analysed using a mixed effects log binomial regression model which will be used to calculate relative risk and 95% confidence intervals, after accounting for the intervention group and the minimisation variables (detailed in section 7.2.1). All minimisation variables will be treated as fixed effects, apart from GP practice which will be included as a random effect. If this fails to converge alternative models will be use such as the Poisson regression (62) with robust standard errors to estimate the same parameters. Usual care with 5 point sputum colour chart will be considered as non-inferior to usual care as long as the upper 95% confidence limit for the absolute risk difference (treated vs usual care) was ≤6 percentage points. Further analyses of the episodes of AECOPD over 12 months will be examined using Generalised estimating equations (GEE) (63). Longitudinal data analysis using generalized linear models (66). For the primary outcome, a one-sided confidence interval will be calculated to evaluate non-inferiority and statistical significance (p-value <0.05) will confirm non-inferiority.

14.2.2 Secondary Outcome Measures

The secondary outcomes will be analysed as listed below and the 95% confidence intervals will be two-sided:

- Self-reported AECOPD

This information will be collected from the participant via telephone call made by the research team at 3, 6, 9 months post-enrolment and by the healthcare professional at the GP practice at the 12 month follow up. The evaluation self-reported AECOPD will be analysed using repeated measures techniques (GEE) for binary outcome adjusting for the intervention group, baseline recordings (if available), minimisation variables and associated relative risks will be calculated.

- Prescription for AECOPD

During the 12th month after randomisation antibiotic and steroid prescription for AECOPD taken from participant self-report measures will be summarised using descriptive statistics. Furthermore, this recorded information will be analysed using a mixed effects log binomial regression model, adjusting for the intervention group, will be used to calculate relative risk and 95% confidence intervals.

- All cause hospital admission/ Readmission to hospital for AECOPD (30 days, 90 days)/ Unscheduled GP visits for AECOPD.

The hospital admissions frequency count will be summarised using Poisson regression techniques adjusting for the intervention group, baseline recordings (if available) and minimisation variables. Associated relative risks will be calculated. Likewise the same methods will also be applied for Unscheduled GP visits for AECOPD. Furthermore, the statistical

techniques that will be applied for 'Mortality' (below) will also be taken into consideration for All cause hospital admissions.

- Bed days due to AECOPD

Total number of bed days due to AECOPD will be summarised using basic descriptive statistics (Mean[SD]). We will also consider using linear regression models to estimate differences between the two treatment groups including the minimisation variables as covariates.

- Mortality

The evaluation of mortality between treatment groups after randomisation will be analysed using survival analysis techniques: Kaplan Meier curves with log rank test. Furthermore Cox regression methods will be applied to investigate treatment group differences adjusting for baseline recordings (if available) and minimisation variables.

- Prescription for second course of antibiotics within 14 days

The evaluation of second course of antibiotics within 14 days will be analysed in a similar manner to prescription for AECOPD. A log binomial regression model, adjusting for the intervention group and minimisation variables, will be used to calculate relative risk and 95% confidence intervals.

- Prescription for anti-fungal

The Prescription for anti-fungal will be summarised between groups using descriptive statistics and will be analysed in a similar manner to prescription for AECOPD.

- QoL (COPD assessment test [CAT], EQ-5D-5L) at 3 month intervals

Received patient questionnaires (CAT; EQ-5D-5L) will be converted into scores and analysed using mixed generalised linear modelling techniques adjusting for the intervention group, baseline score (if available) and minimisation variables. Further supportive analyses be will be carried out on questionnaire responses using a repeated measures (multi-level) model incorporating all recorded scores (screening appointment and the post-treatment scores).

- Antibiotic resistance (from sputum culture at Baseline, all AECOPD and 12 months)

Data from this variable will be analysed in a similar manner to Self-reported AECOPD.

Regarding safety, the total number of patients experiencing SAEs will be given by intervention group along with a descriptive table of the events, and statistical significance will be determined by a chi-square test.

All analyses will follow a carefully documented Statistical Analysis Plan.

14.2.3 Subgroup Analyses

Subgroup analyses will take place because additional factors have been recorded and we want to investigate the impact of these factors on the primary outcome recorded, adjusting for treatment group and minimisation variables. Tests for statistical heterogeneity (e.g. by including the treatment group by subgroup interaction parameter in the statistical model) will be performed prior to any examination of effect estimate within subgroups. The results of subgroup analyses will be treated with caution and will be used for the purposes of hypothesis generation only.

Sub group analyses, as approved by the TSC are: analyses of patients with co-morbid bronchiectasis v those without, patients with asthma v those without, patients with chronic bronchitis v those without, patients hospitalized in the year prior to inclusion v those who were not hospitalized and GOLD groups A-D. We will also describe our AECOPD results with respect to blood eosinophils and classes of COPD treatment used during the study (specifically LAMA/LABA/ICS/roflumilast/carbocisteine).

14.2.4 Missing Data and Sensitivity Analyses

Every attempt will be made to collect full data to the end of the study on all study participants; it is thus anticipated that missing data will be minimal. Participants with missing primary outcome data will not be included in the primary analysis in the first instance. This presents a risk of bias, and sensitivity analyses will be undertaken to assess the possible impact of the risk. This will consist of simulating the missing responses using a multiple imputation approach. Parameters used to simulate the missing responses will include the minimisation variables, intervention group and previous response at each time point. It is not anticipated that the randomised interventions will be associated with the number of deaths. Full details will be included in the Statistical Analysis Plan.

14.2.5 Exploratory Analyses

Patient responses from two short PROMIS questionnaires measuring self-efficacy at 6 months will be summarised and presented for each treatment group using descriptive statistics (Mean[SD]).

14.3 Planned Interim Analysis

Interim analyses of safety and efficacy for presentation to the independent DMC will occur as per standard trials unit processes. Criteria for stopping or modifying the study based on this information will be ratified by the DMC. Details of the agreed plan will be written into the Statistical Analysis Plan. Further details of DMC arrangements are given in section 16.5.

14.4 Planned Final Analyses

The primary analysis for the study will occur once all participants have completed the 1 year assessment and corresponding outcome data has been entered onto the study database and validated as being ready for analysis. E-diary and sputum sub-studies are also analysed at completion of the study. This analysis will include data items up to and including the 1 year assessment and no further.

15.0 Health Economic Evaluation

The economic evaluation will assess the cost-effectiveness of the intervention versus usual care in patients with COPD. Hospital admission is the primary outcome of the clinical trial, and therefore it is important to evaluate the cost-effectiveness of the intervention based on this outcome. The National Institute for Health and Care Excellence (NICE) recommend the use of QALYs in economic evaluations to allow comparisons across different diseases and interventions. Therefore, the evaluation will take the form of i) an incremental cost-effectiveness analysis to estimate cost per hospital admission avoided and ii) an incremental cost-utility analysis to estimate cost per quality adjusted life year (QALY). Both analyses will be from an NHS perspective over 12 months follow-up using patient level data on costs and outcomes from the trial.

Data collection: Data on hospital admissions will be self-reported by the patient and collected from HES data during the pilot stage. During the internal pilot a CRF completed at study appointments with the patient will collect this data directly until adequacy of digital data is established. Resource use information will be collected on COPD-related primary care visits, visits to other health care professionals, prescribed medications, and hospital admissions (A&E, length and nature of inpatient admissions).

The cost of the intervention and the self-management pack will be determined within the trial, taking into account the cost of the sputum colour chart and use of antibiotics and steroids. Unit costs from standard UK sources, for example NHS Reference costs will be sought for all health care resource use items. In order to calculate QALYs, the EQ-5D-5L questionnaire will be administered to patients at baseline, 3, 6, 9 and 12 months. The crosswalk value set will be applied to patient responses to obtain utility scores, in line with current NICE recommendations. The more recent English value set will be used in a sensitivity analysis.

Analysis: QALYs will be calculated using responses to the EQ-5D-5L, using the "area under the curve" approach. Unit costs will be applied to all health care resource use items, and mean resource use (for each category of health care usage) and mean total costs will be calculated for all trial participants. As cost data is likely to have a skewed distribution, the nature of the distribution of costs will be explored, and if the data is not normally distributed, a non-parametric comparison of means (using bootstrapping) will be undertaken. Multiple imputation will be used to impute all missing values for the EQ-5D and total cost estimates for non-responders.

A cost consequence analysis will initially be reported, describing all the important results relating to costs and consequences (across the full range of clinical outcomes). Incremental cost-effectiveness and cost-utility analyses will then be undertaken to estimate the incremental cost per hospital admission avoided and cost per QALY gained respectively, with adjustment for baseline covariates. The robustness of the results will be explored using sensitivity analysis. This will explore uncertainties in the trial based data itself, the methods employed to analyse the data and the generalisability of the results to other settings. Cost-effectiveness acceptability curves will also be produced to reflect the probability the

intervention will be cost effective at different cost per QALY willingness to pay thresholds. A Health Economic Analysis Plan (HEAP) will be developed which will describe their plans in more detail.

16.0 TRIAL ORGANISATIONAL STRUCTURE

16.1 Sponsor

The University of Birmingham will be acting as sponsor for this study.

16.2 Coordinating Centre

The University of Birmingham Clinical Trials Unit will be the coordinating centre for this study

16.3 Trial Management Group

The Trial Management Group includes those individuals responsible for the day-to-day management of the trial, namely the CI, statistician, trial manager, data manager and co-applicants. Some co-applicants have roles specific to particular parts of the study, such as the economic or acceptability analyses; these individuals will attend TMG meetings only when required based on the agenda. The role of the group is to monitor all aspects of the conduct and progress of the trial, ensure that the protocol is adhered to and take appropriate action to safeguard participants and the quality of the trial itself. Meetings are planned to occur monthly but will vary according to the needs of the study.

16.4 Trial Steering Committee

A TSC will be created for the Colour COPD trial and meet approximately every 6 months and as required depending on the needs of the trial. The role of the TSC is to provide overall supervision for the trial on behalf of the Trial Sponsor and the Trial Funder and to ensure that the study/trial is conducted according to the guidelines for GCP, The UK Policy Framework for Health and Social Care and all relevant regulations and local policies.

Membership and duties/responsibilities are outlined in the TSC Charter. In summary, the TSC will provide overall oversight of the trial, including the practical aspects of the study, as well as ensuring that the study is run in a way which is both safe for the participants and provides appropriate feasibility data to the sponsor and investigators.

16.5 Data Monitoring Committee

Data analyses will be supplied in confidence to an independent Data Monitoring Committee (DMC), which will be asked to give advice on whether the accumulated data from the trial, together with the results from other relevant research, justifies the continuing recruitment of further participants. The DMC will operate in accordance with a trial specific charter. The DMC will meet at least annually as agreed by the Committee and documented in the Charter. More

frequent meetings may be required for a specific reason (e.g. pilot phase, SSRE) and will be recorded in minutes.

Additional meetings may be called if recruitment is much faster than anticipated and the DMC may, at their discretion, request to meet more frequently or continue to meet following completion of recruitment. An emergency meeting may also be convened if a safety issue is identified. The DMC may consider recommending the discontinuation of the trial if the recruitment rate or data quality are unacceptable or if any issues are identified which may compromise participant safety.

16.6 Finance

Colour COPD is wholly funded by an NIHR HTA grant. Collaboration agreements are in place with 2 key commercial providers of services for the study, Bronkotest® who supply the charts for use as the intervention, and Dunkker who provide services for the e-diary sub-study.

17.0 ETHICAL CONSIDERATIONS

The trial will be performed in accordance with the recommendations guiding physicians in biomedical research involving human subjects, adopted by the 18th World Medical Association General Assembly, Helsinki, Finland, 1964, amended by the 48th WMA General Assembly, Somerset West, Republic of South Africa, 1996 (website: *http://www.wma.net/en/30publications/10policies/b3/index.html*).

The trial will be conducted in accordance with the UK Policy Framework for Health and Social Care, the applicable UK Statutory Instruments, (which include the Data Protection Act 2018) and the Principles of GCP. The protocol will be submitted to and approved by the main REC prior to circulation.

Before any participants are enrolled into the trial, the PI at each site will obtain local R&D approval/assurance. Sites will not be permitted to enrol participants until written confirmation of R&D approval/assurance is received by the BCTU trials team. It is the responsibility of the PI to ensure that all subsequent amendments gain the necessary local approval. This does not affect the individual clinicians' responsibility to take immediate action if thought necessary to protect the health and interest of individual participants.

18.0 CONFIDENTIALITY AND DATA PROTECTION

Personal data recorded on all documents will be regarded as strictly confidential and will be handled and stored in accordance with the Data Protection Act 2018.

Participants will always be identified using their unique trial identification number on the Case Report Form in correspondence with the BCTU. Participants will give their explicit consent for the movement of their consent form, giving permission for BCTU to be sent a copy. This will be used to perform in-house monitoring of the consent process.

The Investigator must maintain documents not for submission to BCTU (e.g. Participant Identification Logs) in strict confidence. In the case of specific issues and/or queries from the regulatory authorities, it will be necessary to have access to the complete trial records, provided that participant confidentiality is protected.

BCTU will maintain the confidentiality of all participant's data and will not disclose information by which participants may be identified to any third party. The e-diary provider will not process identifiable information as participants will only enter a trial ID to the system, not a name or other data. Representatives of the Colour COPD trial team and sponsor may be required to have access to participant's notes for quality assurance purposes but participants should be reassured that their confidentiality will be respected at all times.

19.0 FINANCIAL AND OTHER COMPETING INTERESTS

The Chief Investigator declares that there are no ownership interests that may be related to products, services, or interventions considered for use in the trial or that may be significantly affected by the trial.

The trial has two commercial ties, Bronkotest, who developed the original colour chart, and a commercial company (Dunkker), who will develop the e-diary. The University of Birmingham has entered into a separate contract with each party to determine IP. As such, there should be no commercial conflicts arising.

At the time of writing the protocol not all sites/personnel have been identified. This information will be collected as it becomes available and documented in the Trial Master File.

20.0 INSURANCE AND INDEMNITY

The University of Birmingham has in place Clinical Trials indemnity coverage for this trial which provides cover to the University for harm which comes about through the University's, or its staff's, negligence in relation to the design or management of the trial and may alternatively, and at the University's discretion provide cover for non-negligent harm to participants. With respect to the conduct of the trial at Site and other clinical care of the participant, responsibility for the care of the participants remains with the NHS organisation responsible for the Clinical Site and is therefore indemnified through the NHS Litigation Authority. The University of Birmingham is independent of any pharmaceutical company, and as such it is not covered by the Association of the British Pharmaceutical Industry (ABPI) guidelines for participant compensation.

21.0 POST-TRIAL CARE

After the trial care will revert to the participants' usual care provider, however they may continue to use the intervention if they wish to do so under guidance from their usual care provider.

22.0 PUBLICATION POLICY

Results of this trial will be submitted for publication in a peer reviewed journal. The manuscript will be prepared by the CI or nominated member of the investigative team and authorship will be determined by BCTU's Publication Policy Guidelines.

Any secondary publications and presentations prepared by Investigators must be reviewed and approved by the TSC. Manuscripts must be submitted to the TSC in a timely fashion and in advance of being submitted for publication, to allow time for review and resolution of any outstanding issues. Authors must acknowledge that the trial was performed with the support of the NIHR and University of Birmingham. Intellectual Property rights will be addressed in the Clinical Study Site Agreement between Sponsor and site.

23.0 ACCESS TO FINAL DATA SET

Following completion of the trial, the full anonymised dataset shall remain the property of the University of Birmingham. Site investigators will not have access to the full data set and must not use, disseminate or publish any trial data without the prior written consent of the TMG and BCTU Data Sharing Committee.

24.0 REFERENCE LIST

1. Definition and classification of chronic bronchitis for clinical and epidemiological purposes. A report to the Medical Research Council by their Committee on the Aetiology of Chronic Bronchitis. Lancet. 1965;1(7389):775-9.
2. The battle for breath - the impact of lung disease in the UK. London: British Lung Foundation, 2016.
3. Chronic obstructive pulmonary disease: NICE guideline. 2010.
4. Miller MR, Quanjer PH, Swanney MP, Ruppel G, Enright PL. Interpreting lung function data using 80 percent of predicted and fixed thresholds misclassifies over 20% of patients. Chest. 2010.
5. Quanjer PH, Stanojevic S, Cole TJ, Baur X, Hall GL, Culver BH, et al. Multi-ethnic reference values for spirometry for the 3-95-yr age range: the global lung function 2012 equations. Eur Respir J. 2012;40(6):1324-43.
6. Sluga R, Smeele IJ, Lucas AE, Thoonen BP, Grootens-Stekelenburg JG, Heijdra YF, et al. Impact of switching to new spirometric reference equations on severity staging of airflow obstruction in COPD: a crosssectional observational study in primary care. Prim Care Respir J. 2014;23(1):85-91.
7. Global Initiative for Obstructive Lung Disease [Available from: www.goldcopd.com.
8. Anthonisen NR, Manfreda J, Warren CP, Hershfield ES, Harding GK, Nelson NA. Antibiotic therapy in exacerbations of chronic obstructive pulmonary disease. Annals of internal medicine. 1987;106(2):196-204.
9. Dickens JA, Miller BE, Edwards LD, Silverman EK, Lomas DA, Tal-Singer R. COPD association and repeatability of blood biomarkers in the ECLIPSE cohort. Respir Res. 2011;12:146.
10. Thomsen M, Ingebrigtsen TS, Marott JL, Dahl M, Lange P, Vestbo J, et al. Inflammatory biomarkers and exacerbations in chronic obstructive pulmonary disease. JAMA : the journal of the American Medical Association. 2013;309(22):2353-61.
11. Miravitlles M, Kruesmann F, Haverstock D, Perroncel R, Choudhri SH, Arvis P. Sputum colour and bacteria in chronic bronchitis exacerbations: a pooled analysis. Eur Respir J. 2012;39(6):1354-60.
12. Seemungal TA, Donaldson GC, Paul EA, Bestall JC, Jeffries DJ, Wedzicha JA. Effect of exacerbation on quality of life in patients with chronic obstructive pulmonary disease. American journal of respiratory and critical care medicine. 1998;157(5 Pt 1):1418-22.
13. Langsetmo L, Platt RW, Ernst P, Bourbeau J. Underreporting exacerbation of chronic obstructive pulmonary disease in a longitudinal cohort. Am J Respir Crit Care Med. 2008;177(4):396-401.
14. Leidy NK, Murray LT, Jones P, Sethi S. Performance of the EXAcerbations of chronic pulmonary disease tool patient-reported outcome measure in three clinical trials of chronic obstructive pulmonary disease. Annals of the American Thoracic Society. 2014;11(3):316-25.
15. Vijayasaratha K, Stockley RA. Reported and unreported exacerbations of COPD: analysis by diary cards. Chest. 2008;133(1):34-41.
16. Donaldson GC, Seemungal TA, Bhowmik A, Wedzicha JA. Relationship between exacerbation frequency and lung function decline in chronic obstructive pulmonary disease. Thorax. 2002;57(10):847-52.
17. Bahadori K, FitzGerald JM, Levy RD, Fera T, Swiston J. Risk factors and outcomes associated with chronic obstructive pulmonary disease exacerbations requiring hospitalization. Can Respir J. 2009;16(4):e43-9.

18. Roberts CM, Stone RA, Lowe D, Pursey NA, Buckingham RJ. Co-morbidities and 90-day outcomes in hospitalized COPD exacerbations. COPD. 2011;8(5):354-61.
19. Celli BR, Locantore N, Yates J, Tal-Singer R, Miller BE, Bakke P, et al. Inflammatory Biomarkers Improve Clinical Prediction of Mortality in Chronic Obstructive Pulmonary Disease. Am J Respir Crit Care Med. 2012;185(10):1065-72.
20. Beasley V, Joshi PV, Singanayagam A, Molyneaux PL, Johnston SL, Mallia P. Lung microbiology and exacerbations in COPD. Int J Chron Obstruct Pulmon Dis. 2012;7:555-69.
21. Majothi S, Jolly K, Heneghan NR, Price MJ, Riley RD, Turner AM, et al. Supported self-management for patients with COPD who have recently been discharged from hospital: a systematic review and meta-analysis. Int J Chron Obstruct Pulmon Dis. 2015;10:853-67.
22. Johnson-Warrington V, Rees K, Gelder C, Morgan MD, Singh SJ. Can a supported self-management program for COPD upon hospital discharge reduce readmissions? A randomized controlled trial. Int J Chron Obstruct Pulmon Dis. 2016;11:1161-9.
23. Jordan RE, Majothi S, Heneghan NR, Blissett DB, Riley RD, Sitch AJ, et al. Supported self-management for patients with moderate to severe chronic obstructive pulmonary disease (COPD): an evidence synthesis and economic analysis. Health Technol Assess. 2015;19(36):1-516.
24. Lenferink A, Brusse-Keizer M, van der Valk PD, Frith PA, Zwerink M, Monninkhof EM, et al. Self-management interventions including action plans for exacerbations versus usual care in patients with chronic obstructive pulmonary disease. Cochrane Database Syst Rev. 2017;8:CD011682.
25. Jolly K, Majothi S, Sitch AJ, Heneghan NR, Riley RD, Moore DJ, et al. Self-management of health care behaviors for COPD: a systematic review and meta-analysis. Int J Chron Obstruct Pulmon Dis. 2016;11:305-26.
26. Blackmore C, Johnson-Warrington VL, Williams JE, Apps LD, Young HM, Bourne C, et al. Development of a training program to support health care professionals to deliver the SPACE for COPD self-management program. Int J Chron Obstruct Pulmon Dis. 2017;12:1669-81.
27. Hurst JR, Vestbo J, Anzueto A, Locantore N, Mullerova H, Tal-Singer R, et al. Susceptibility to exacerbation in chronic obstructive pulmonary disease. N Engl J Med. 2010;363(12):1128-38.
28. Merinopoulou E, Raluy-Callado M, Ramagopalan S, MacLachlan S, Khalid JM. COPD exacerbations by disease severity in England. Int J Chron Obstruct Pulmon Dis. 2016;11:697-709.
29. Estimating the economic burden of respiratory illness in the UK. London: British Lung Foundation, 2016.
30. Stone RA, Holzhauer BJ, McMillan V, Saleem Khan M, Searle L, Skipper E, et al. COPD: Who cares when it matters most? London: Royal College of Physicians, 2017.
31. Steer J, Gibson GJ, Bourke SC. Longitudinal change in quality of life following hospitalisation for acute exacerbations of COPD. BMJ open respiratory research. 2015;2(1):e000069.
32. Vijayasaratha K, Stockley RA. Relationship between frequency, length, and treatment outcome of exacerbations to baseline lung function and lung density in alpha-1 antitrypsin-deficient COPD. Int J Chron Obstruct Pulmon Dis. 2012;7:789-96.
33. Albert RK, Connett J, Bailey WC, Casaburi R, Cooper JA, Jr., Criner GJ, et al. Azithromycin for prevention of exacerbations of COPD. N Engl J Med. 2011;365(8):689-98.
34. Fighting antibiotic resistance: NHS Improvement; 2017 [Available from: https://improvement.nhs.uk/resources/fighting-antimicrobial-resistance/.
35. Rodrigo-Troyano A, Suarez-Cuartin G, Peiro M, Barril S, Castillo D, Sanchez-Reus F, et al. Pseudomonas aeruginosa resistance patterns and clinical outcomes in hospitalized exacerbations of COPD. Respirology. 2016;21(7):1235-42.

36. Stockley RA, O'Brien C, Pye A, Hill SL. Relationship of sputum color to nature and outpatient management of acute exacerbations of COPD. Chest. 2000;117(6):1638-45.
37. Stone RA, Holzhauer BJ, Lowe D, Searle L, Skipper E, Welham S, et al. National COPD audit programme. COPD: Who cares matters. London, UK: Royal College of Physicians, 2015 Contract No.: eISBN 978-1-86016-559-7.
38. Woodcock A, Boucot I, Leather DA, Crawford J, Collier S, Bakerly ND, et al. Effectiveness versus efficacy trials in COPD: how study design influences outcomes and applicability. Eur Respir J. 2018;51(2).
39. O'Brien C, Guest PJ, Hill SL, Stockley RA. Physiological and radiological characterisation of patients diagnosed with chronic obstructive pulmonary disease in primary care. Thorax. 2000;55(8):635-42.
40. John C, Soler Artigas M, Hui J, Nielsen SF, Rafaels N, Pare PD, et al. Genetic variants affecting cross-sectional lung function in adults show little or no effect on longitudinal lung function decline. Thorax. 2017;72(5):400-8.
41. Pillai AP, Turner AM, Stockley RA. Global Initiative for Chronic Obstructive Lung Disease 2011 symptom/risk assessment in alpha1-antitrypsin deficiency. Chest. 2013;144(4):1152-62.
42. Woolhouse IS, Hill SL, Stockley RA. Symptom resolution assessed using a patient directed diary card during treatment of acute exacerbations of chronic bronchitis. Thorax. 2001;56(12):947-53.
43. Allegra L, Blasi F, Diano P, Cosentini R, Tarsia P, Confalonieri M, et al. Sputum color as a marker of acute bacterial exacerbations of chronic obstructive pulmonary disease. Respir Med. 2005;99(6):742-7.
44. Cleveland color of snot chart 2017 [Available from: https://health.clevelandclinic.org/2014/11/whatthe-color-of-your-snot-really-means/.
45. Stolbrink M, Amiry J, Blakey JD. Does antibiotic treatment duration affect the outcomes of exacerbations of asthma and COPD? A systematic review. Chron Respir Dis. 2017:1479972317745734.
46. Khan A, Dickens AP, Adab P, Jordan RE. Self-management behaviour and support among primary care COPD patients: cross-sectional analysis of data from the Birmingham Chronic Obstructive Pulmonary Disease Cohort. Prim Care Respir Med. 2017;27.
47. British Lung Foundation COPD resources 2018 [Available from: https://shop.blf.org.uk/products/copd-self-management-plan.
48. Avery KN, Williamson PR, Gamble C, O'Connell Francischetto E, Metcalfe C, Davidson P, et al. Informing efficient randomised controlled trials: exploration of challenges in developing progression criteria for internal pilot studies. BMJ open. 2017;7(2):e013537.
50. Leidy NK, Wilcox TK, Jones PW, Roberts L, Powers JH, Sethi S. Standardizing measurement of chronic obstructive pulmonary disease exacerbations. Reliability and validity of a patient-reported diary. Am J Respir Crit Care Med. 2011;183(3):323-9.
51. Izquierdo-Alonso JL, Rodriguez-Gonzalezmoro JM, de Lucas-Ramos P, Unzueta I, Ribera X, Anton E, et al. Prevalence and characteristics of three clinical phenotypes of chronic obstructive pulmonary disease (COPD). Respir Med. 2013;107(5):724-31.
52. Wood AM, Bassford C, Webster D, Newby P, Rajesh P, Stockley RA, et al. Vitamin D-binding protein contributes to COPD by activation of alveolar macrophages. Thorax. 2011.
53. Gompertz S, O'Brien C, Bayley DL, Hill SL, Stockley RA. Changes in bronchial inflammation during acute exacerbations of chronic bronchitis. Eur Respir J. 2001;17(6):1112-9.
54. Gozzelino R, Arosio P. Iron Homeostasis in Health and Disease. International journal of molecular sciences. 2016;17(1).

56. Gale NK, Heath G, Cameron E, Rashid S, Redwood S. Using the framework method for the analysis of qualitative data in multi-disciplinary health research. BMC medical research methodology. 2013;13:117.
57. Foreman MG, DeMeo DL, Hersh CP, Reilly JJ, Silverman EK. Clinical determinants of exacerbations in severe, early-onset COPD. Eur Respir J. 2007;30(6):1124-30.
58. Busch R, Cho MH, Silverman EK. Progress in disease progression genetics: dissecting the genetic origins of lung function decline in COPD. Thorax. 2017;72(5):389-90.
59. Rehal S, Morris TP, Fielding K, Carpenter JR, Phillips PP. Non-inferiority trials: are they inferior? A systematic review of reporting in major medical journals. BMJ open. 2016;6(10):e012594.
60. Machin D, Campbell NJ, Tan SB, Tan SH. Sample Size Tables for Clinical Trials: Wiley-Blackwell; 2009.
61. Schneider S, Schmidli H, Friede T. Blinded and unblinded internal pilot study designs for clinical trials with count data. Biometrical journal Biometrische Zeitschrift. 2013;55(4):617-33.

62. Zou G. A modified poisson regression approach to prospective studies with binary data. American Journal of Epidemiology. 2004;159:702-706
63. Liang, K.-Y., & Zeger, S. L. (1986).
64. Fleming TR, Current issues in non-inferiority trials. Stat Med. 2008;27(3):317-332
65. Macaya F et al, Challenges in the design and interpretation of noninferiority trials. JACC. 2017;70(7))
66. Biometrika,73, 13-22.

25.0 APPENDICES

Your COPD Action Plan

Appendix 1

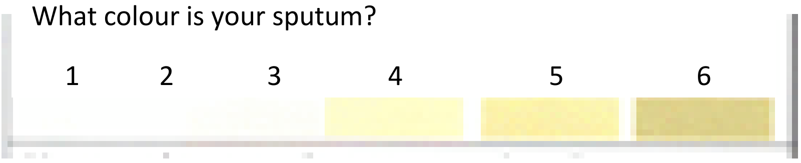

5 point sputum colour chart, adapted from Bronkotest® This will be a credit card shape and size for the purposes of the trial.

Appendix 2

Standardised self-management (SM) plan –Both groups

Staying well

Keep warm! Your living room temperature should be around 21degrees and your bedroom between 16 and 18 degrees.

Keep active! Many people with COPD reduce their activity levels to avoid becoming breathless. But if you do this you will become less fit and more breathless when you are active. Ask your GP or nurse about Better Breathing activity classes for COPD.

Eat a well-balanced diet with plenty of fresh fruit and vegetables. Try to keep good sleeping habits.

Have a flu vaccination every year and a pneumonia jab at least once.

Stopping smoking is the most important thing you can do to prevent your COPD getting worse. It is never too late to quit. Ask your doctor or nurse about how you can get help to stop smoking.

Take your medicines regularly as prescribed. Make sure that your doctor or nurse has checked that you can use your inhalers correctly.

Even if you are well make sure you see your practice nurse at least once a year for a COPD check up

You can get more information, help, advice and support for COPD from The British Lung Foundation or your local Breathe Easy support group
HELPLINE 08458 50 50 20 or ONLINE at www.lunguk.org
Breathe Easy – [sites to insert local details]

How to treat flare-ups of your COPD using your rescue pack

This plan will help you: Learn how to identify and manage flare-ups (exacerbations). The earlier you spot a flare up and step up your treatment the better.

Look out for these symptoms and follow the plan inside this booklet

More coughing than usual

More breathless than usual

More phlegm than usual

Phlegm changing colour to dark yellow/ green or brown

How to treat flare-ups of your COPD using your rescue pack

More breathlessness, wheeze, cough or phlegm than usual for at least 2 days? →

- Increase your blue reliever inhaler to 4 puffs every 2-4 hours
- OR If you have a nebuliser for your reliever use it every 4 hours
- If this does not help OR you are much worse than normal take 30mg prednisolone (6 x 5mg tablets) every morning for 5 days AND a 5 day course of antibiotics, as issued in your rescue pack

Contact your GP practice to arrange for a COPD review and to re-stock your rescue antibiotic and steroid tablets.

Feeling much worse or not improving? →

- If your symptoms are getting worse despite treatment or you are struggling to cope at home **CALL YOUR GP**

Very severe symptoms →

- If you develop chest pain, sudden very severe breathlessness, confusion or are feeling very drowsy **SPEAK TO YOUR GP URGENTLY**

Standardised self-management (SM) plan – Intervention group

More breathlessness or wheeze than usual for at least 2 days?

- Increase your blue reliever inhaler to 4 puffs every 2-4 hours
- OR If you have a nebuliser for your reliever use it every 4 hours
- If this does not help OR you are much worse than normal take 30mg prednisolone (6 x 5mg tablets) every morning for 5 days.

More cough and phlegm than usual for at least 2 days?

- Cough onto a white tissue
- Use your sputum colour chart to help you decide if you need antibiotics

Contact your GP practice to arrange for a COPD review and to re-stock your rescue antibiotic and steroid tablets.

Feeling much worse or not improving?

- If your symptoms are getting worse despite treatment or you are struggling to cope at home **CALL YOUR GP**

Very severe symptoms

- If you develop chest pain, sudden very severe breathlessness, confusion or are feeling very drowsy **SPEAK TO YOUR GP URGENTLY**

Remember ambulances and A&E are for life threatening emergencies only

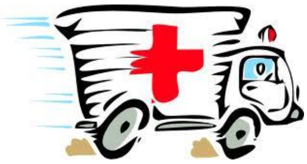

Appendix 3

PROMIS® Item Bank v1.0 – General Self Efficacy – Short Form 4a 1 September 2017 © 2008-2017 PROMIS Health Organization (PHO) Page 1 of 1

PROMIS® Item Bank v1.0 – General Self Efficacy – Short Form 4a

General Self-Efficacy – Short Form 4a

Please respond to each item by marking one box per row.

For the next set of questions, please read each sentence and rate your level of confidence in managing various situations, problems, and events.

	**Rate your level of confidence.**	**I am not at all confident**	**I am a little confident**	**I am somewhat confident**	**I am quite confident**	**I am very confident**
GSE11_C	I can manage to solve difficult problems if I try hard enough.	☐ 1	☐ 2	☐ 3	☐ 4	☐ 5
GSE14_C	I am confident that I could deal efficiently with unexpected events.	☐ 1	☐ 2	☐ 3	☐ 4	☐ 5
GSE19_C	If I am in trouble, I can think of a solution.	☐ 1	☐ 2	☐ 3	☐ 4	☐ 5
GSE20_C	I can handle whatever comes my way........	☐ 1	☐ 2	☐ 3	☐ 4	☐ 5

PROMIS Item Bank v1.0 - Self-Efficacy for Managing Chronic Conditions - Managing Medications and Treatment - Short Form 8a
Last Updated: 21 June 2017© 2010-2017 PROMIS Health Organization (PHO) Page 1 of 1

PROMIS Item Bank v1.0 - Self-Efficacy for Managing Chronic Conditions - Managing Medications and Treatment - Short Form 8a

Self-Efficacy for Managing Chronic Conditions - Managing Medications and Treatment – Short Form 8a

Please respond to each question or statement by marking one box per row.

	CURRENT level of confidence…	I am not at all confident	I am a little confident	I am somewhat confident	I am quite confident	I am very confident
SEMMT005	I can follow directions when my doctor changes my medications............................	☐ 1	☐ 2	☐ 3	☐ 4	☐ 5
SEMMT019	I can take my medication when I am working or away from home	☐ 1	☐ 2	☐ 3	☐ 4	☐ 5
SEMMT023	I can take my medication when there is a change in my usual day (unexpected things happen)	☐ 1	☐ 2	☐ 3	☐ 4	☐ 5
SEMMT006	I can manage my medication without help	☐ 1	☐ 2	☐ 3	☐ 4	☐ 5
SEMMT002	I can remember to take my medication as prescribed	☐ 1	☐ 2	☐ 3	☐ 4	☐ 5
SEMMT017	I can use technology to help me manage my medication and treatments (for example: to get information, avoid side-effects, schedule reminders)	☐ 1	☐ 2	☐ 3	☐ 4	☐ 5
SEMMT010	I can list my medications, including the doses and schedule	☐ 1	☐ 2	☐ 3	☐ 4	☐ 5
SEMMT024	I can figure out what treatment I need when my symptoms change	☐ 1	☐ 2	☐ 3	☐ 4	☐ 5
